# Supplementary material for: Biosynthesis of ilamycins featuring unusual building blocks and engineered production of enhanced anti-tuberculosis agents
Source: Nat Commun. 2017 Aug 30;8:391. doi: 10.1038/s41467-017-00419-5 (PMC5577134; doi:10.1038/s41467-017-00419-5)
Supplement: Supplementary file 1 — Supplementary Information [file 41467_2017_419_MOESM1_ESM.pdf]

Description of Supplementary Files

File Name: Supplementary Information
Description: Supplementary Figures, Supplementary Tables and Supplementary References

File Name: Supplementary Data 1
Description: The CIF file of crystallographic data for ilamycin B_2 (2)

File Name: Supplementary Data 2
Description: The CIF file of crystallographic data for ilamycin C_2 (4)

File Name: Supplementary Data 3
Description: The CIF file of crystallographic data for ilamycin D (5)

File Name: Supplementary Data 4
Description: The CIF file of crystallographic data for ilamycin F (8)

File Name: Supplementary Data 5
Description: The check CIF file of crystallographic data for ilamycin B_2 (2), ilamycin C_2 (4), ilamycin D (5) and ilamycin F (8).

File Name: Peer Review File

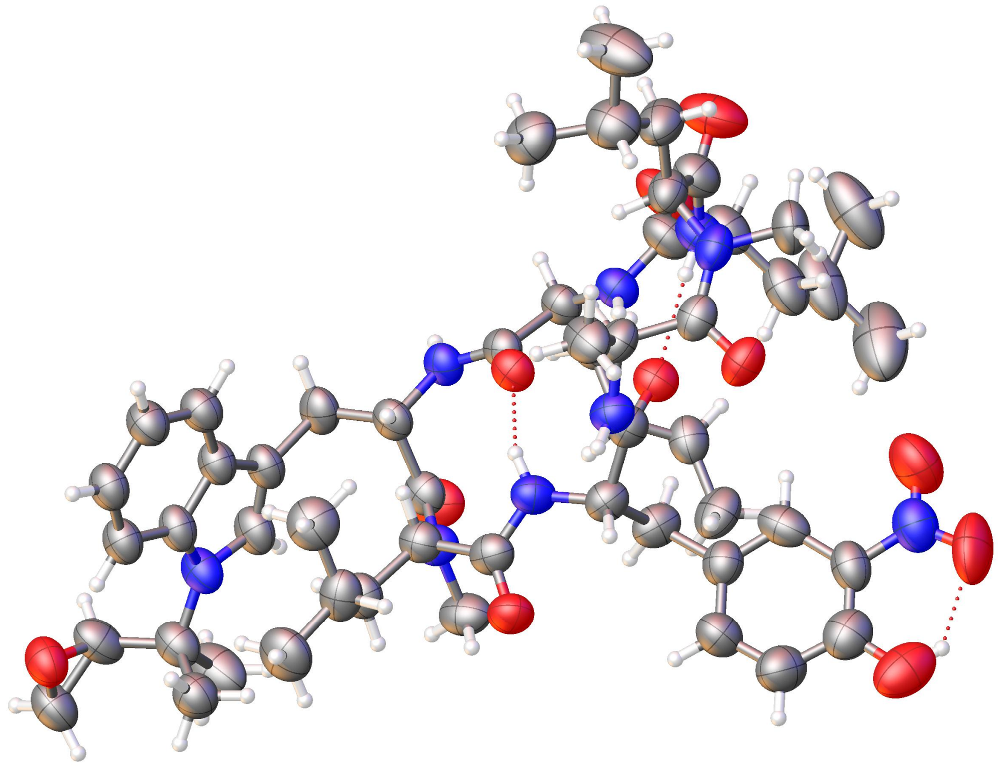

Supplementary Figure 1. The prespective view of the X-ray crystal structure of ilamycin B_2 (**2**).

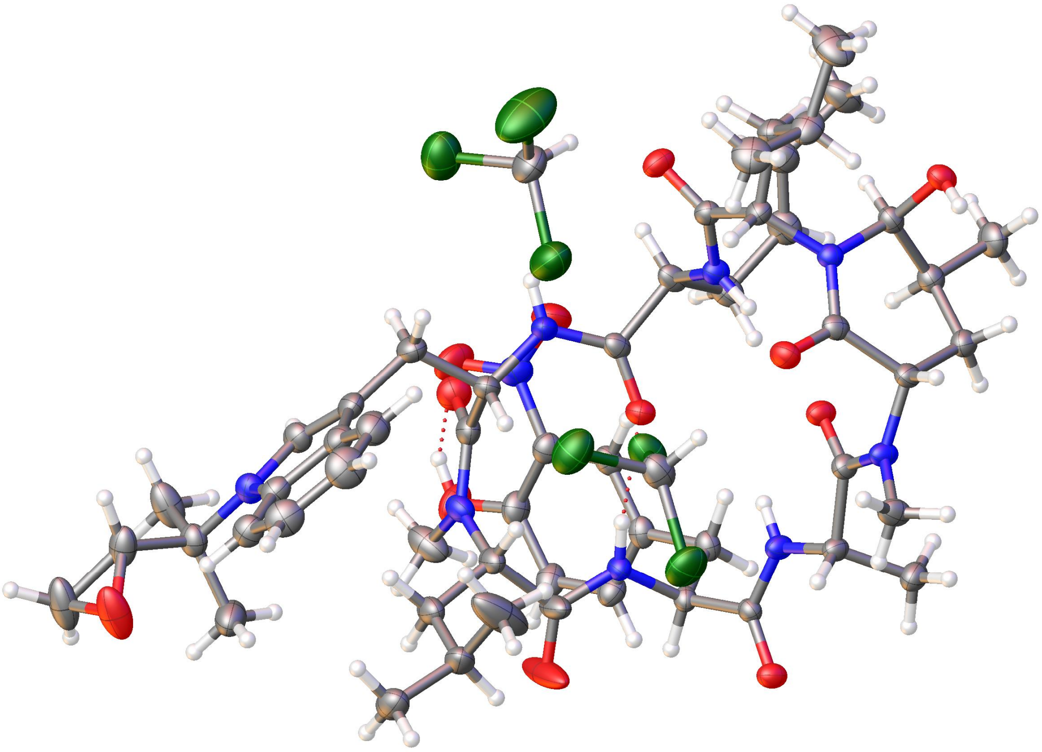

Supplementary Figure 2. The prespective view of the X-ray crystal structure of ilamycin C_2 (**4**).

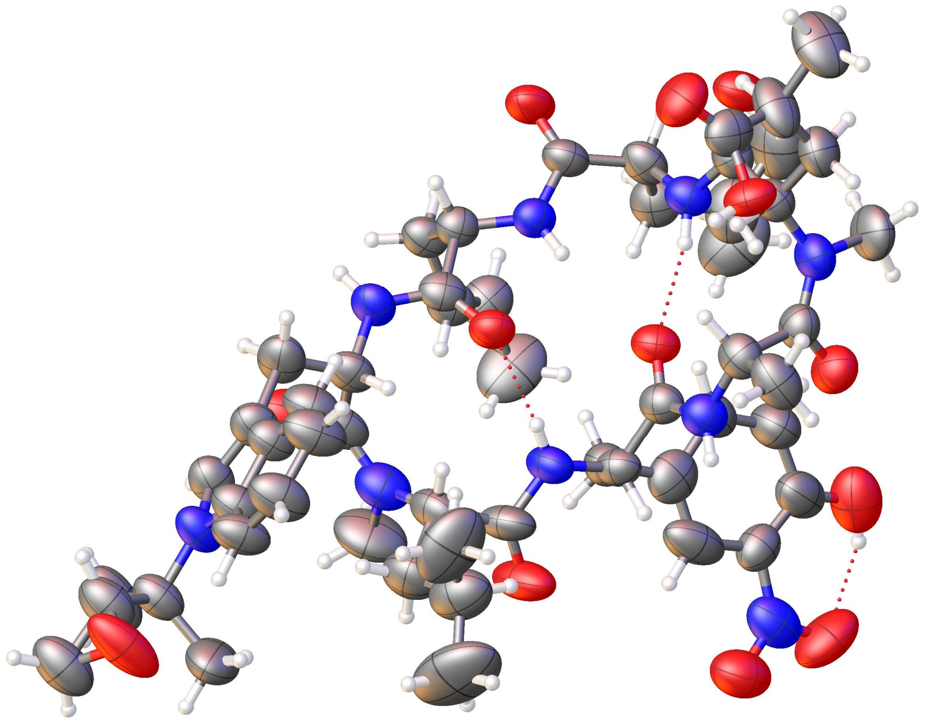

Supplementary Figure 3. The prespective view of the X-ray crystal structure of ilamycin D (**5**).

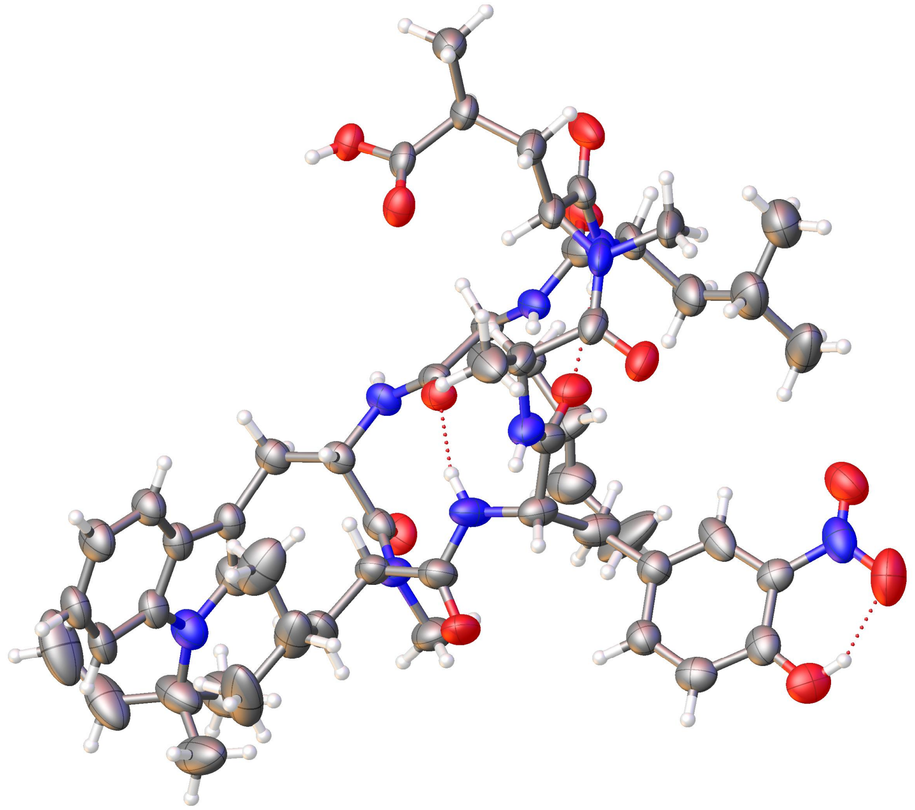

Supplementary Figure 4. The prespective view of the X-ray crystal structure of ilamycin F (**8**).

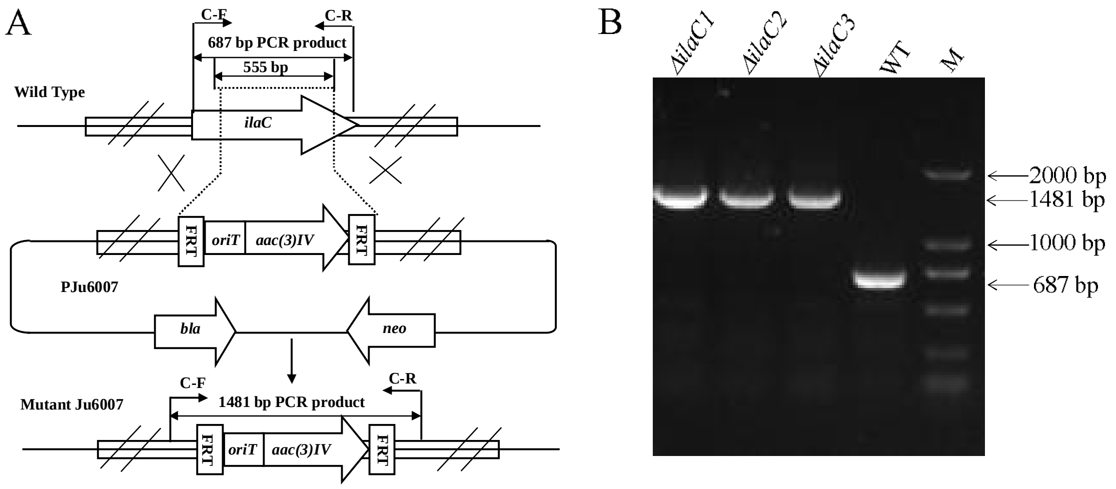

Supplementary Figure 5. Disruption of *ilaC* in wild-type *S. atratus* SCSIO ZH16 via PCR-targeting. (A) Schematic representation for disruption of *ilaC.* (B*)* PCR analyses of the wild-type strain and the *ilaC* double-cross mutant carried out using the primers listed in Supplementary Table 5. M: DNA molecular ladder; WT: using the genomic DNA of *S. atratus* SCSIO ZH16 as template; *ΔilaC1-3*: using the genomic DNA of *ilaC* mutant as template.

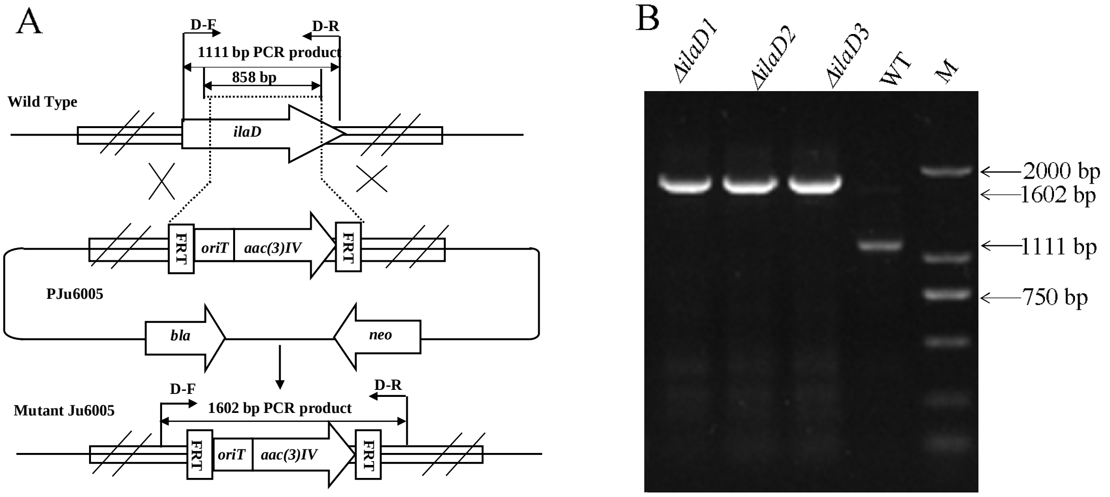

Supplementary Figure 6. Disruption of *ilaD* in wild-type *S. atratus* SCSIO ZH16 via PCR-targeting. (A) Schematic representation for disruption of *ilaD.* (B*)* PCR analyses of the wild-type strain and the *ilaD* double-cross mutant carried out using the primers listed in Supplementary Table 5. M: DNA molecular ladder; WT: using the genomic DNA of *S. atratus* SCSIO ZH16 as template; *ΔilaD1-3*: using the genomic DNA of *ilaD* mutant as template.

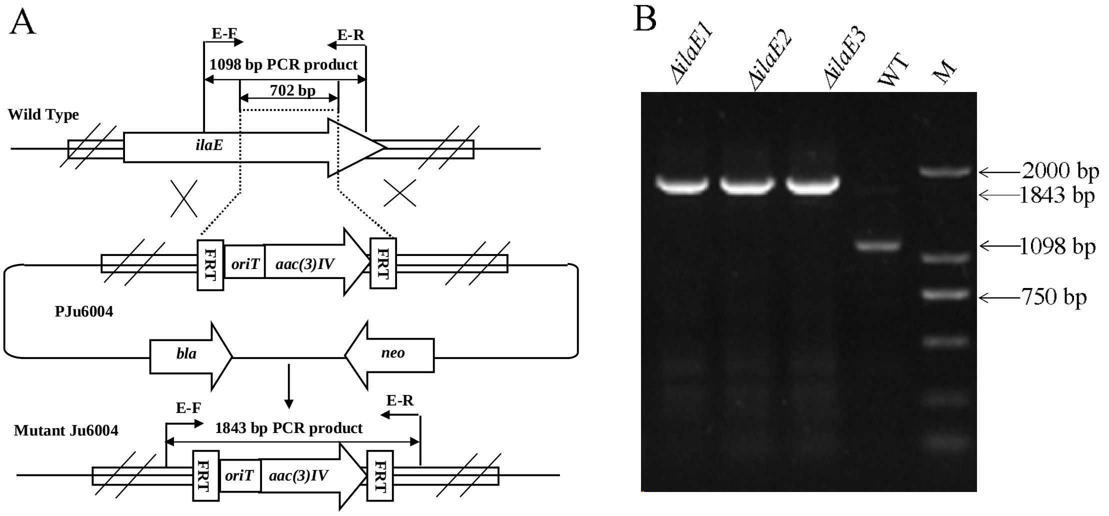

Supplementary Figure 7. Disruption of *ilaE* in wild-type *S. atratus* SCSIO ZH16 via PCR-targeting. (A) Schematic representation for disruption of *ilaE.* (B*)* PCR analyses of the wild-type strain and the *ilaE* double-cross mutant carried out using the primers listed in Supplementary Table 5. M: DNA molecular ladder; WT: using the genomic DNA of *S. atratus* SCSIO ZH16 as template; *ΔilaE1-3*: using the genomic DNA of *ilaE* mutant as template.

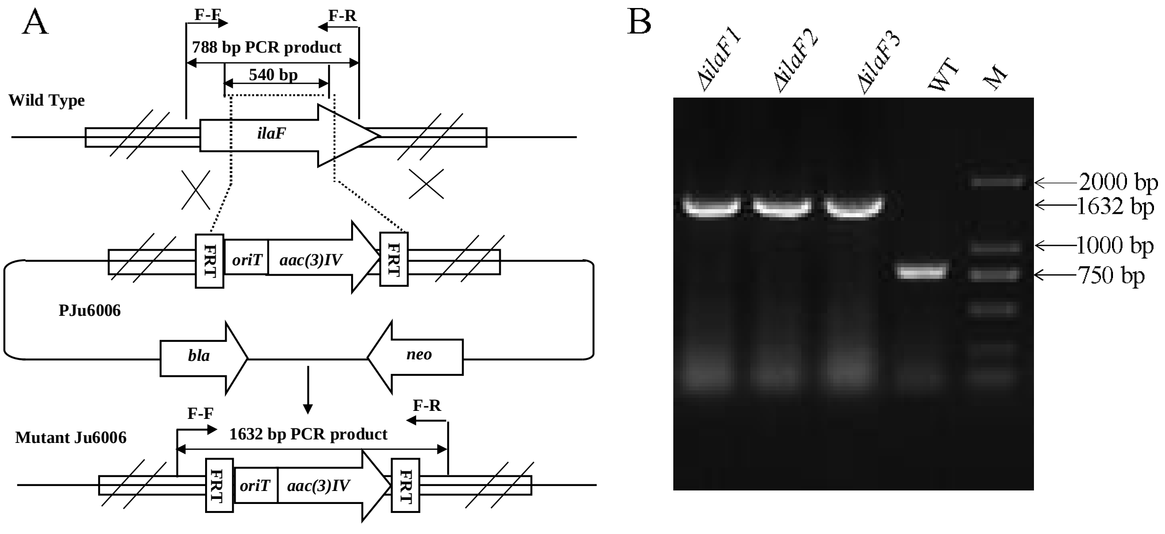

Supplementary Figure 8. Disruption of *ilaF* in wild-type *S. atratus* SCSIO ZH16 via PCR-targeting. (A) Schematic representation for disruption of *ilaF.* (B*)* PCR analyses of the wild-type strain and the *ilaF* double-cross mutant carried out using the primers listed in Supplementary Table 5. M: DNA molecular ladder; WT: using the genomic DNA of *S. atratus* SCSIO ZH16 as template; *ΔilaF1-3*: using the genomic DNA of *ilaF* mutant as template.

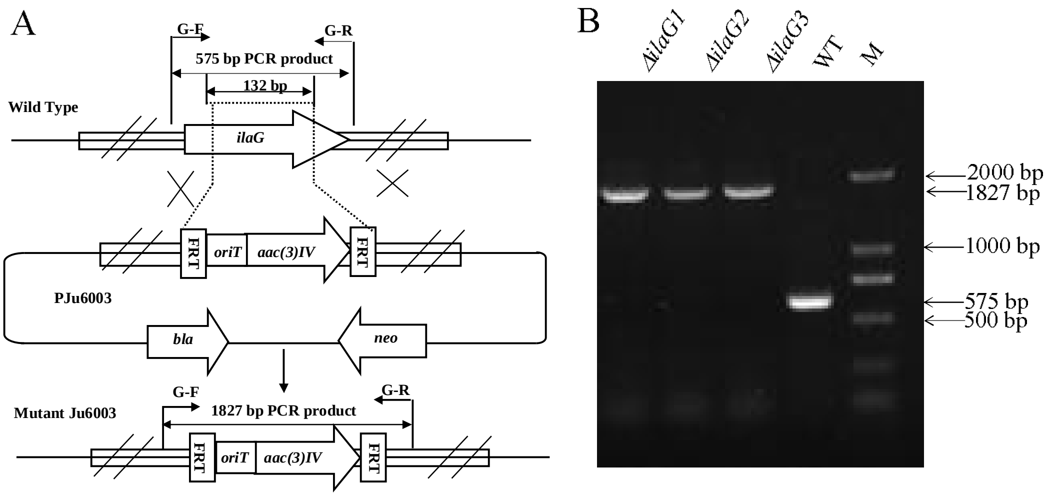

Supplementary Figure 9. Disruption of *ilaG* in wild-type *S. atratus* SCSIO ZH16 via PCR-targeting. (A) Schematic representation for disruption of *ilaG*. (B*)* PCR analyses of the wild-type strain and the *ilaG* double-cross mutant carried out using the primers listed in Supplementary Table 5. M: DNA molecular ladder; WT: using the genomic DNA of *S. atratus* SCSIO ZH16 as template; *ΔilaG1-3*: using the genomic DNA of *ilaG* mutant as template.

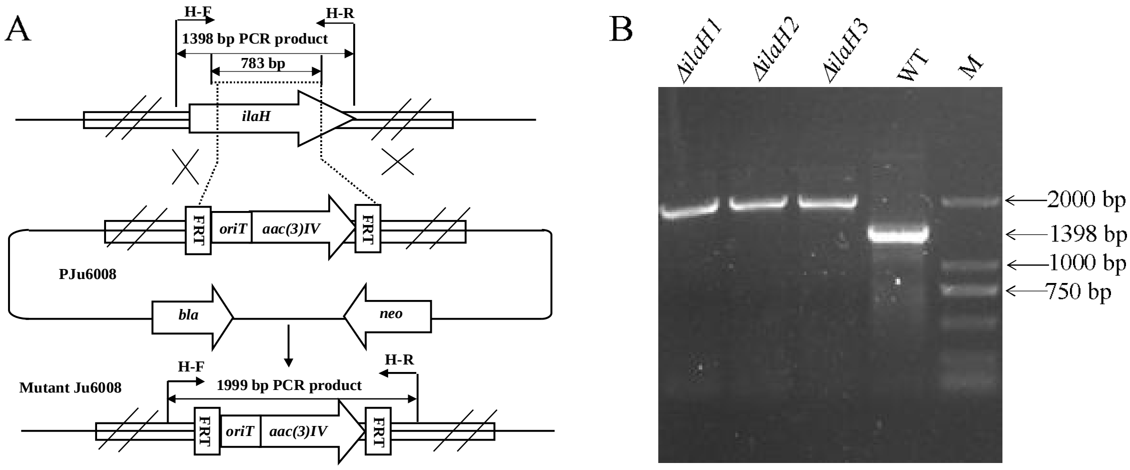

Supplementary Figure 10. Disruption of *ilaH* in wild-type *S. atratus* SCSIO ZH16 via PCR-targeting. (A) Schematic representation for disruption of *ilaH.* (B*)* PCR analyses of the wild-type strain and the *ilaH* double-cross mutant carried out using the primers listed in Supplementary Table 5. M: DNA molecular ladder; WT: using the genomic DNA of *S. atratus* SCSIO ZH16 as template; *ΔilaH1-3*: using the genomic DNA of *ilaH* mutant as template.

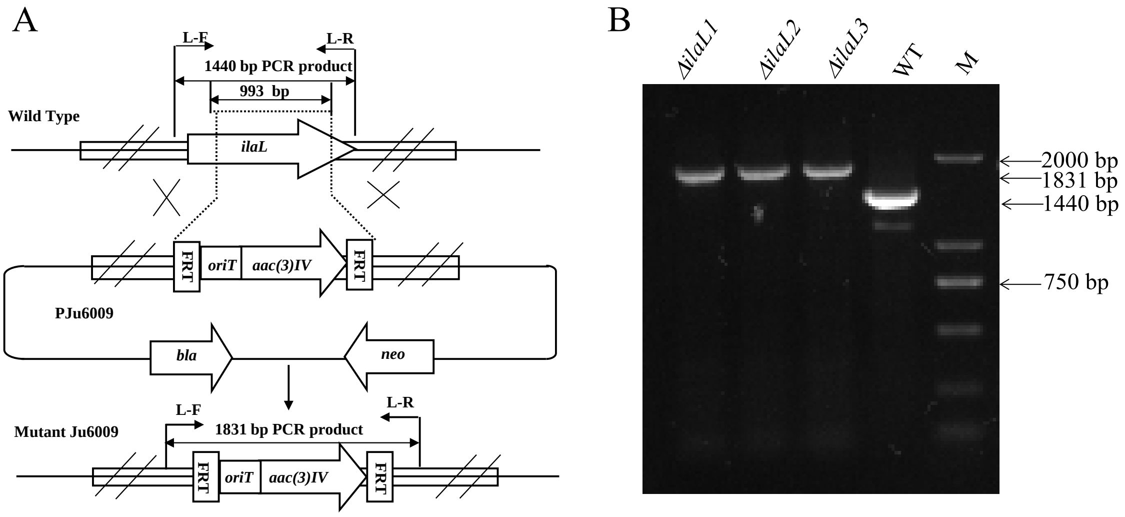

Supplementary Figure 11. Disruption of *ilaL* in wild-type *S. atratus* SCSIO ZH16 via PCR-targeting. (A) Schematic representation for disruption of *ilaL.* (B*)* PCR analyses of the wild-type strain and the *ilaL* double-cross mutant carried out using the primers listed in Supplementary Table 5. M: DNA molecular ladder; WT: using the genomic DNA of *S. atratus* SCSIO ZH16 as template; *ΔilaL1-3*: using the genomic DNA of *ilaL* mutant as template.

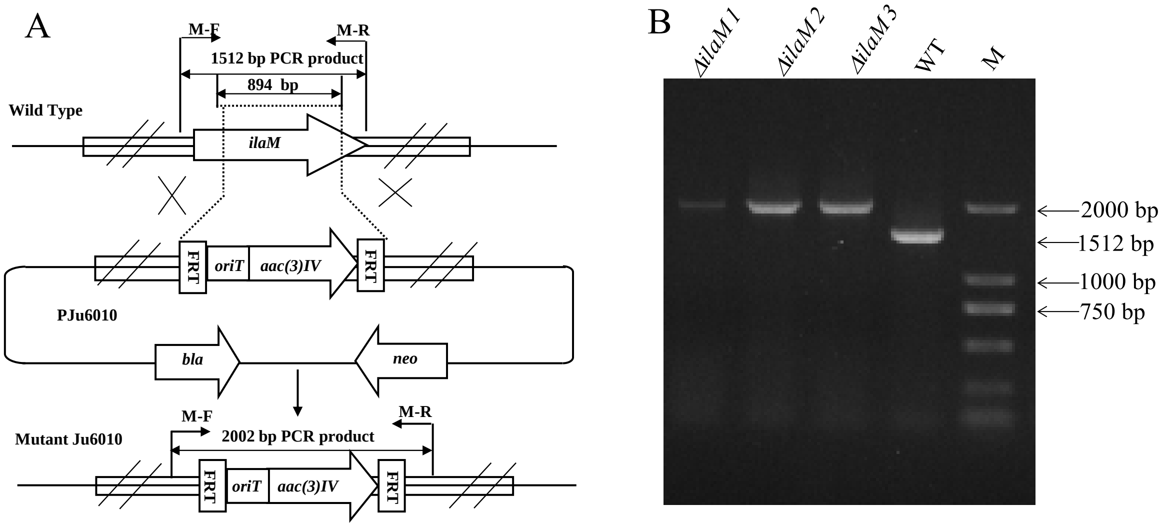

Supplementary Figure 12. Disruption of *ilaM* in wild-type *S. atratus* SCSIO ZH16 via PCR-targeting. (A) Schematic representation for disruption of *ilaM.* (B) PCR analyses of the wild-type strain and the *ilaM* double-cross mutant carried out using the primers listed in Supplementary Table 5. M: DNA molecular ladder; WT: using the genomic DNA of *S. atratus* SCSIO ZH16 as template; *ΔilaM1-3*: using the genomic DNA of *ilaM* mutant as template.

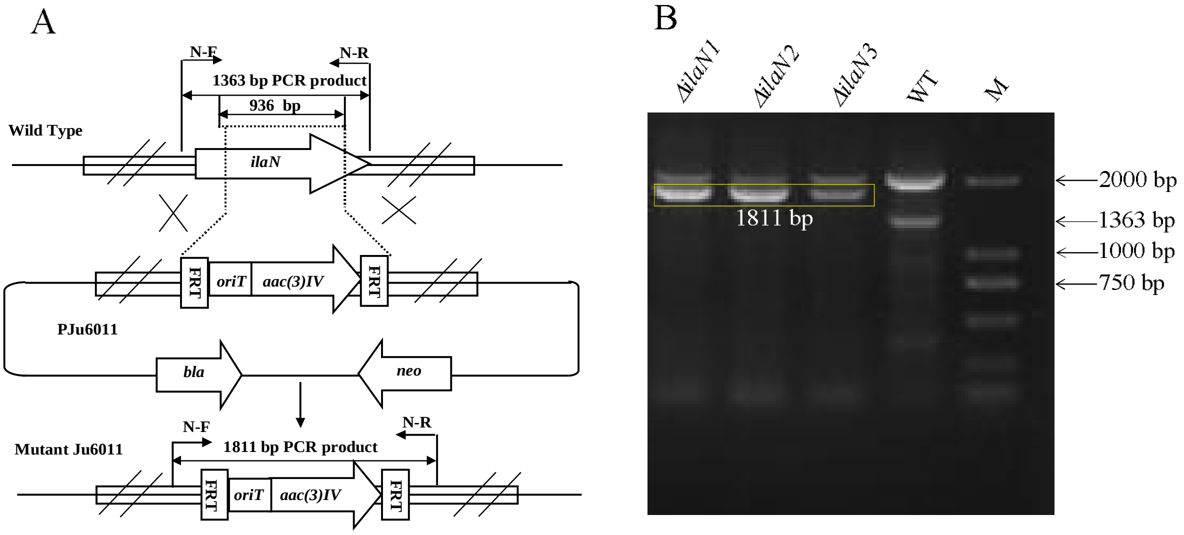

Supplementary Figure 13. Disruption of *ilaN* in wild-type *S. atratus* SCSIO ZH16 via PCR-targeting. (A) Schematic representation for disruption of *ilaN.* (B) PCR analyses of the wild-type strain and the *ilaN* double-cross mutant carried out using the primers listed in Supplementary Table 5. M: DNA molecular ladder; WT: using the genomic DNA of *S. atratus* SCSIO ZH16 as template; *ΔilaN1-3*: using the genomic DNA of *ilaN* mutant as template.

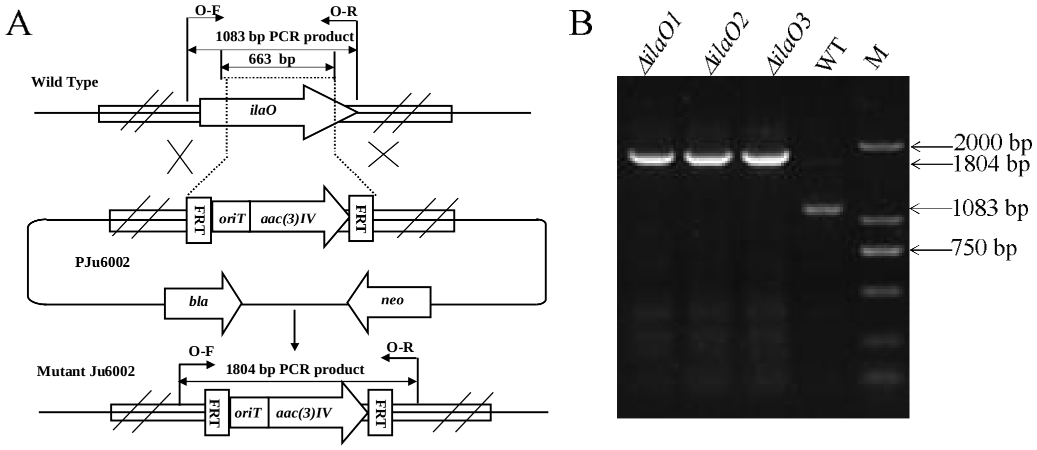

Supplementary Figure 14. Disruption of *ilaO* in wild-type *S. atratus* SCSIO ZH16 via PCR-targeting. (A) Schematic representation for disruption of *ilaO.* (B) PCR analyses of the wild-type strain and the *ilaO* double-cross mutant carried out using the primers listed in Supplementary Table 5. M: DNA molecular ladder; WT: using the genomic DNA of *S. atratus* SCSIO ZH16 as template; *ΔilaO1-3*: using the genomic DNA of *ilaO* mutant as template.

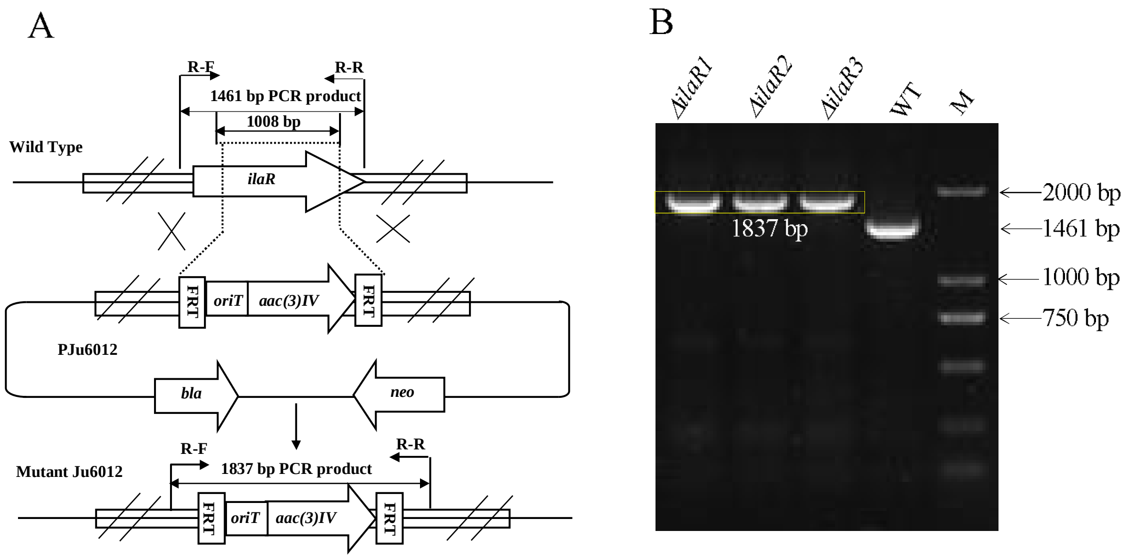

Supplementary Figure 15. Disruption of *ilaR* in wild-type *S. atratus* SCSIO ZH16 via PCR-targeting. (A) Schematic representation for disruption of *ilaR.* (B*)* PCR analyses of the wild-type strain and the *ilaR* double-cross mutant carried out using the primers listed in Supplementary Table 5. M: DNA molecular ladder; WT: using the genomic DNA of *S. atratus* SCSIO ZH16 as template; *ΔilaR1-3*: using the genomic DNA of *ilaR* mutant as template.

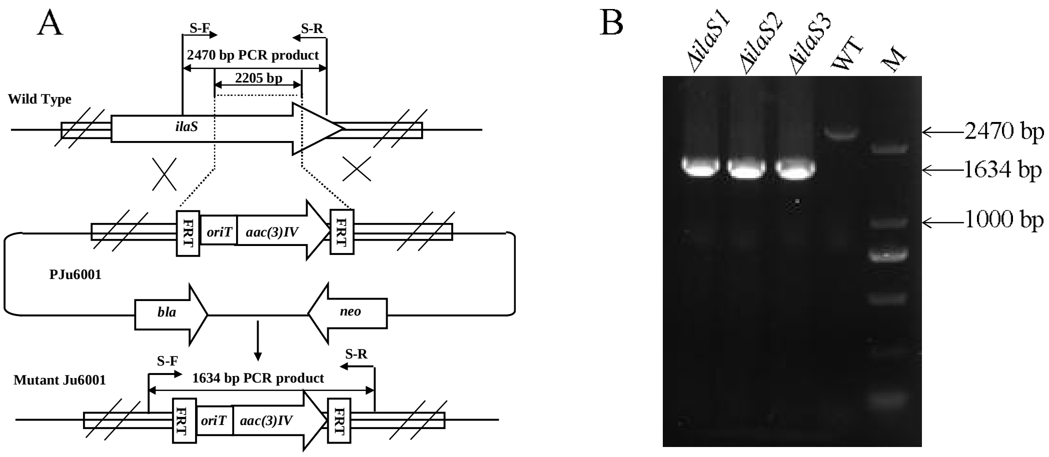

Supplementary Figure 16. Disruption of *ilaS* in wild-type *S. atratus* SCSIO ZH16 via PCR-targeting. (A) Schematic representation for disruption of *ilaS.* (B*)* PCR analyses of the wild-type strain and the *ilaS* double-cross mutant carried out using the primers listed in Supplementary Table 5. M: DNA molecular ladder; WT: using the genomic DNA of *S. atratus* SCSIO ZH16 as template; *ΔilaS1-3*: using the genomic DNA of *ilaS* mutant as template.

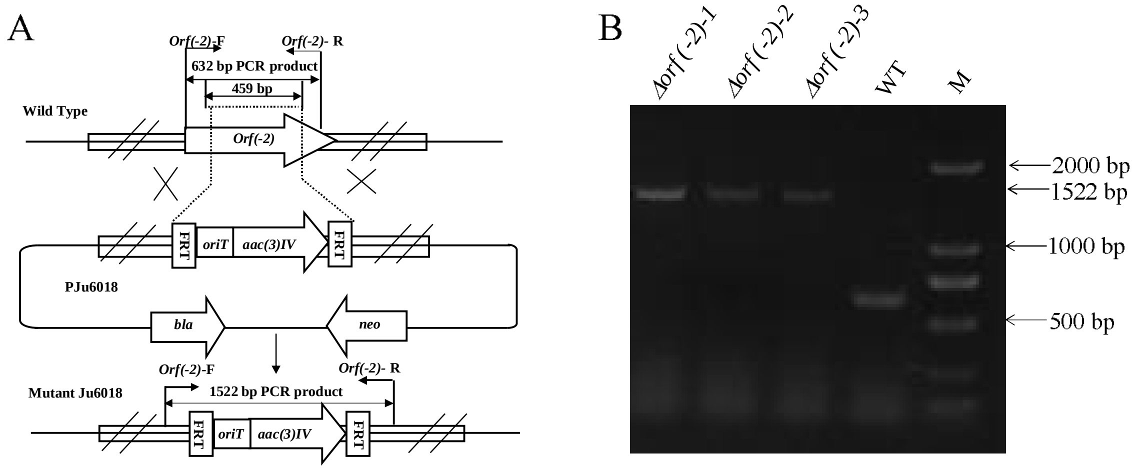

Supplementary Figure 17. Disruption of *orf(-2)* in wild-type *S. atratus* SCSIO ZH16 via PCR-targeting. (A) Schematic representation for disruption of *orf(-2).* (B*)* PCR analyses of the wild-type strain and the *orf(-2)* double-cross mutant carried out using the primers listed in Supplementary Table 5. M: DNA molecular ladder; WT: using the genomic DNA of *S. atratus* SCSIO ZH16 as template; *Δorf(-2)-1-3*: using the genomic DNA of *orf(-2)* mutant as template.

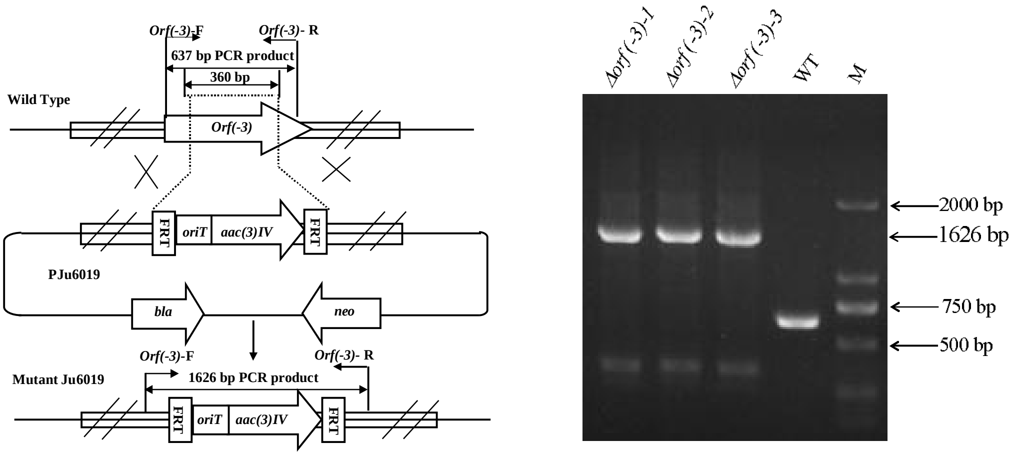

Supplementary Figure 18. Disruption of *orf(-3)* in wild-type *S. atratus* SCSIO ZH16 via PCR-targeting. (A) Schematic representation for disruption of *orf(-3).* (B*)* PCR analyses of the wild-type strain and the *orf(-3)* double-cross mutant carried out using the primers listed in Supplementary Table 5. M: DNA molecular ladder; WT: using the genomic DNA of *S. atratus* SCSIO ZH16 as template; *Δorf(-3)-1-3*: using the genomic DNA of *orf(-3)* mutant as template.

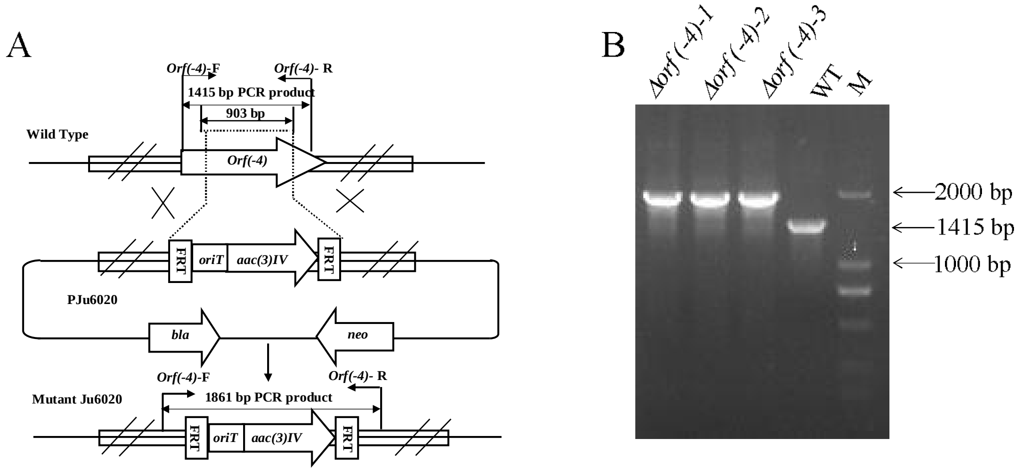

Supplementary Figure 19. Disruption of *orf(-4)* in wild-type *S. atratus* SCSIO ZH16 via PCR-targeting. (A) Schematic representation for disruption of *orf(-4).* (B*)* PCR analyses of the wild-type strain and the *orf(-4)* double-cross mutant carried out using the primers listed in Supplementary Table 5. M: DNA molecular ladder; WT: using the genomic DNA of *S. atratus* SCSIO ZH16 as template; *Δorf(-4)-1-3*: using the genomic DNA of *orf(-4)* mutant as template.

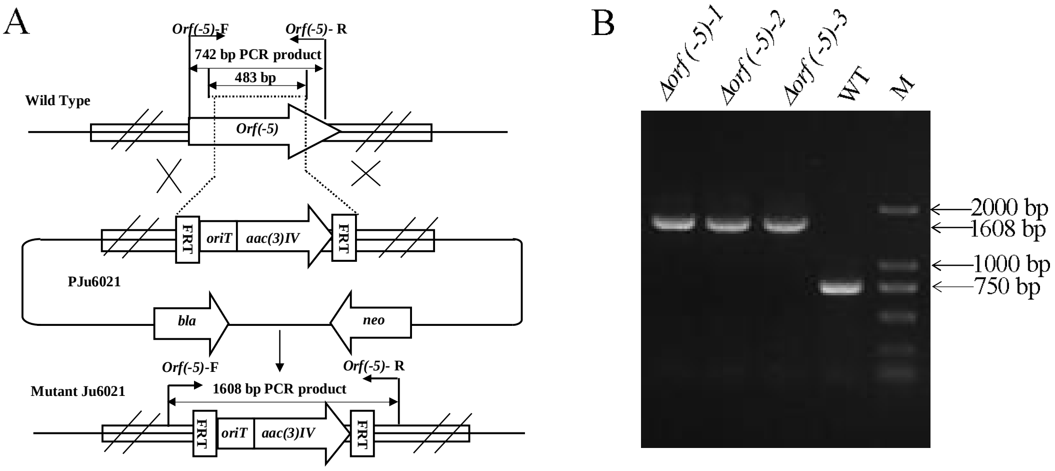

Supplementary Figure 20. Disruption of *orf(-5)* in wild-type *S. atratus* SCSIO ZH16 via PCR-targeting. (A) Schematic representation for disruption of *orf(-5).* (B*)* PCR analyses of the wild-type strain and the *orf(-5)* double-cross mutant carried out using the primers listed in Supplementary Table 5. M: DNA molecular ladder; WT: using the genomic DNA of *S. atratus* SCSIO ZH16 as template; *Δorf(-5)-1-3*: using the genomic DNA of *orf(-5)* mutant as template.

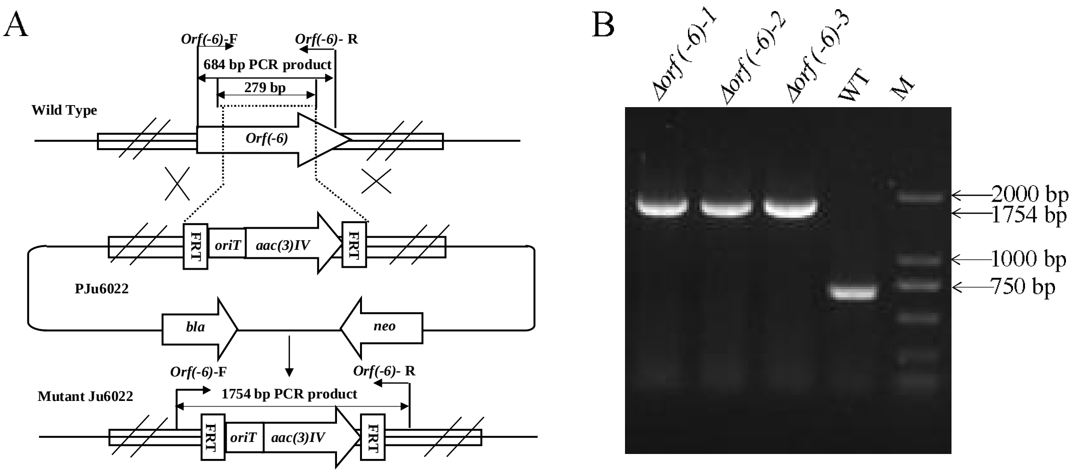

Supplementary Figure 21. Disruption of *orf(-6)* in wild-type *S. atratus* SCSIO ZH16 via PCR-targeting. (A) Schematic representation for disruption of *orf(-6).* (B*)* PCR analyses of the wild-type strain and the *orf(-6)* double-cross mutant carried out using the primers listed in Supplementary Table 5. M: DNA molecular ladder; WT: using the genomic DNA of *S. atratus* SCSIO ZH16 as template; *Δorf(-6)-1-3*: using the genomic DNA of *orf(-6)* mutant as template.

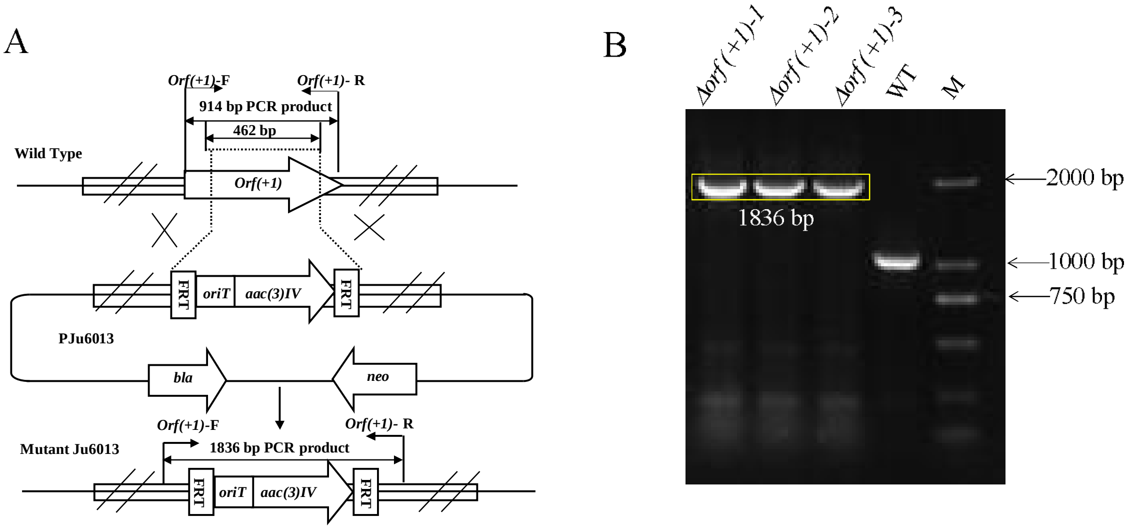

Supplementary Figure 22. Disruption of *orf(+1)* in wild-type *S. atratus* SCSIO ZH16 via PCR-targeting. (A) Schematic representation for disruption of *orf(+1).* (B*)* PCR analyses of the wild-type strain and the *orf(+1)* double-cross mutant carried out using the primers listed in Supplementary Table 5. M: DNA molecular ladder; WT: using the genomic DNA of *S. atratus* SCSIO ZH16 as template; *Δorf(+1)-1-3*: using the genomic DNA of *orf(+1)* mutant as template.

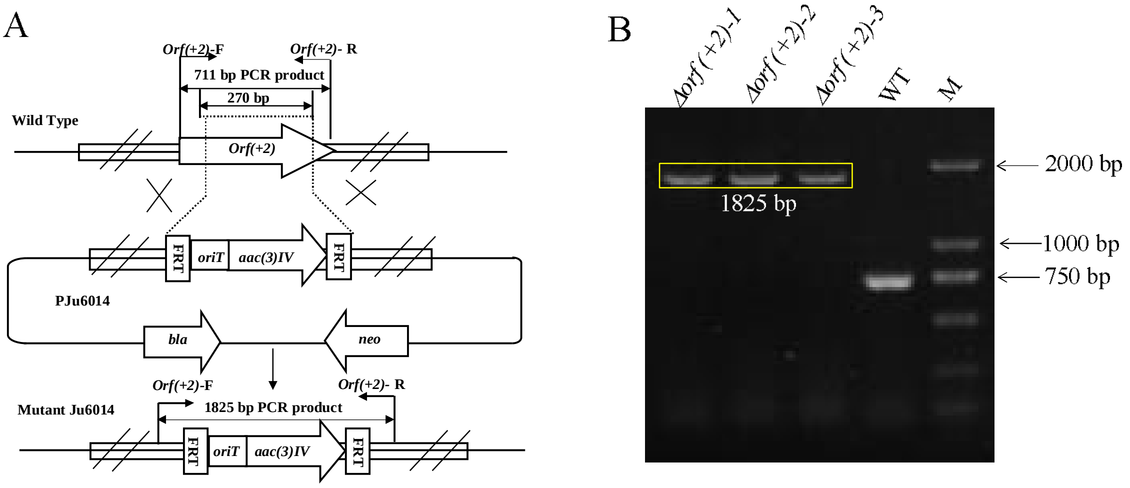

Supplementary Figure 23. Disruption of *orf(+2)* in wild-type *S. atratus* SCSIO ZH16 via PCR-targeting. (A) Schematic representation for disruption of *orf(+2).* (B*)* PCR analyses of the wild-type strain and the *orf(+2)* double-cross mutant carried out using the primers listed in Supplementary Table 5. M: DNA molecular ladder; WT: using the genomic DNA of *S. atratus* SCSIO ZH16 as template; *Δorf(+2)-1-3*: using the genomic DNA of *orf(+2)* mutant as template.

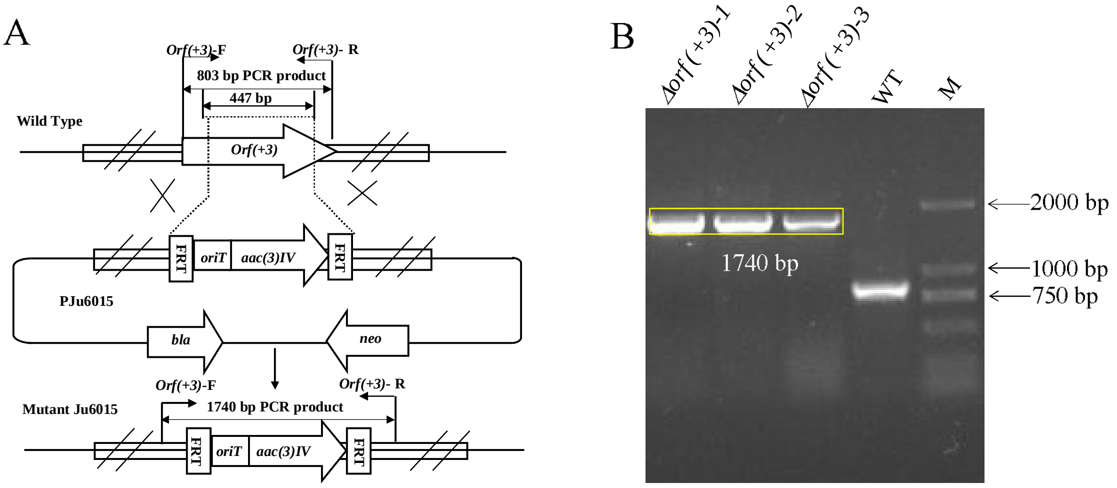

Supplementary Figure 24. Disruption of *orf(+3)* in wild-type *S. atratus* SCSIO ZH16 via PCR-targeting. (A) Schematic representation for disruption of *orf(+3).* (B*)* PCR analyses of the wild-type strain and the *orf(+3)* double-cross mutant carried out using the primers listed in Supplementary Table 5. M: DNA molecular ladder; WT: using the genomic DNA of *S. atratus* SCSIO ZH16 as template; *Δorf(+3)-1-3*: using the genomic DNA of *orf(+3)* mutant as template.

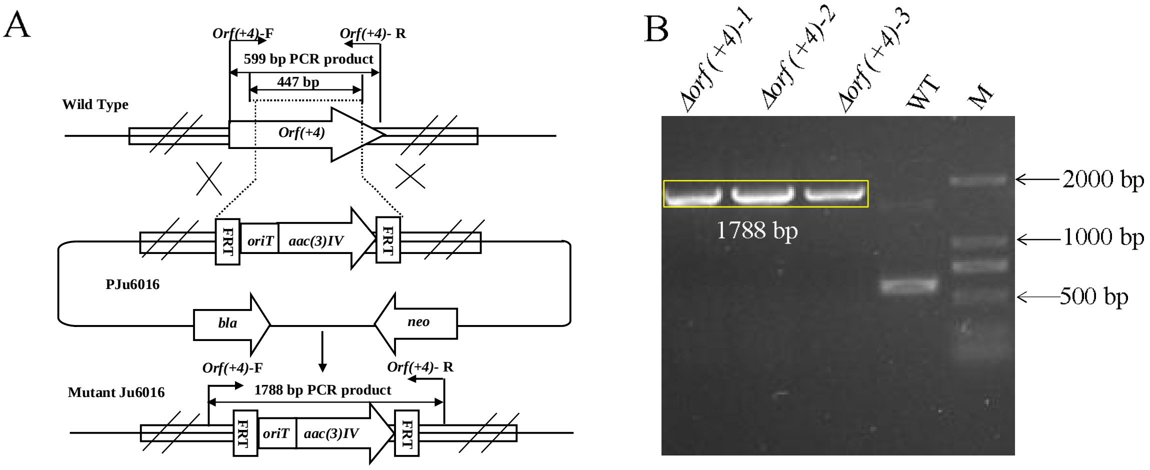

Supplementary Figure 25. Disruption of *orf(+4)* in wild-type *S. atratus* SCSIO ZH16 via PCR-targeting. (A) Schematic representation for disruption of *orf(+4).* (B*)* PCR analyses of the wild-type strain and the *orf(+4)* double-cross mutant carried out using the primers listed in Supplementary Table 5. M: DNA molecular ladder; WT: using the genomic DNA of *S. atratus* SCSIO ZH16 as template; *Δorf(+4)-1-3*: using the genomic DNA of *orf(+4)* mutant as template.

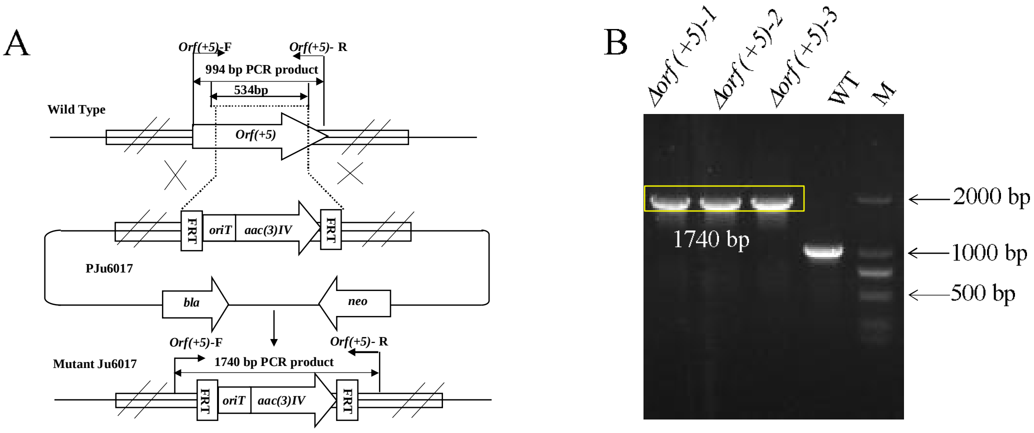

Supplementary Figure 26. Disruption of *orf(+5)* in wild-type *S. atratus* SCSIO ZH16 via PCR-targeting. (A) Schematic representation for disruption of *orf(+5).* (B*)* PCR analyses of the wild-type strain and the *orf(+5)* double-cross mutant carried out using the primers listed in Supplementary Table 5. M: DNA molecular ladder; WT: using the genomic DNA of *S. atratus* SCSIO ZH16 as template; *Δorf(+5)-1-3*: using the genomic DNA of *orf(+5)* mutant as template.

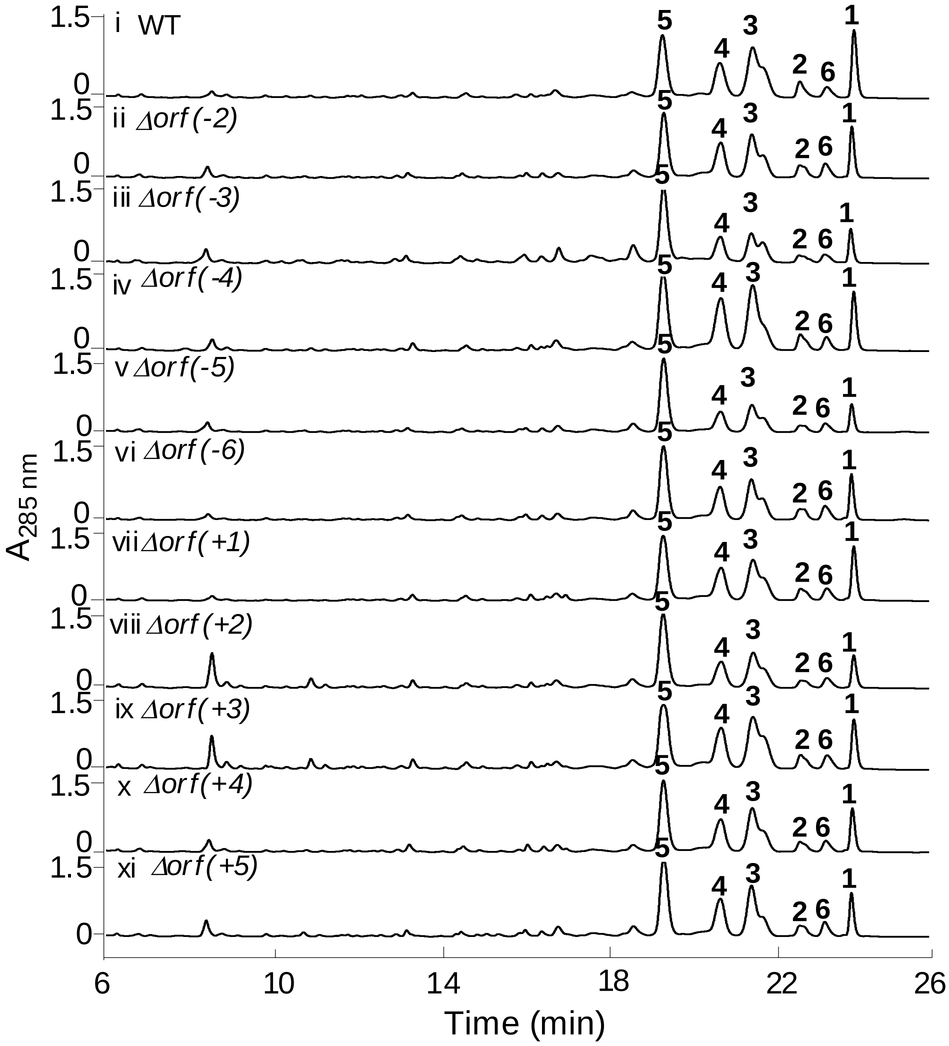

Supplementary Figure 27. HPLC analyses of fermentation brothes of *Δorf(-2)* to *Δorf(-6)*, and *Δorf(+1) to Δorf(+5)*. (i) ilamycin wild-type producer *S. atratus* SCSIO ZH16; (ii) *Δorf(-2)* mutant strain; (iii) *Δorf(-3)* mutant strain; (iv) *Δorf(-4)* mutant strain; (v) *Δorf(-5)*mutant strain; (vi) *Δorf(-6)* mutant strain; (vii) *Δorf(+1)* mutant strain; (viii) *Δorf(+2)* mutant strain; (ix) *Δorf(+3)* mutant strain; (x) *Δorf(+4)* mutant strain; (xi) *Δorf(+5)* mutant strain.

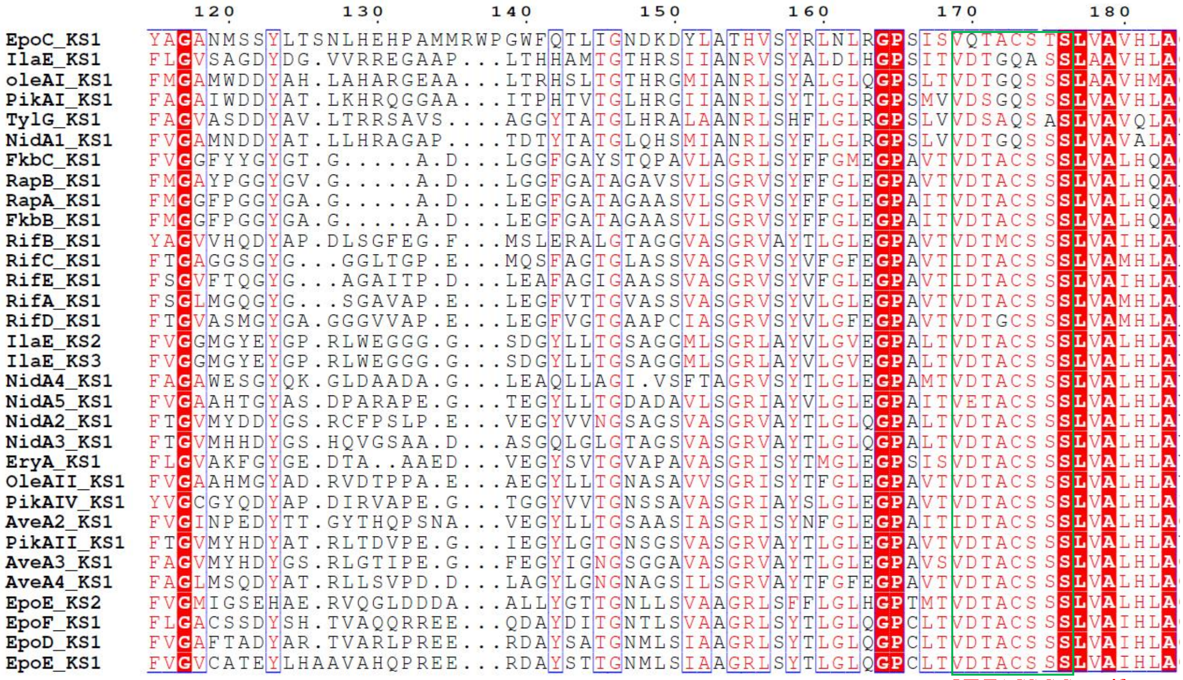

Supplementary Figure 28. Sequence alignments of IlaE_KS$_{1\text{-}3}$ with other KS domains in different antibiotics biosynthesis pathways. EpoC-F_KSs, epothilone, AF217189, *Sorangian cellulosin*; OleAI/AII_KSs, oleandomycin, AF220951, *Streptomyces antibiotics*; PikAI/AIV_KSs, pikromycin, AF079138, *Streptomyces venezuelae*; TylG_KSs, tylosin, U78289, *Streptomyces fradiae*; NidA1-A5_KSs, niddamycin, AF016585, *Streptomuces caelestis*; FkbC_KSs, FK520, AF235504, *Streptomyces hygroscopicus sub. sp. ascomyceticus*; RapA/B_KSs, rapamycin, X86780, *Streptomyces rapamycincius*; RifA-E_KSs, rifamycin, AF040570, *Amycolatopsis mediterranei* S699; EryA_KSs, erythromycin, X63569, *Saccharopolyspora erythraea* NRRL 2338; KS numbers refer to the module in which the domain appears. VDTACSSS motif is shown in green box and highlighted in red letters at the bottom of the alignment sequences.

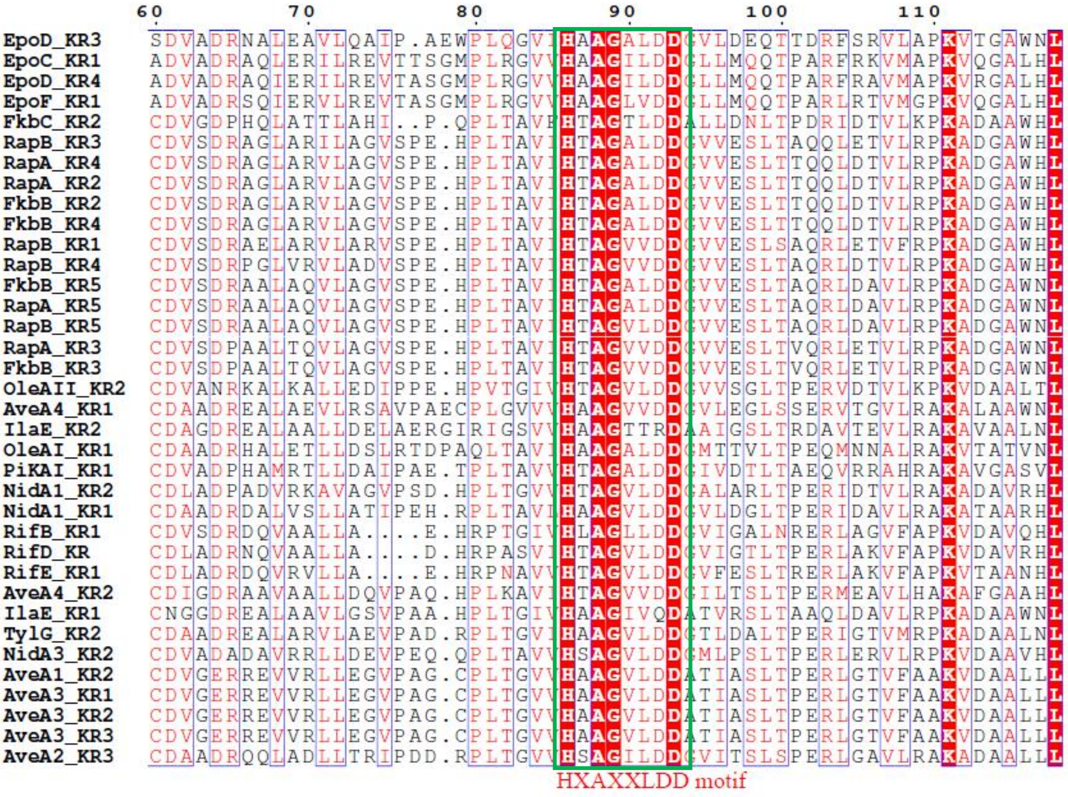

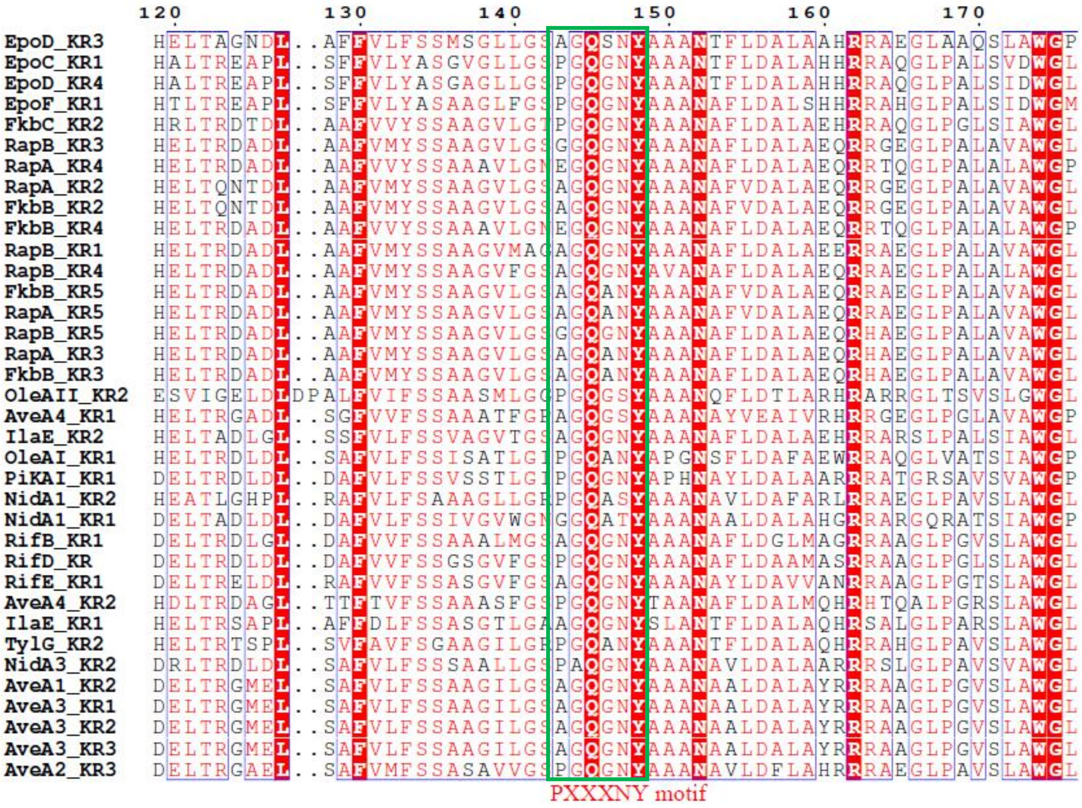

Supplementary Figure 29. Sequence alignments of IlaE_KR$_{2\text{-}3}$ with other KR domains in different antibiotic biosynthesis pathways. The KR domains in different antibiotic biosynthesis pathways, sequence accession numbers and the respective strains are as follows: EpoD_KRs,

epothilone, AF217189, *Sorangian cellulosin*; RapA/B_KRs, rapamycin, X86780, *Streptomyces rapamycincius*; FkbA/B_KRs, FK520, AF235504, *Streptomyces hygroscopicus sub. sp. ascomyceticus*; OleAI/AII_KRs, oleandomycin, AF220951, *Streptomyces antibiotics*; PikAI-KR1, pikromycin, AF079138, *Streptomyces venezuelae*; NidA1/A3_Rs, niddamycin, AF016585, *Streptomuces caelestis*; RifB-D_KRs, rifamycin, AF040570, *Amycolatopsis mediterranei* S699; AveA1-A3_KRs, avermectin, AB032367, *Streptomyces avermitilis*; TylG_KRs, tylosin, U78289, *Streptomyces fradiae*. KR numbers refer to the module in which the domain appears. HXAXXLDD motif and PXXXNY motifs are shown in green box and highlighted in red letters at the bottom of the alignment sequences.

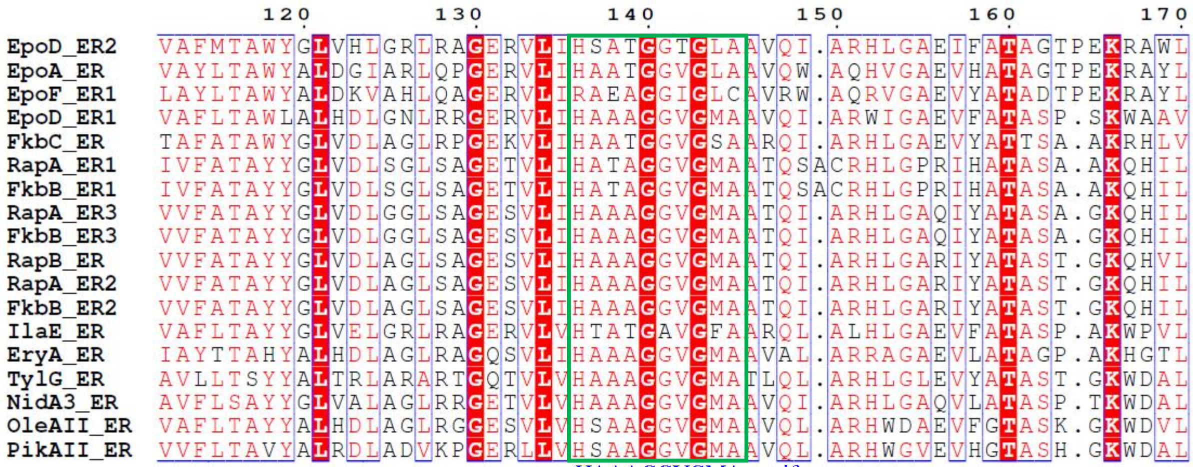

Supplementary Figure 30. Sequence alignments of IlaE_ER domain with other ER domains in different antibiotics biosynthesis pathways. The ER domains in different antibiotics biosynthesis pathways, sequence accession numbers and respective strains are as follows: EpoA/D/F_ERs, epothilone, AF217189, *Sorangian cellulosin*; FkbB_ERs, FK520, AF235504, *Streptomyces hygroscopicus sub. sp. ascomyceticus*; RapA/B_ERs, rapamycin, X86780, *Streptyomyces rapamycincius*; EryA_ER, erythromycin, X63569, *Saccharopolyspora erythraea* NRRL 2338; TylG-ER, tylosin, U78289, *Streptomyces fradiae*; NidA3_ER, niddamycin, AF016585, *Streptomuces caelestis*; OleAII_ERs, oleandomycin, AF220951, *Streptomyces antibiotics*; PikAII_ERs, pikromycin, AF079138, *Streptomyces Venezuelae*. ER numbers refer to the module in which the domain appears. The conserved HAAAGGVGMA motif of ER domain is shown in green box and highlighted in blue letters at the bottom of the alignment sequences.

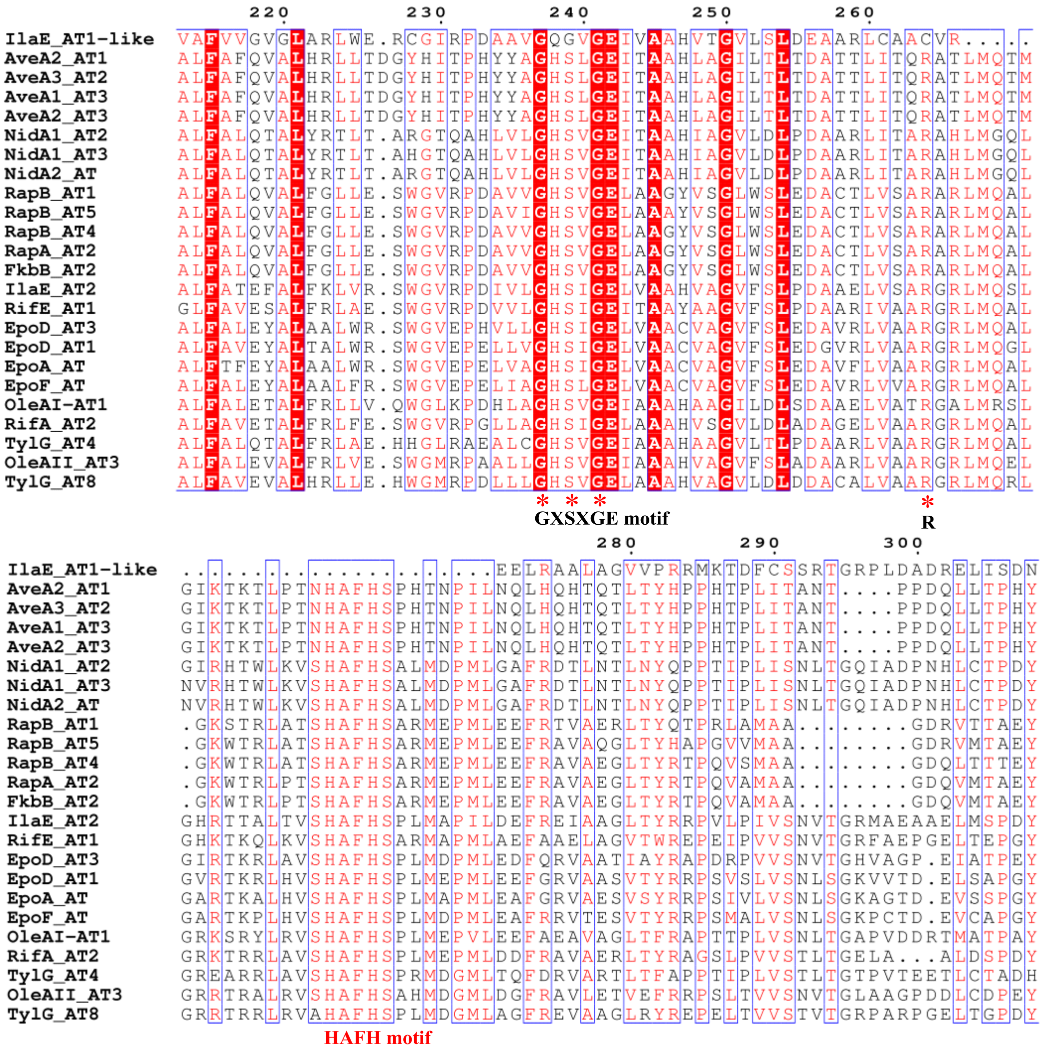

Supplementary Figure 31. Sequence alignments of IlaE_AT$_1$-like and IlaE_AT$_2$ domain with other AT domains in different antibiotics biosynthesis pathways. The AT domains in different antibiotics biosynthesis pathways, sequence accession numbers and respective strains are as follows: AveA1/A2_ATs, avermectin, AB032367, *Streptomyces avermitilis*; NidA1_ATs, niddamycin, AF016585, *Streptomuces caelestis*; RapA/B_ATs, rapamycin, X86780, *Streptomyces rapamycincius*; FkbA/B_ATs, FK520, AF235504, *Streptomyces hygroscopicus sub. sp. ascomyceticus*; EpoA/D/F_ATs, epothilone, AF217189, *Sorangian cellulosin*; OleAI_ATs, oleandomycin, AF220951, *Streptomyces antibiotics*; RifA_ATs, rifamycin, AF040570, *Amycolatopsis mediterranei* S699; TylG_ATs, tylosin, U78289, *Streptomyces fradiae*. AT numbers refer to the module in which the domain appears. The active site residues (G, S, G) in GXSXGE motif and R were labelled by red asterisk and highlighted in black letters; HAFH motifs specific for malnoyl-CoA are highlighted in red letters at the bottom of the alignment sequences.

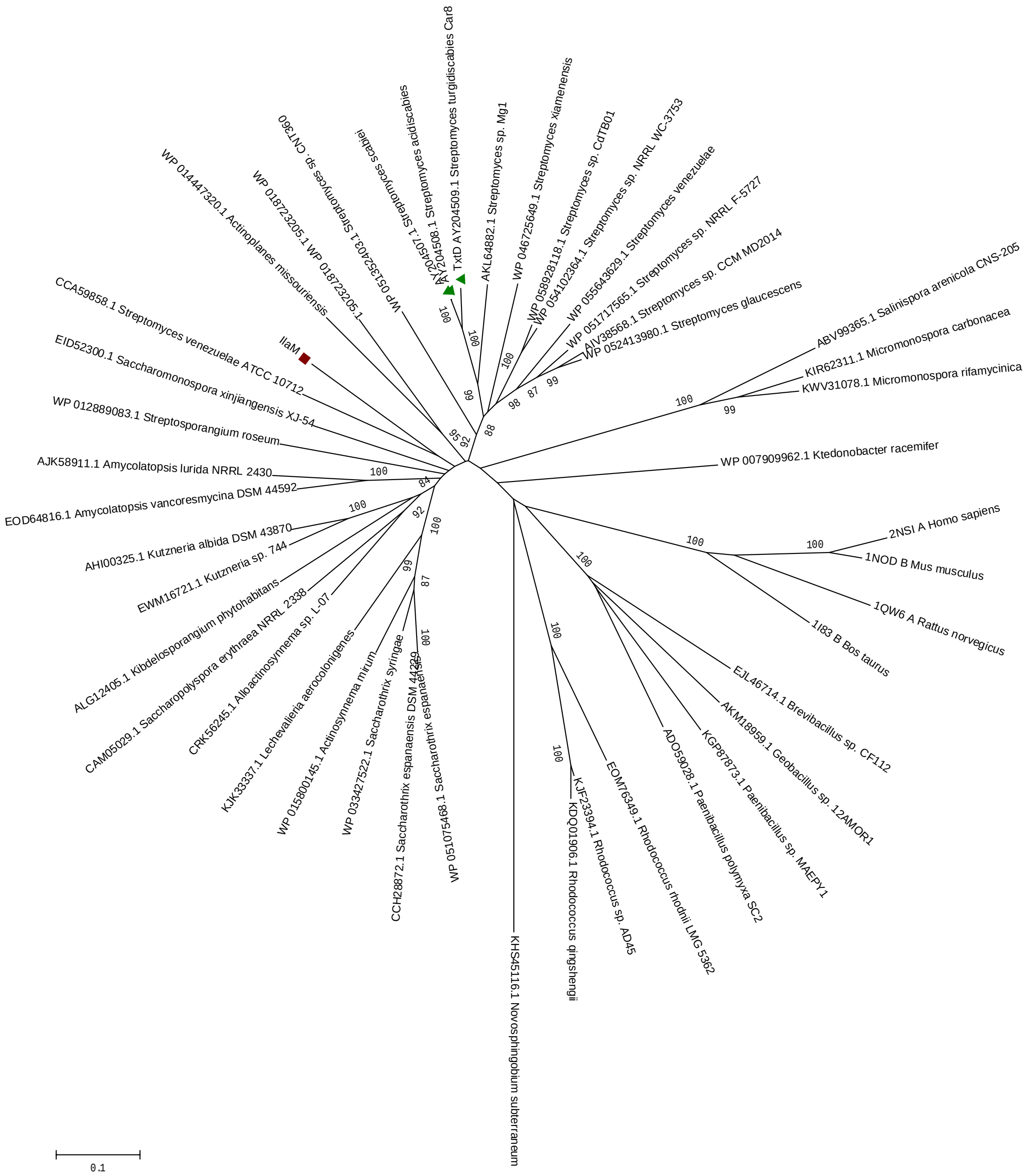

Supplementary Figure 32. Phylogenetic tree of IlaM with other nitric oxide synthase homologues. The amino acid sequences were aligned using ClustalW and the phylogenetic tree was generated using Molecular Evolutionary Genetics Analysis (MEGA) 6.0. IlaM is indicated in purplish red square.

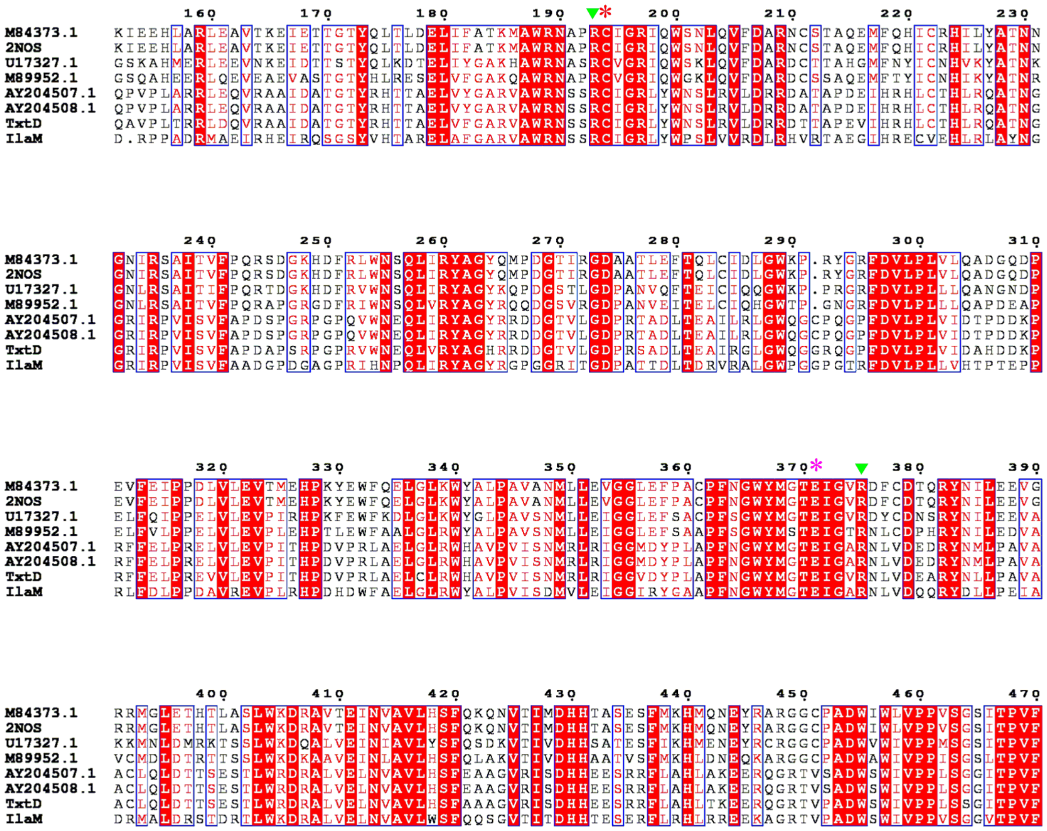

Supplementary Figure 33. Sequence alignments of IlaM with other nitric oxide synthase homologous. The sequence accession numbers and respective source are as follows: NCBI gi number: M84373.1 (iNOSoxy, murine), NCBI gi number: M89952.1 (eNOSoxy, bovine), NCBI gi number: U17327.1 (nNOSoxy, human), PDB number 2NOS (iNOSoxy, house mouse), NCBI gi number: AY204507.1 (NOS, *S. scabiei*), NCBI gi number: AY204508.1 (NOS, *S. acidiscabies*), and NCBI gi number: AY204509.1 (TxtD) (NOS, *S. turgidiscabies* Car8). The conserved proximal heme ligand C194, Arg-binding residue E371, and the H_4B binding sites R193 and R375 are indicated by red star, pink star and fluorescent green triangle, respectively.

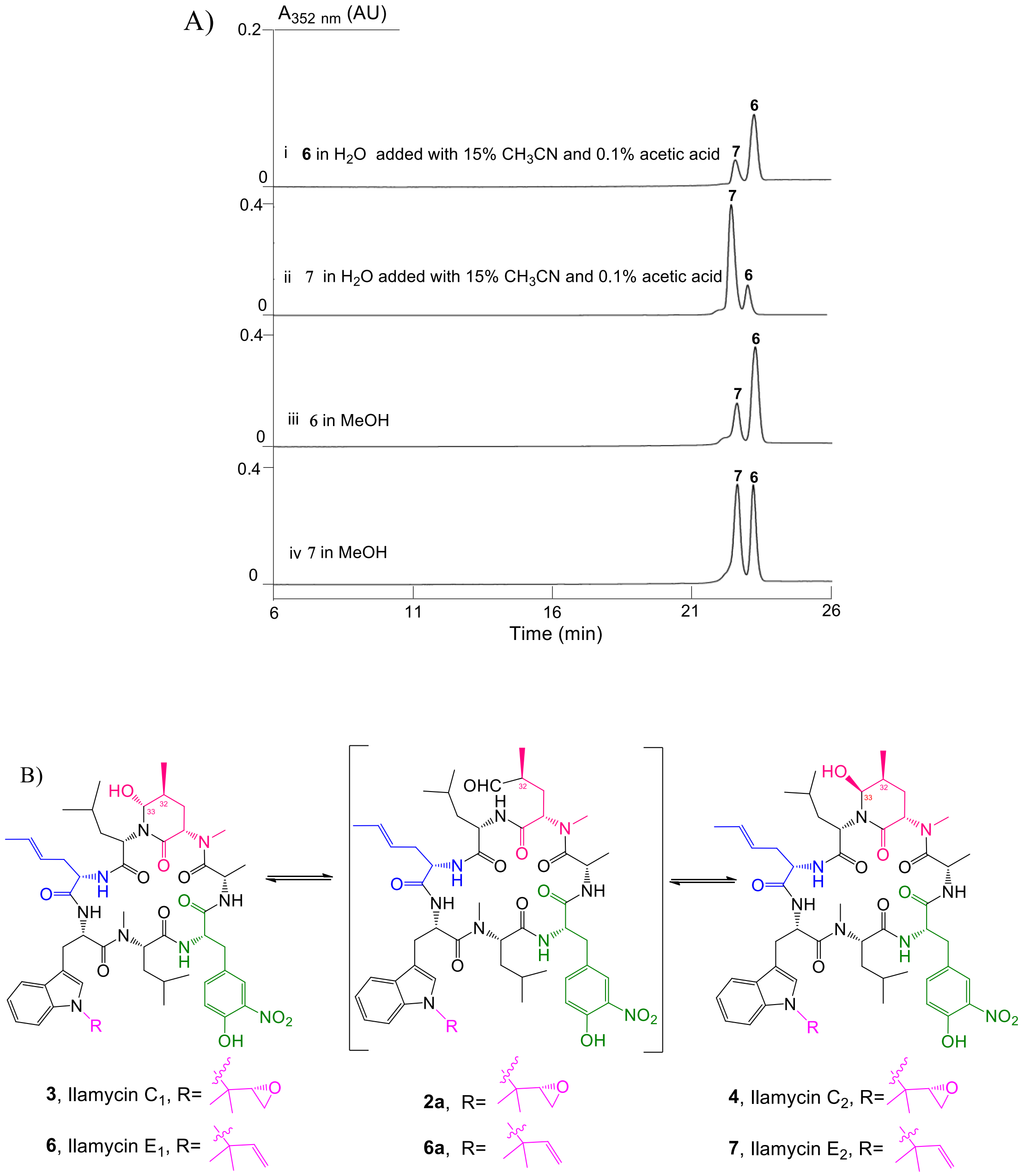

Supplementary Figure 34. The interconversion of compound **6** and **7** and the putative mechanisms for interconversion of two pairs of compounds **3**/**4** and **6**/**7**. A) The HPLC analysis of purified 6 and purified 7 dissolved in 15% CH_3CN-H_2O containing 0.1% acetic acid or in MeOH for 1 day at room temperature. B) Putative mechanisms for interconversion of ilamycins C_1 (**3**) and C_2 (**4**), and ilamycins E_1 (**6**) and E_2 (**7**).

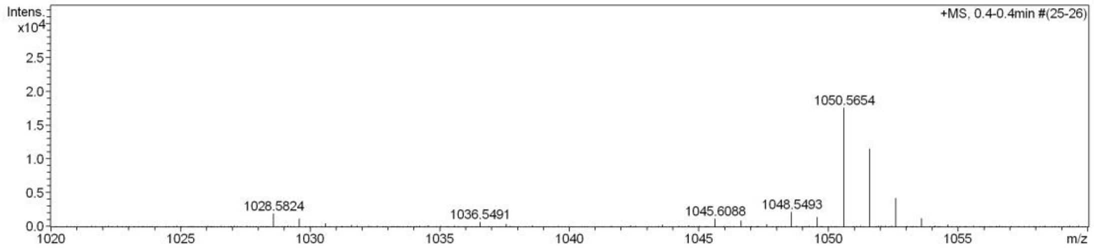

Supplementary Figure 35. (+) HRESIMS spectrum of ilamycin B_2 (**2**).

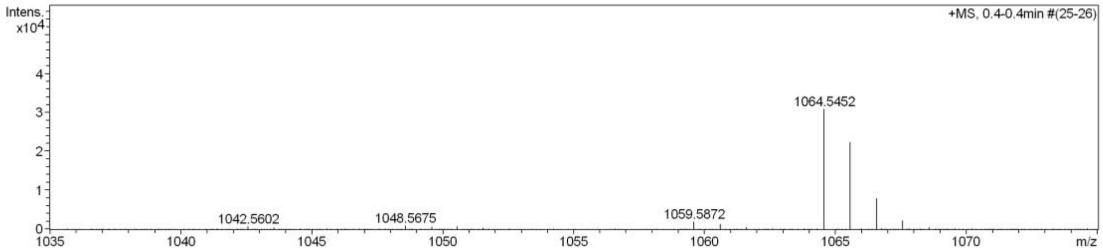

Supplementary Figure 36. (+) HRESIMS spectrum of ilamycin C_2 (**4**).

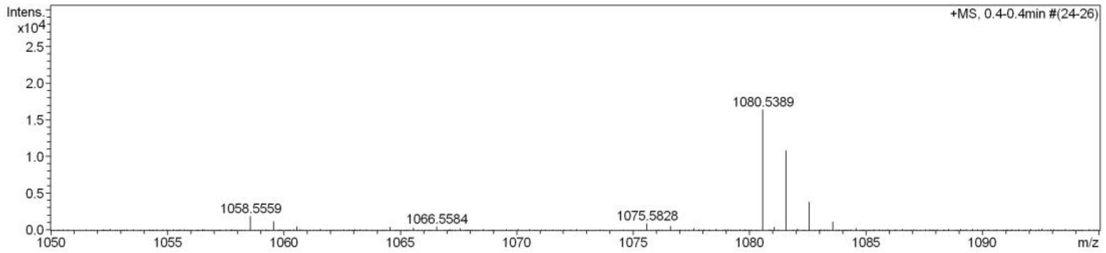

Supplementary Figure 37. (+) HRESIMS spectrum of ilamycin D (**5**).

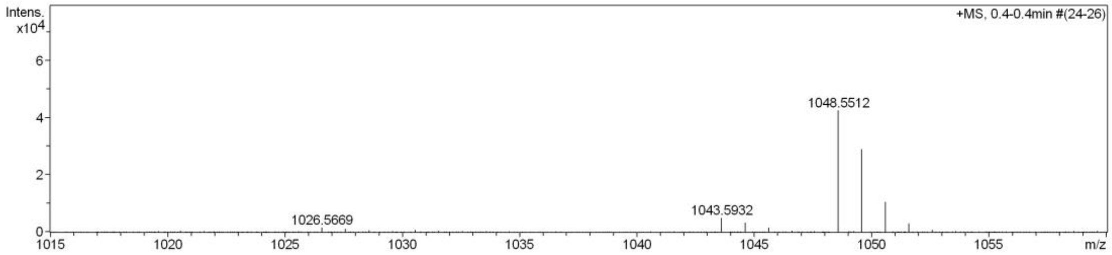

Supplementary Figure 38. (+) HRESIMS spectrum of ilamycin E_1 (**6**).

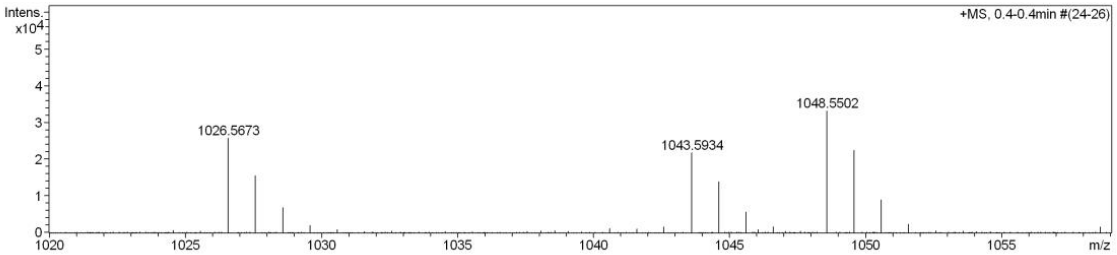

Supplementary Figure 39. (+) HRESIMS spectrum of ilamycin E_2 (**7**).

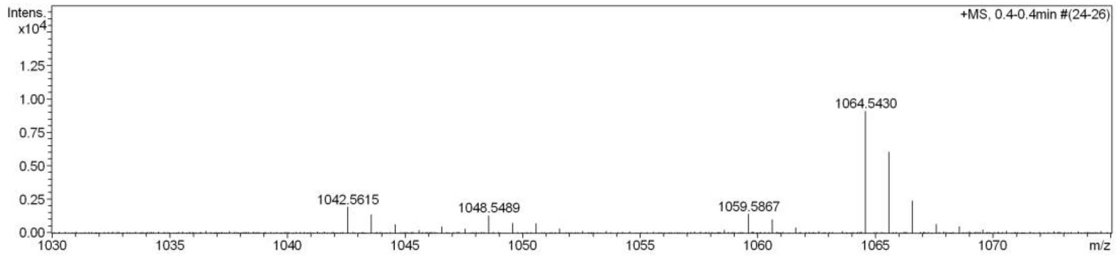

Supplementary Figure 40. (+) HRESIMS spectrum of ilamycin F (**8**).

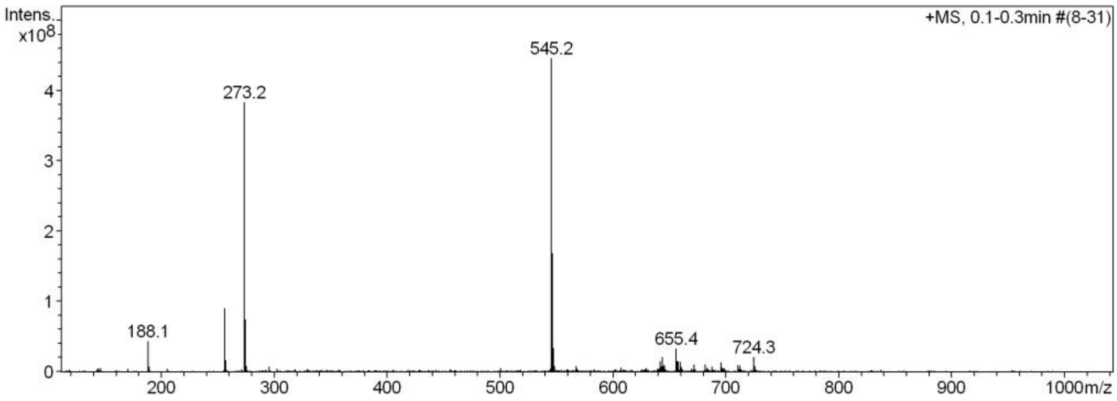

Supplementary Figure 41. (+) ESIMS spectrum of prenyl-tryptophan (**9**).

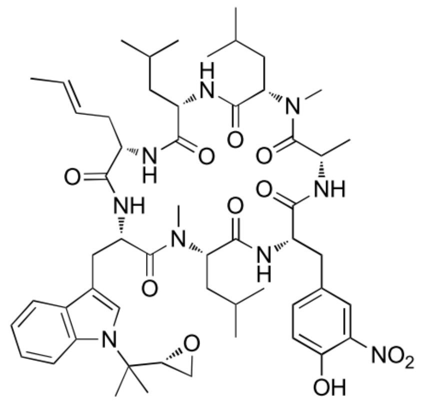

ilamycin B_2 (**2**)

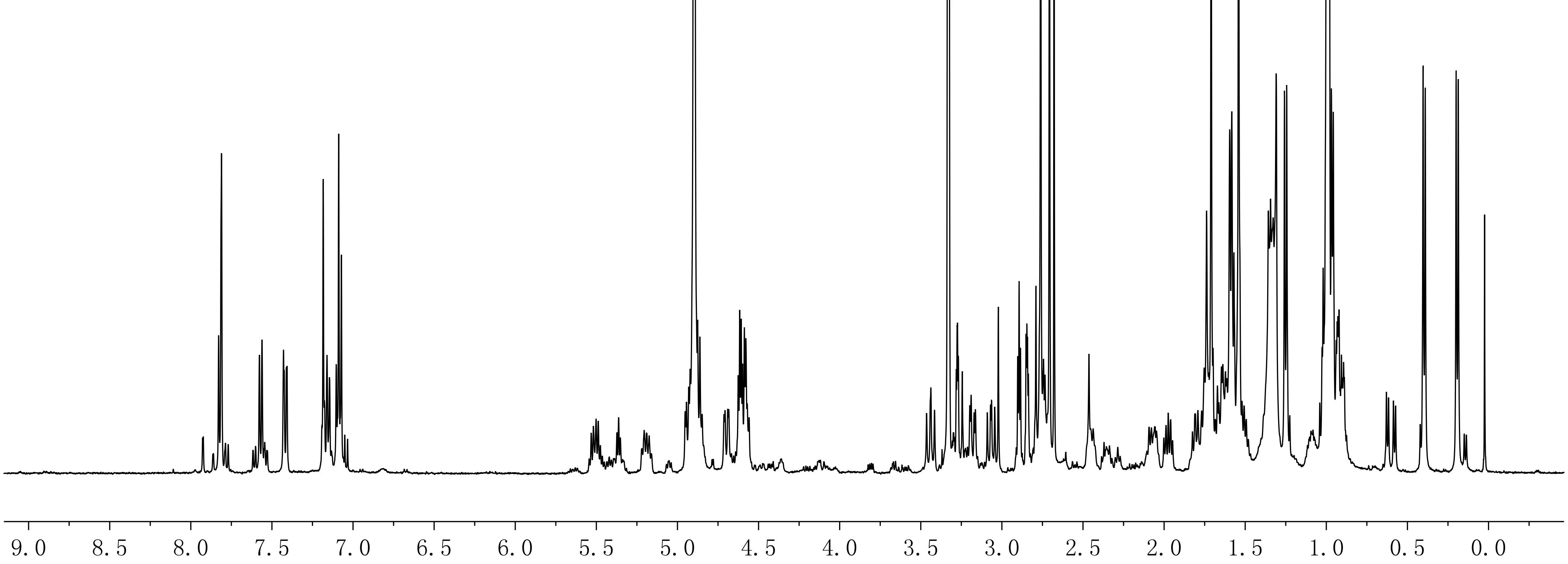

Supplementary Figure 42. ^{1}H NMR spectrum of ilamycin B_2 (**2**) in CD_3OD at 500 HMz.

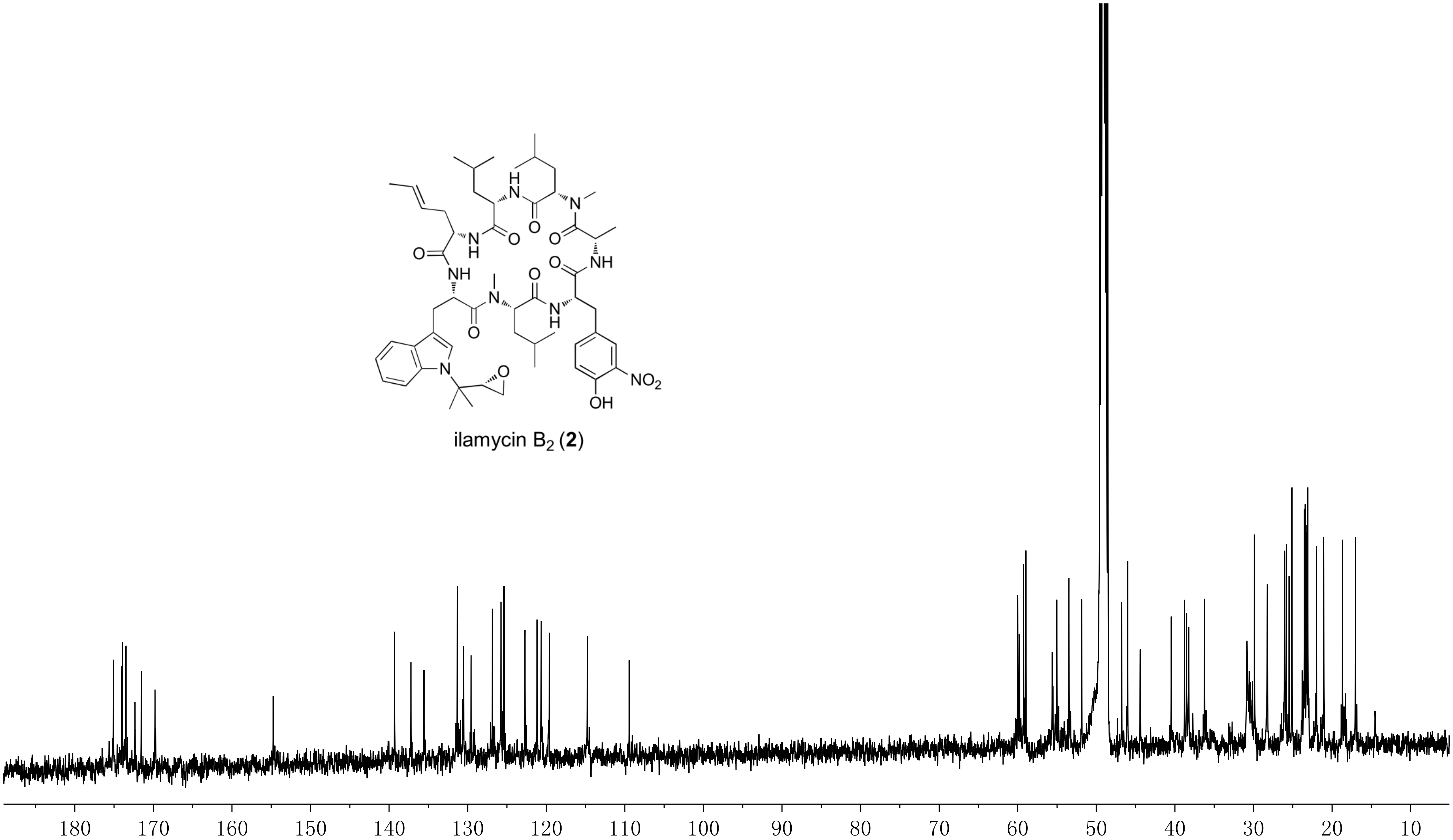

Supplementary Figure 43. ^{13}C NMR spectrum of ilamycin B$_2$ (**2**) in CD_3OD at 125 MHz.

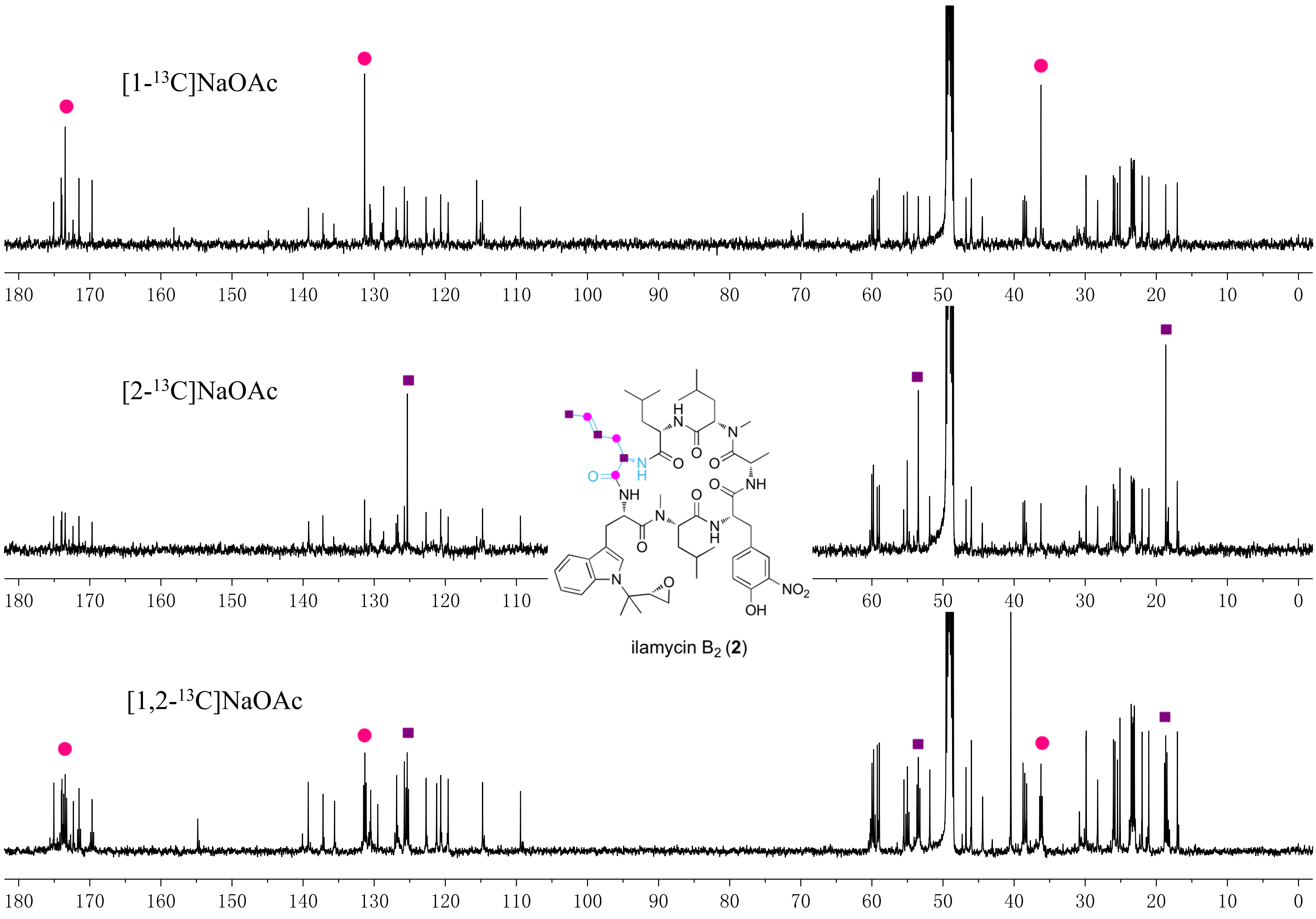

Supplementary Figure 44. ^{13}C NMR spectra of the ^{13}C-labeled ilamycin B_2 (**2**) in CD_3OD at 125 MHz.

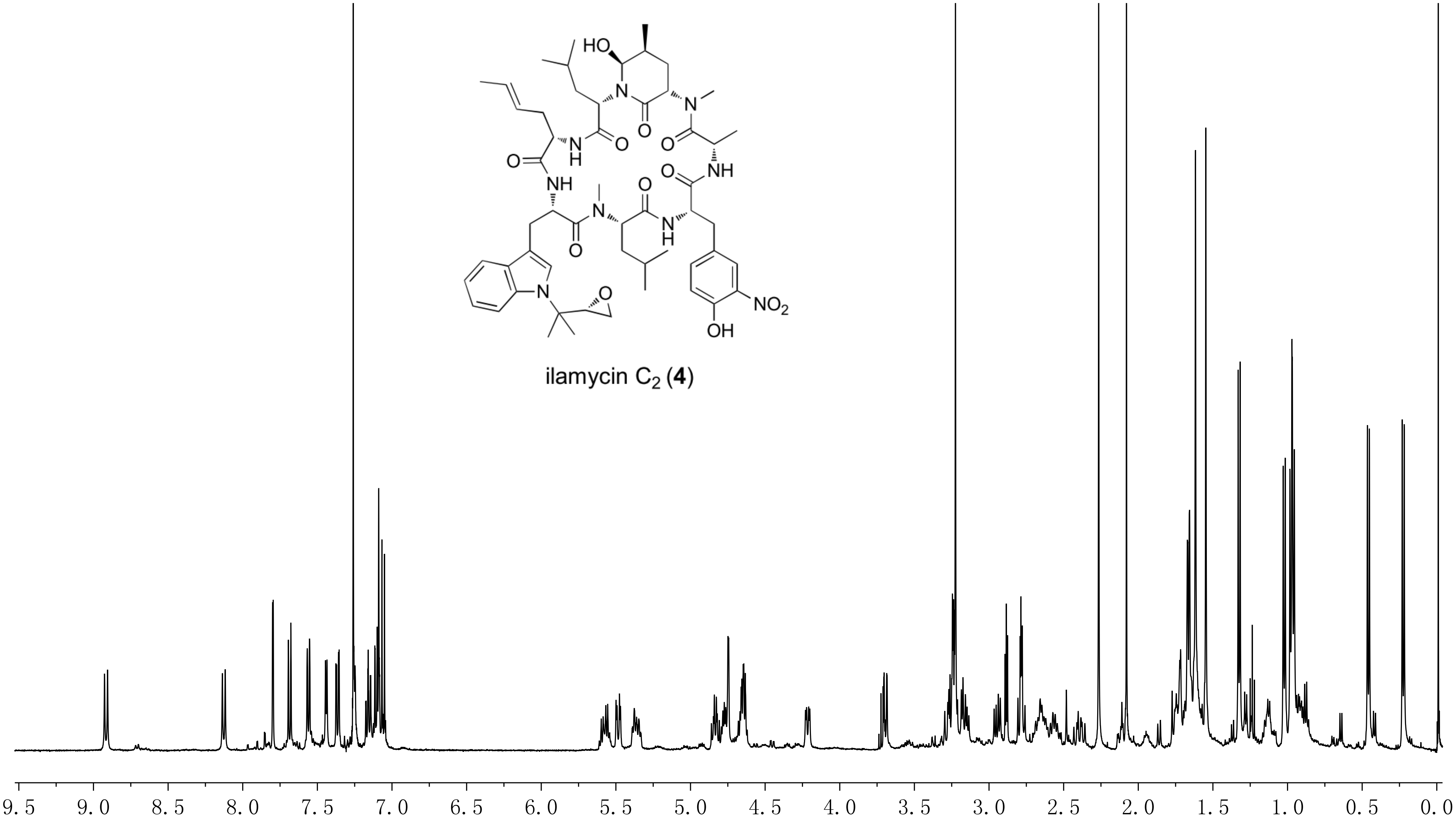

Supplementary Figure 45. ^{1}H NMR spectrum of ilamycin C_2 (**4**) in $CDCl_3$ at 500 MHZ.

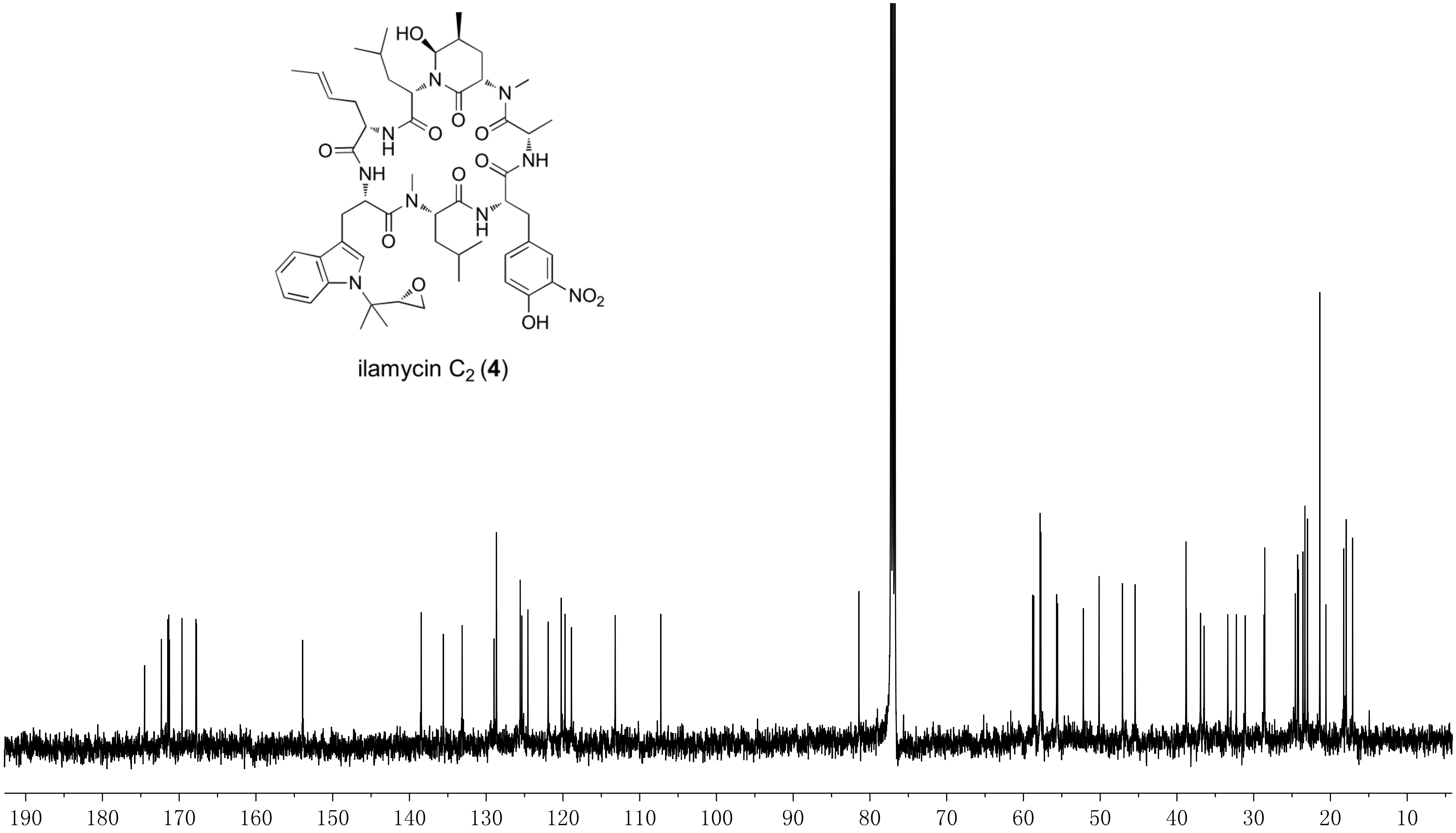

Supplementary Figure 46. ^{13}C NMR spectrum of ilamycin C_2 (**4**) in $CDCl_3$ at 125 MHz.

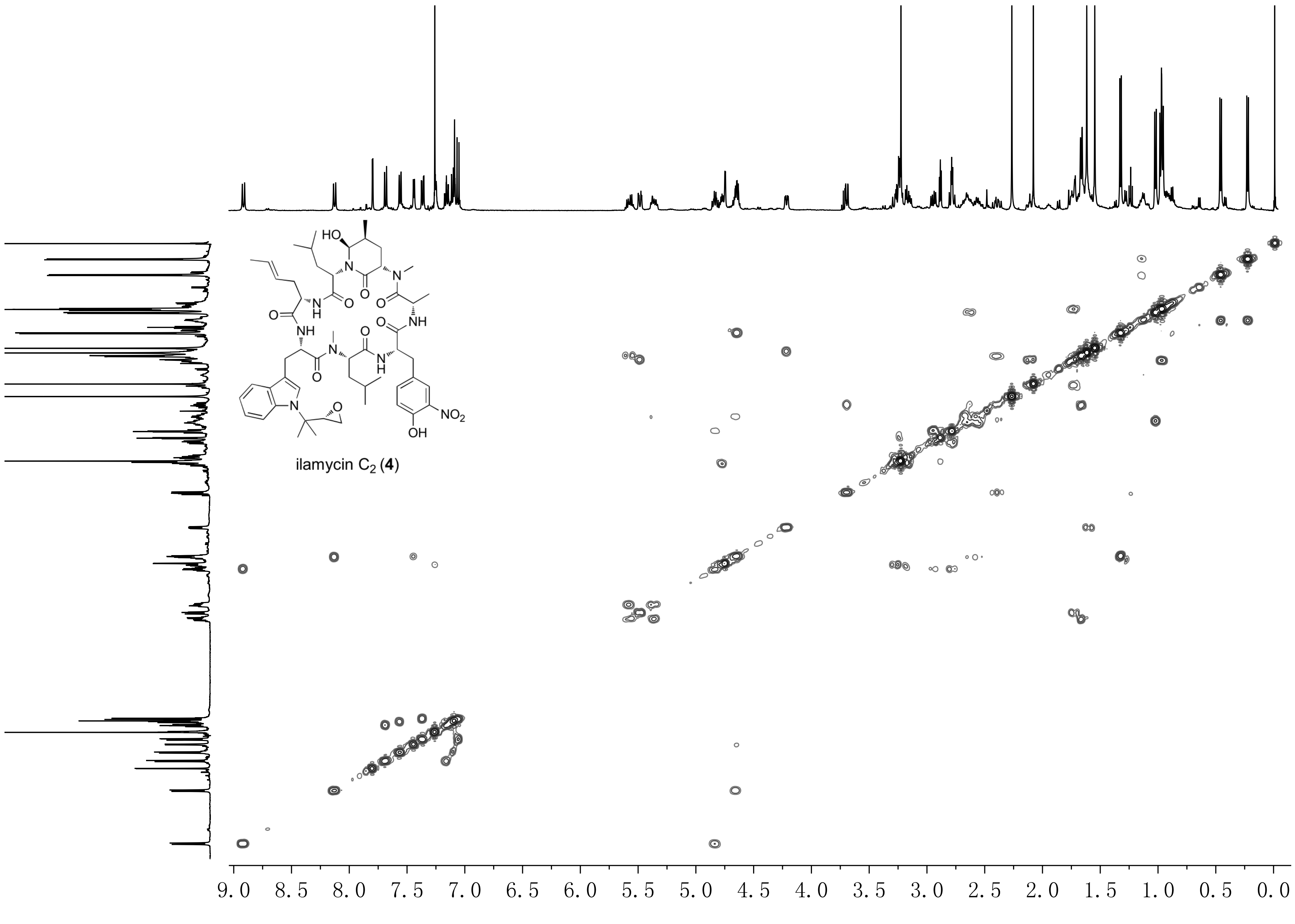

Supplementary Figure 47. COSY spectrum of ilamycin C_2 (**4**) in $CDCl_3$.

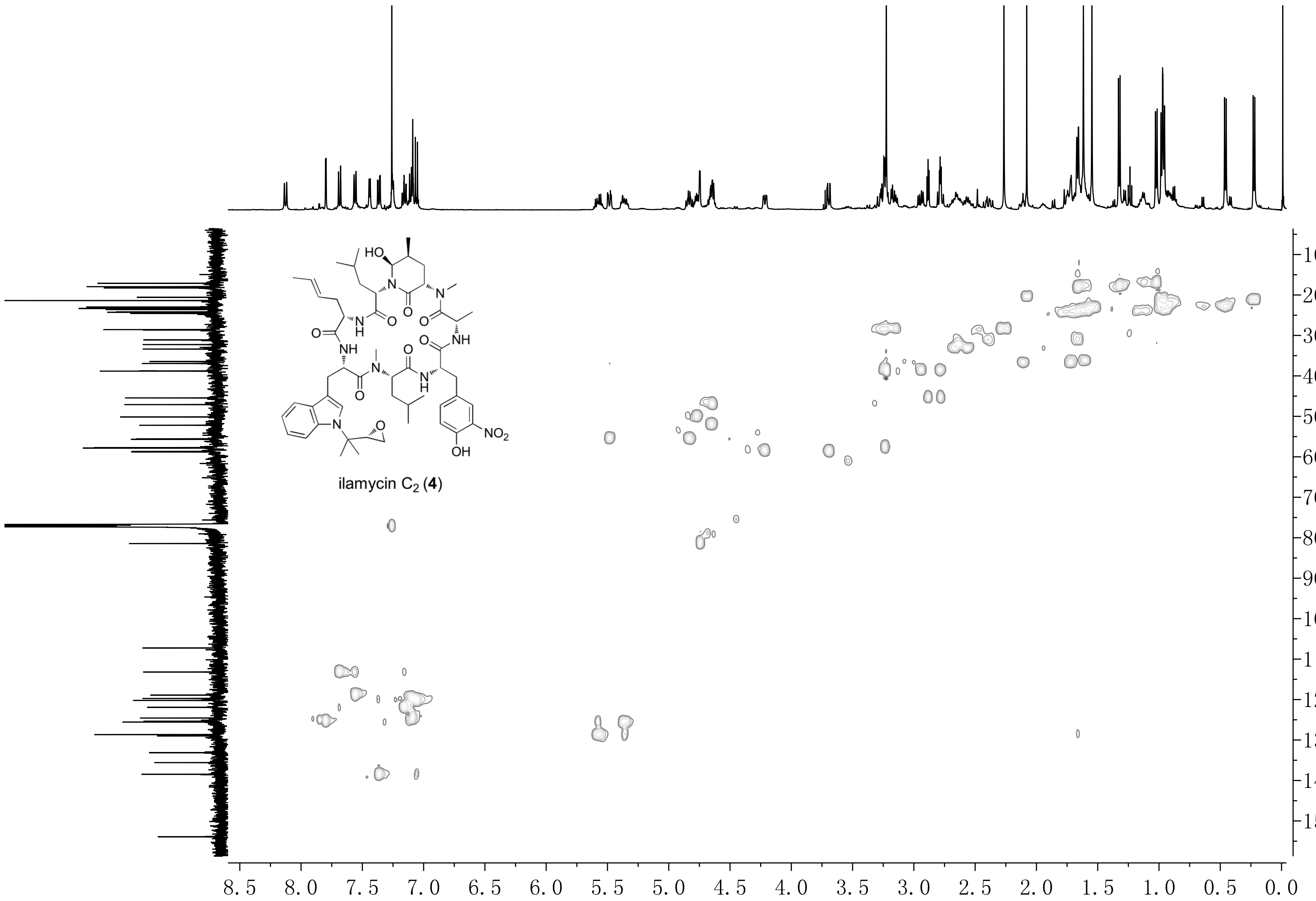

Supplementary Figure 48. HMQC spectrum of ilamycin C_2 (**4**) in $CDCl_3$.

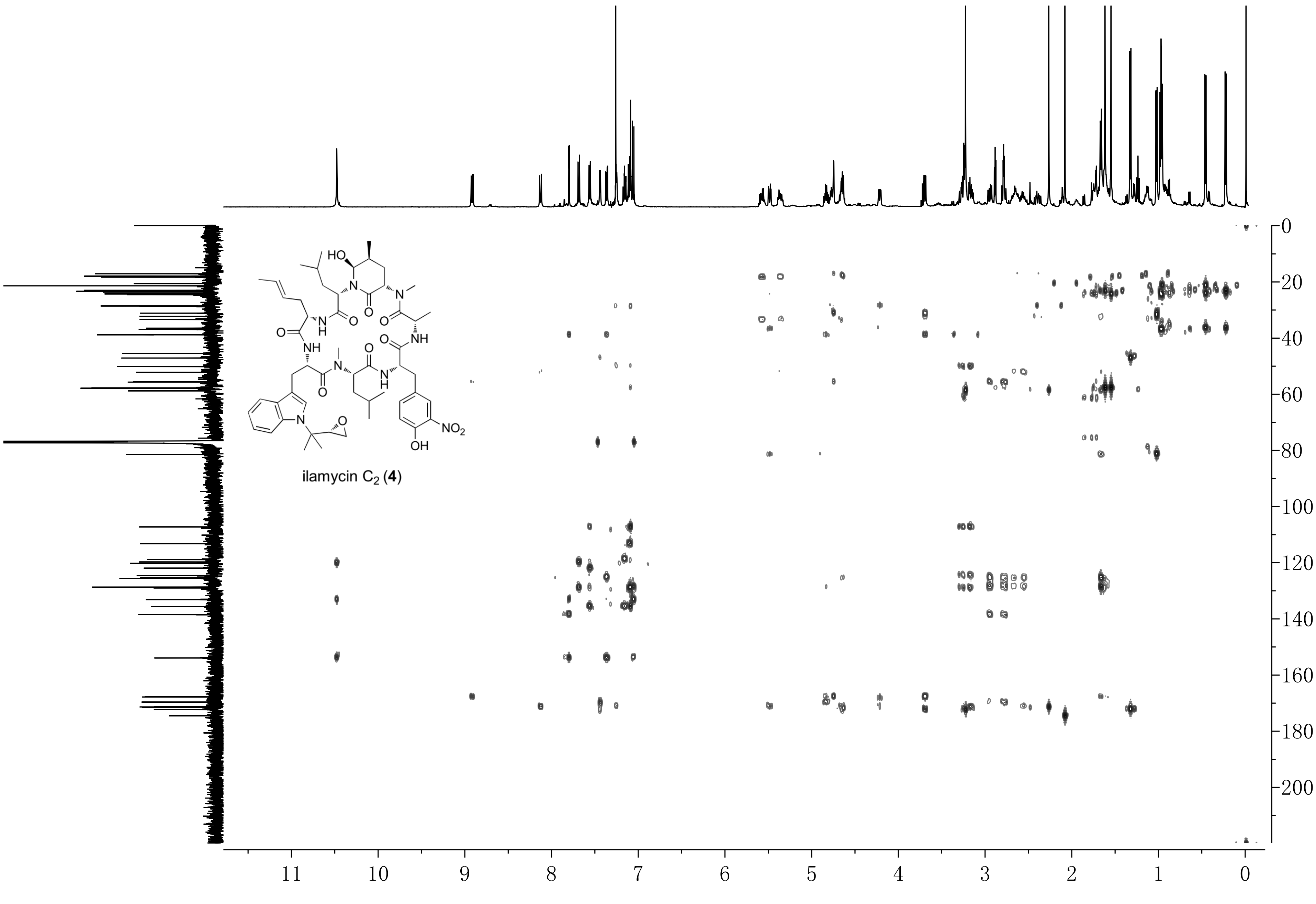

Supplementary Figure 49. HMBC spectrum of ilamycin C_2 (**4**) in $CDCl_3$.

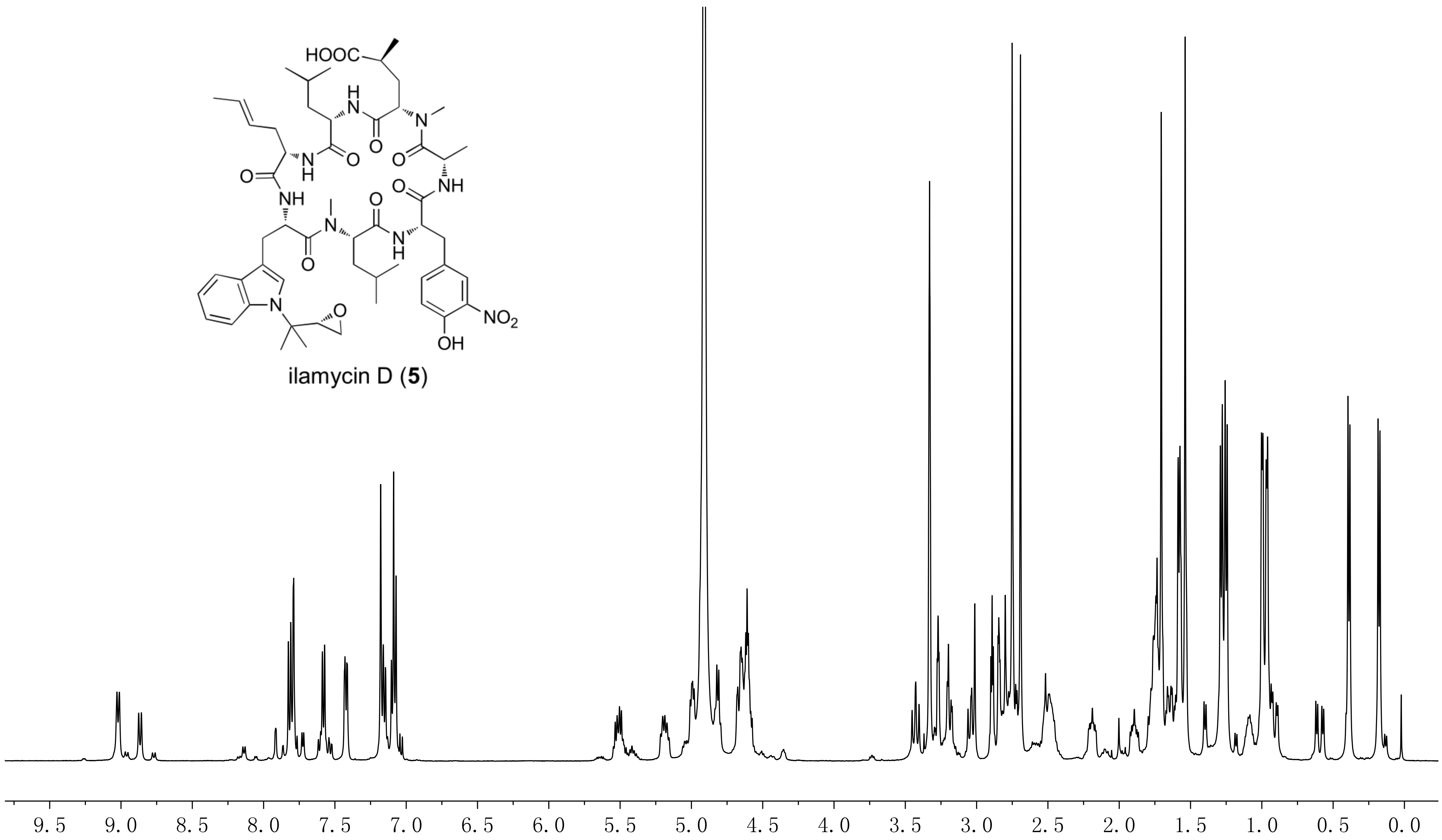

Supplementary Figure **50**. ^{1}H NMR spectrum of ilamycin D (**5**) in CD_3OD at 500 MHz.

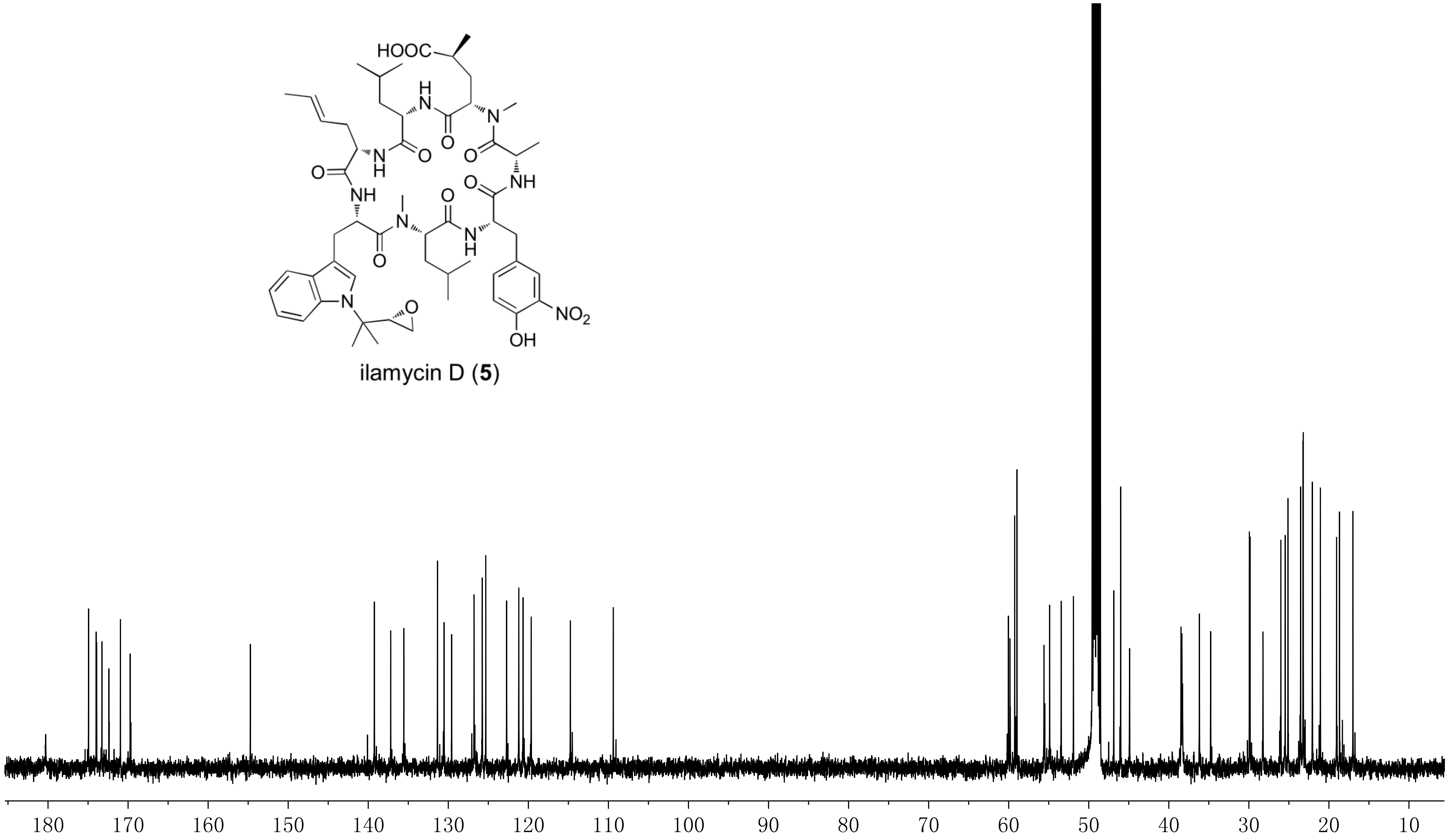

Supplementary Figure 51. ^{13}C NMR spectrum of ilamycin D (**5**) in CD_3OD at 125 MHz.

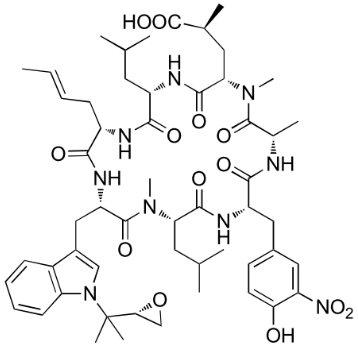

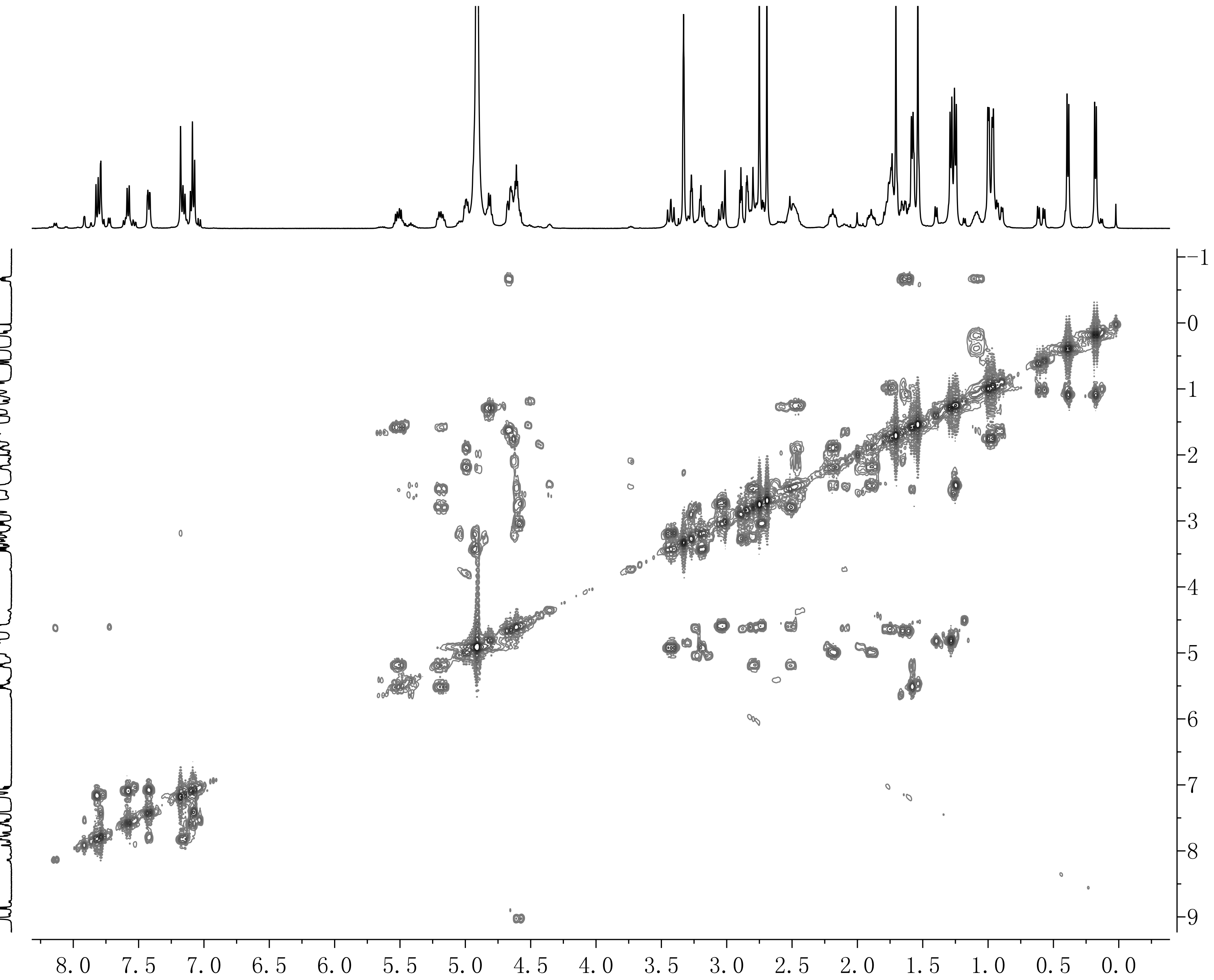

Supplementary Figure 52. COSY spectrum of ilamycin D (**5**) in CD_3OD.

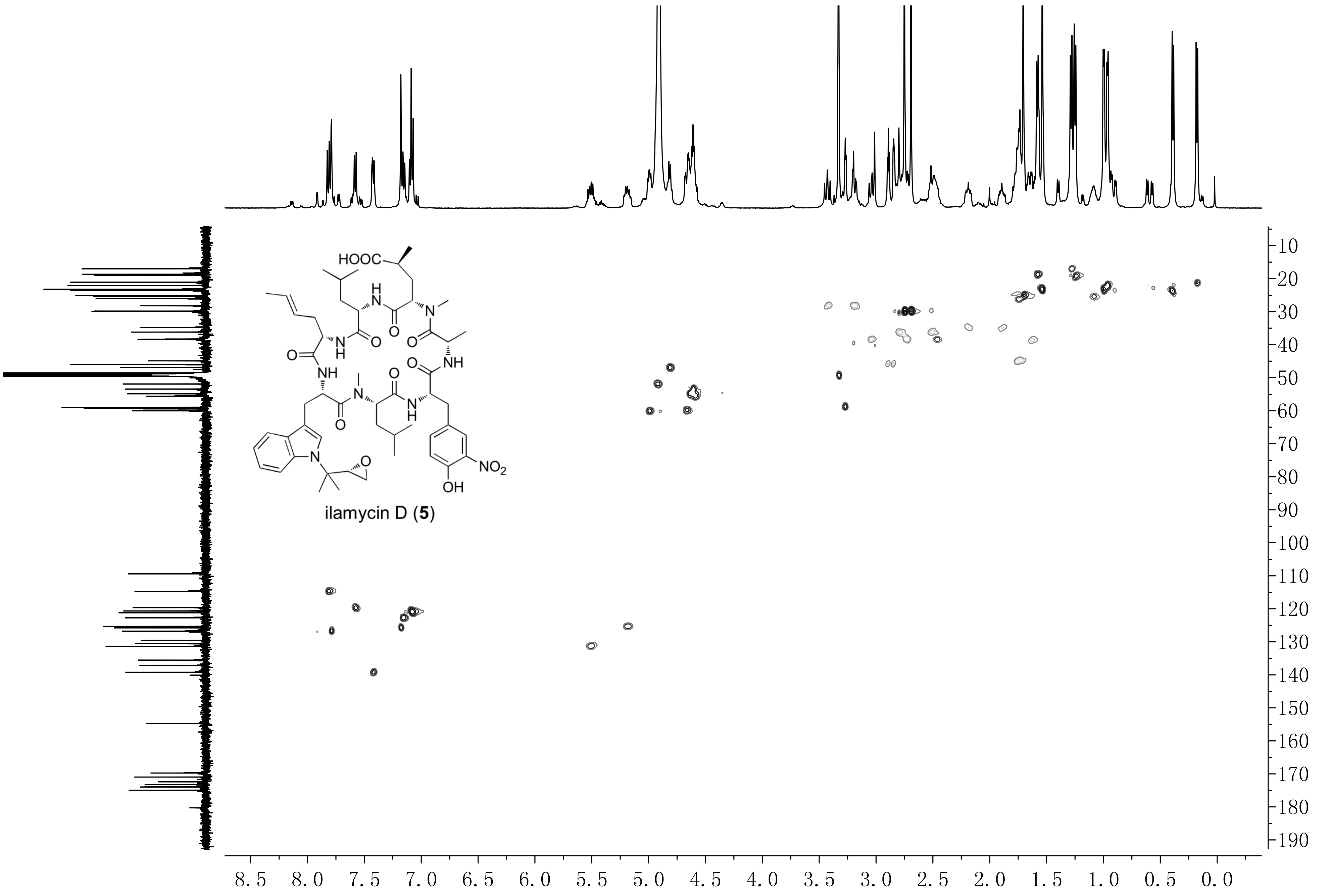

Supplementary Figure 53. HMQC spectrum of ilamycin D (**5**) in CD_3OD.

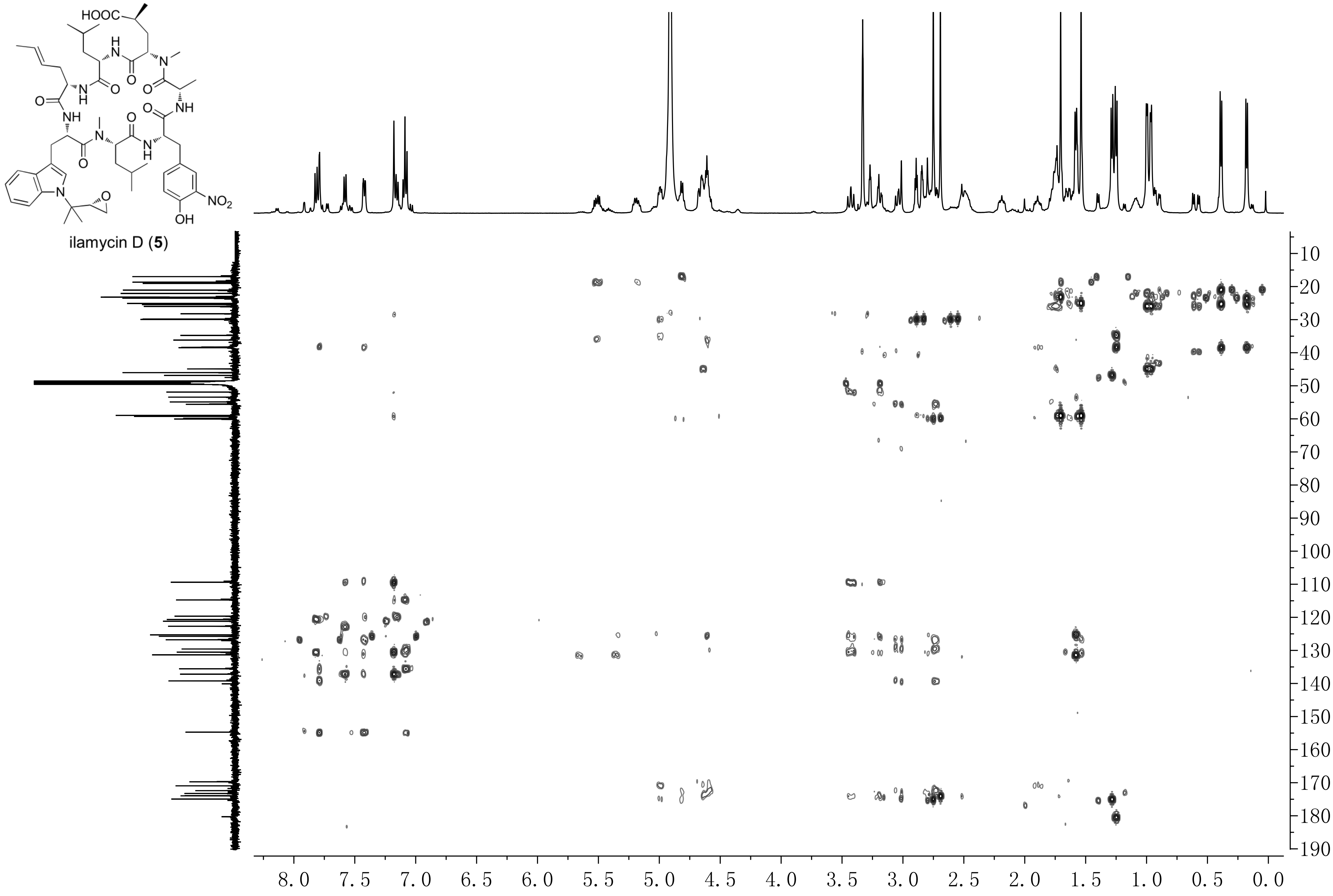

Supplementary Figure 54. HMBC spectrum of ilamycin D (**5**) in CD_3OD.

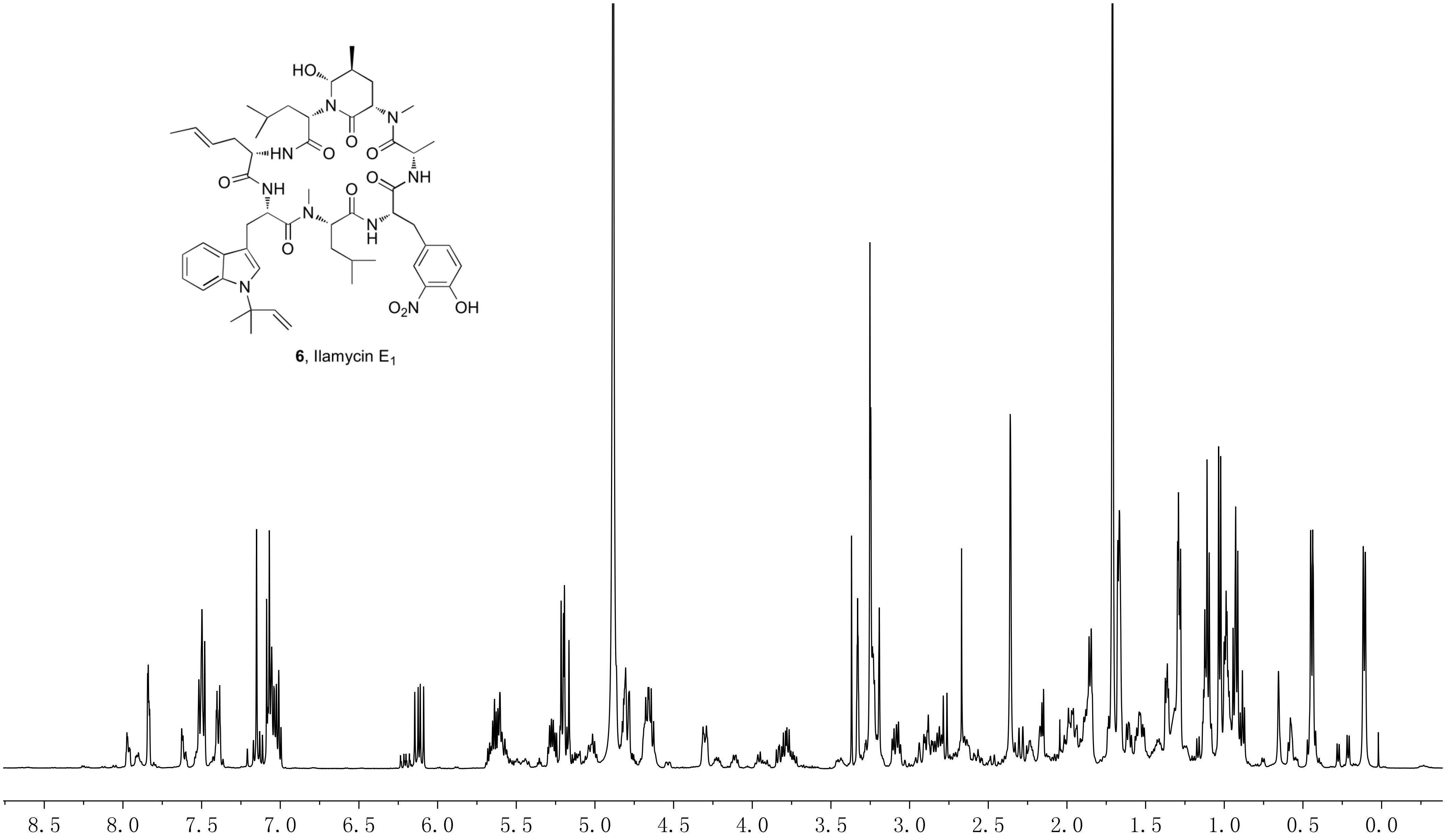

Supplementary Figure 55. ^{1}H NMR spectrum of ilamycin E_1 (**6**) in CD_3OD at 500 MHz.

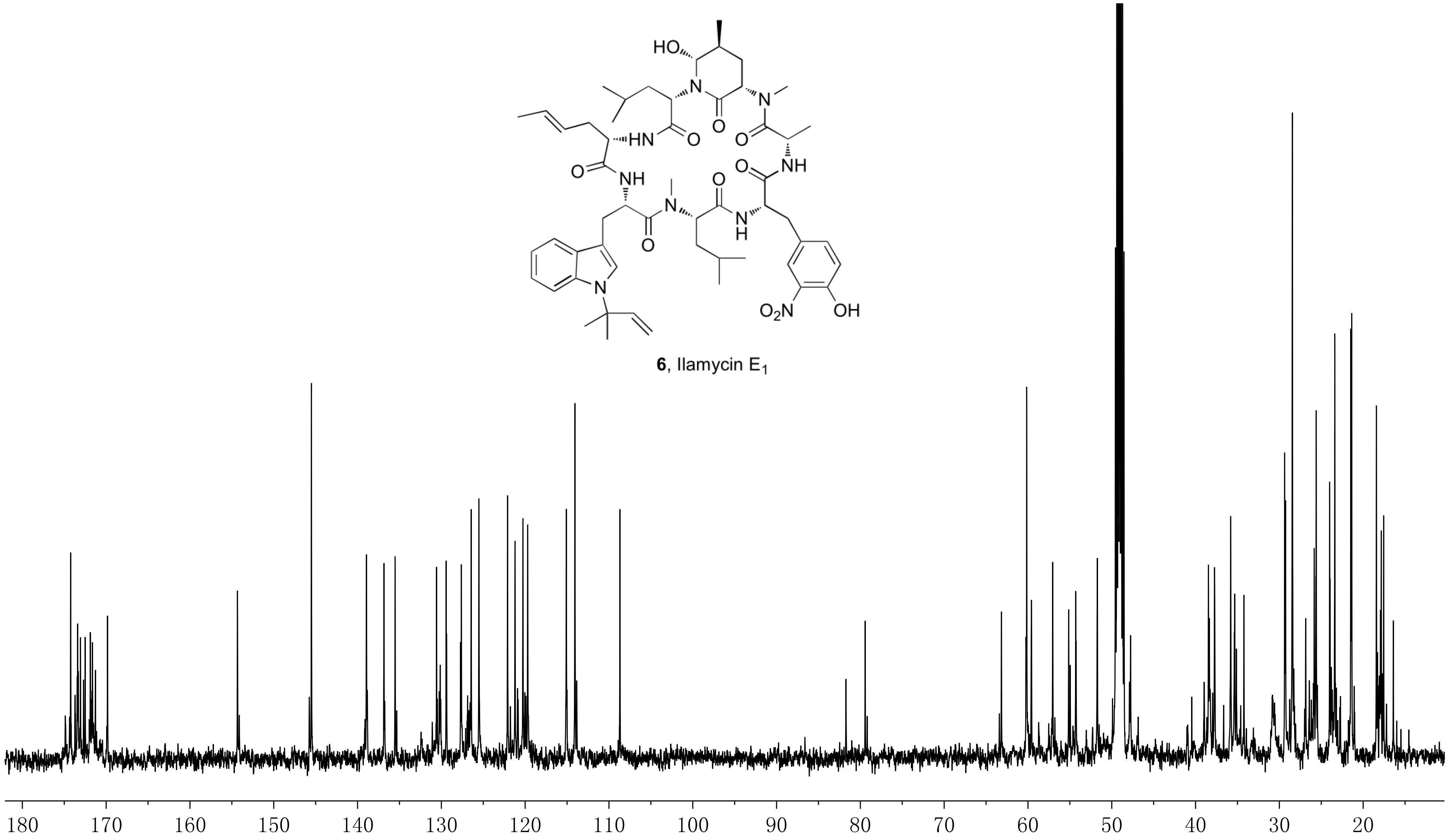

Supplementary Figure 56. ^{13}C NMR spectrum of ilamycin E_1 (**6**) in CD_3OD at 125 MHz.

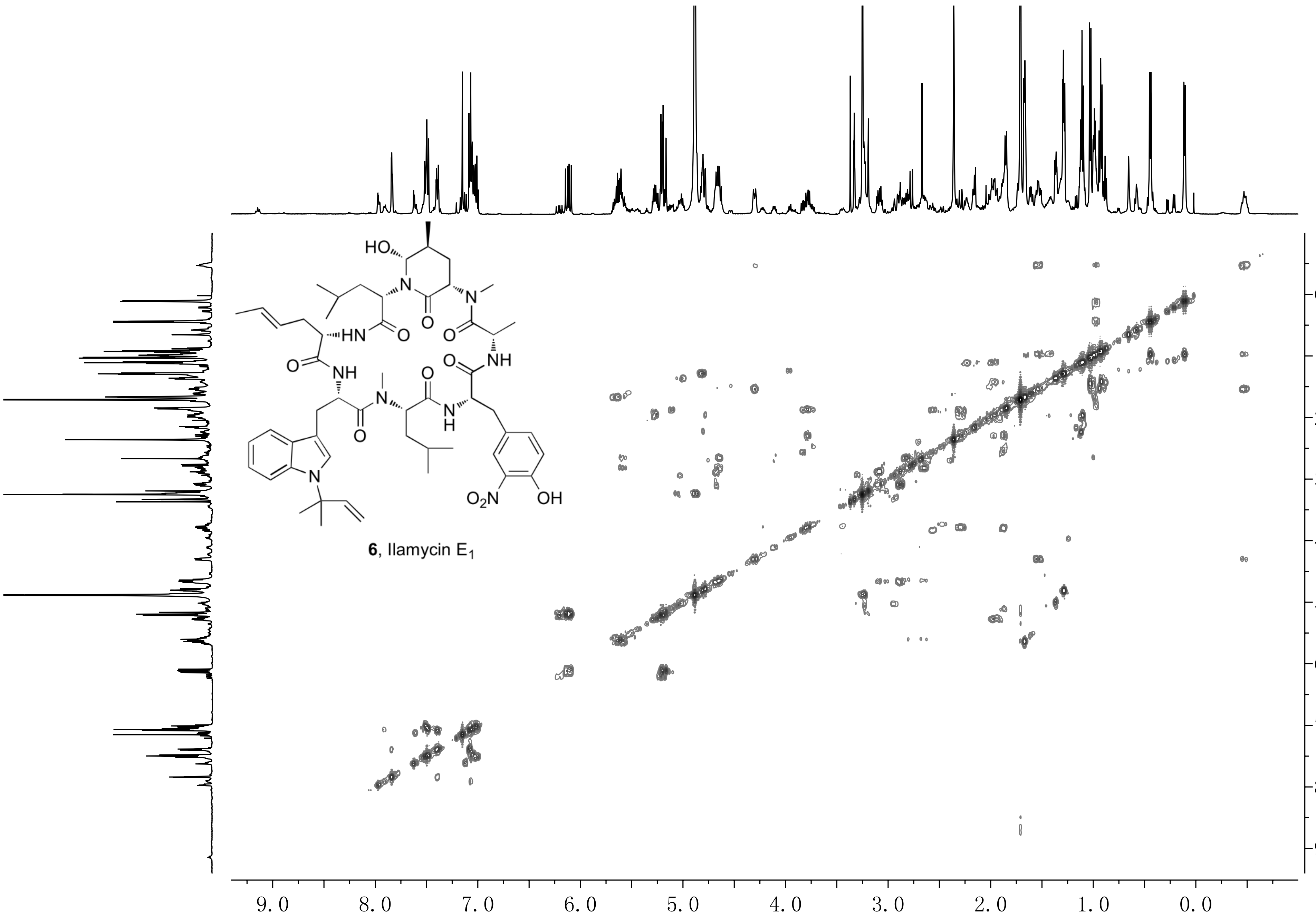

Supplementary Figure 57. COSY spectrum of ilamycin E_1 (**6**) in CD_3OD.

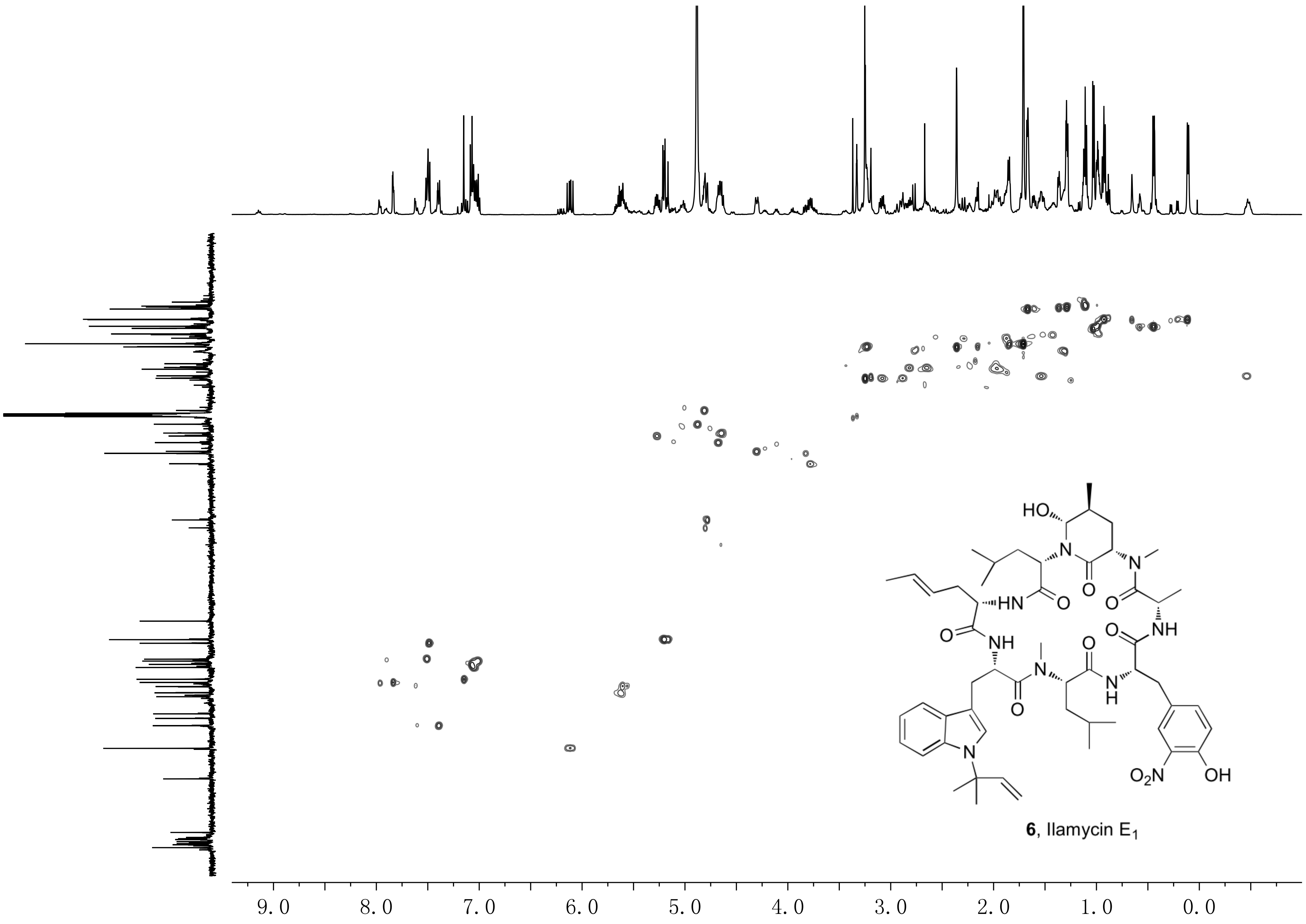

Supplementary Figure 58. HMQC spectrum of ilamycin E_1 (**6**) in CD_3OD.

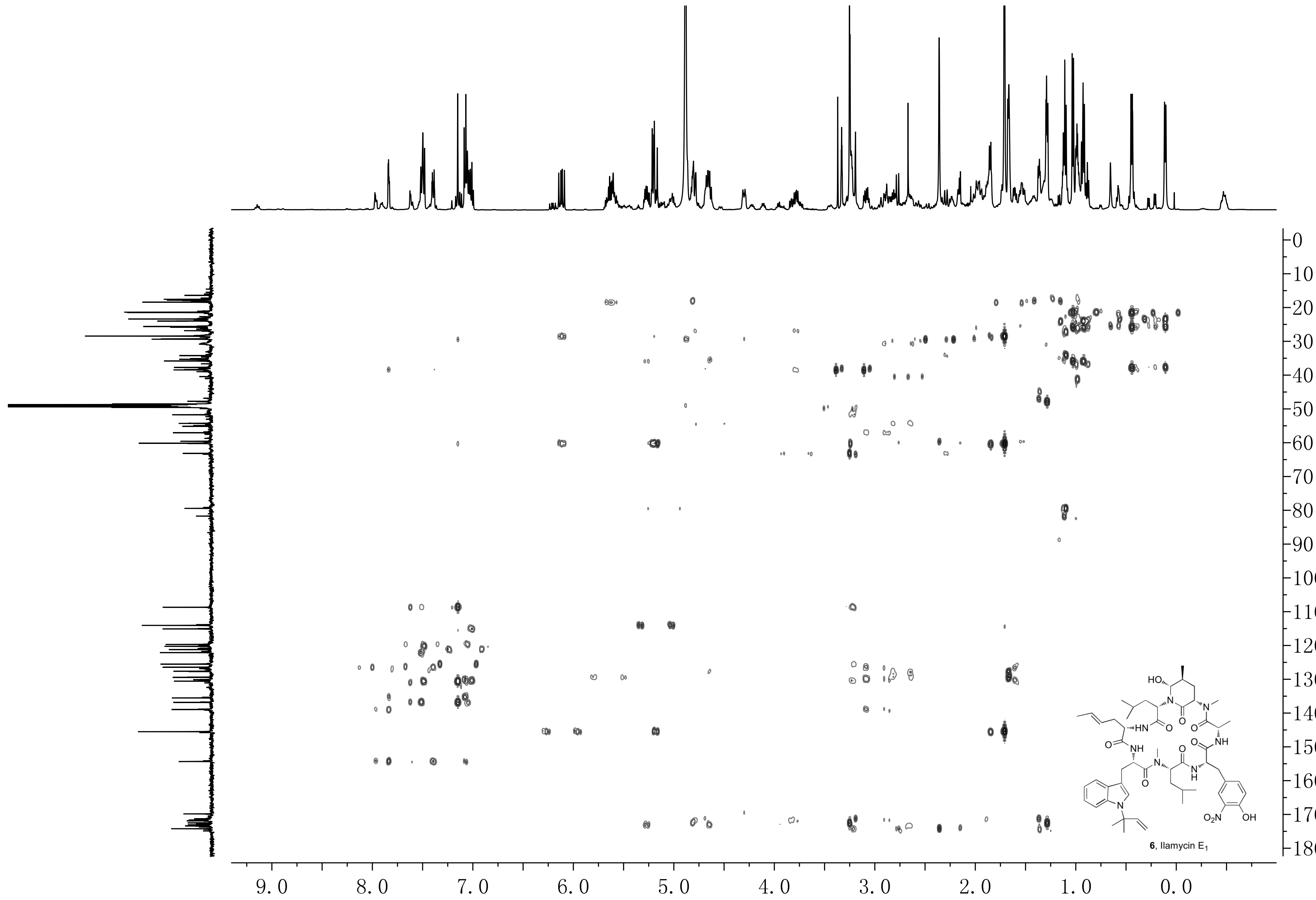

Supplementary Figure 59. HMBC spectrum of ilamycin E_1 (**6**) in CD_3OD.

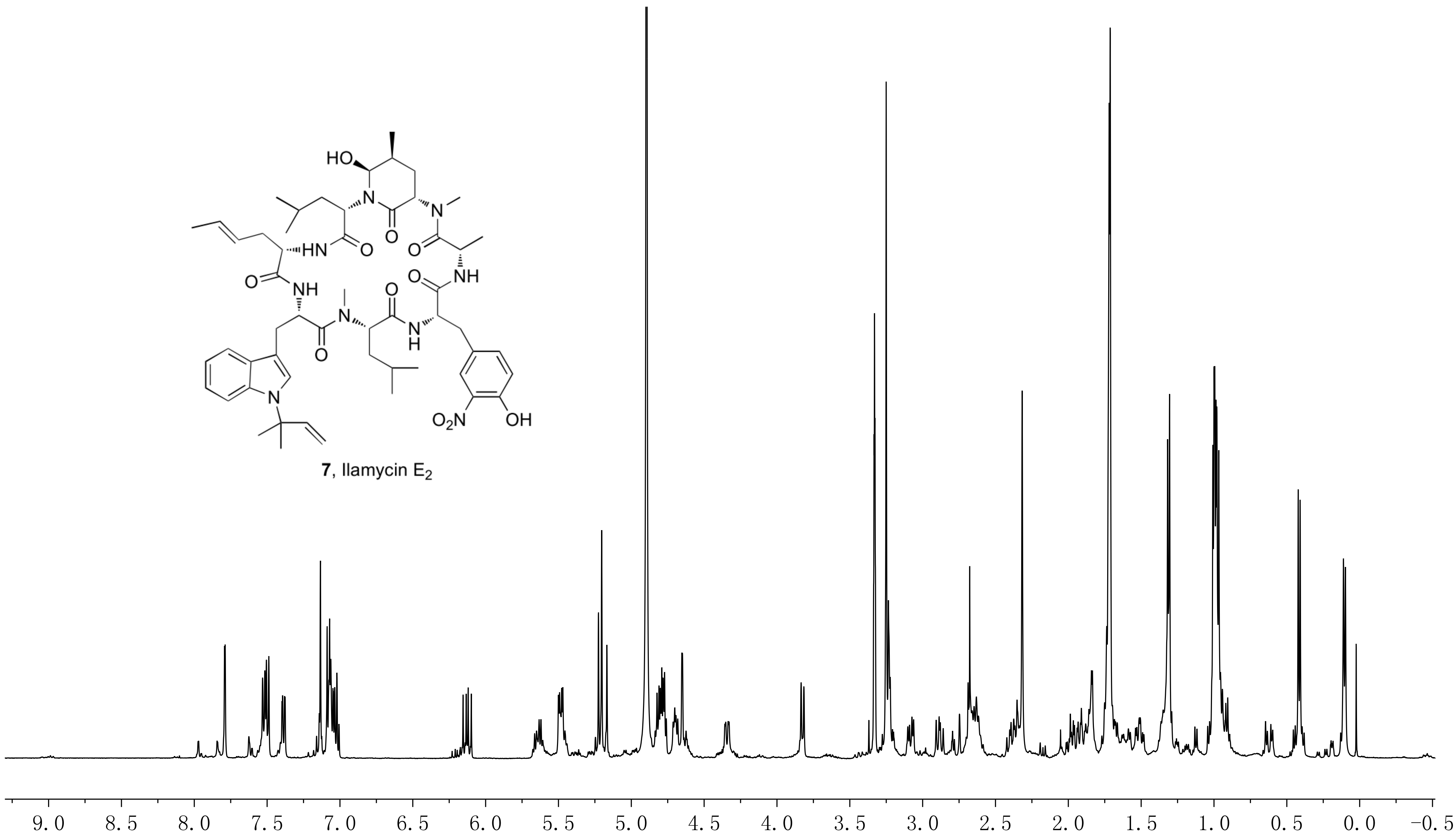

Supplementary Figure 60. ^{1}H NMR spectrum of ilamycin E_2 (**7**) in CD_3OD at 500 MHz.

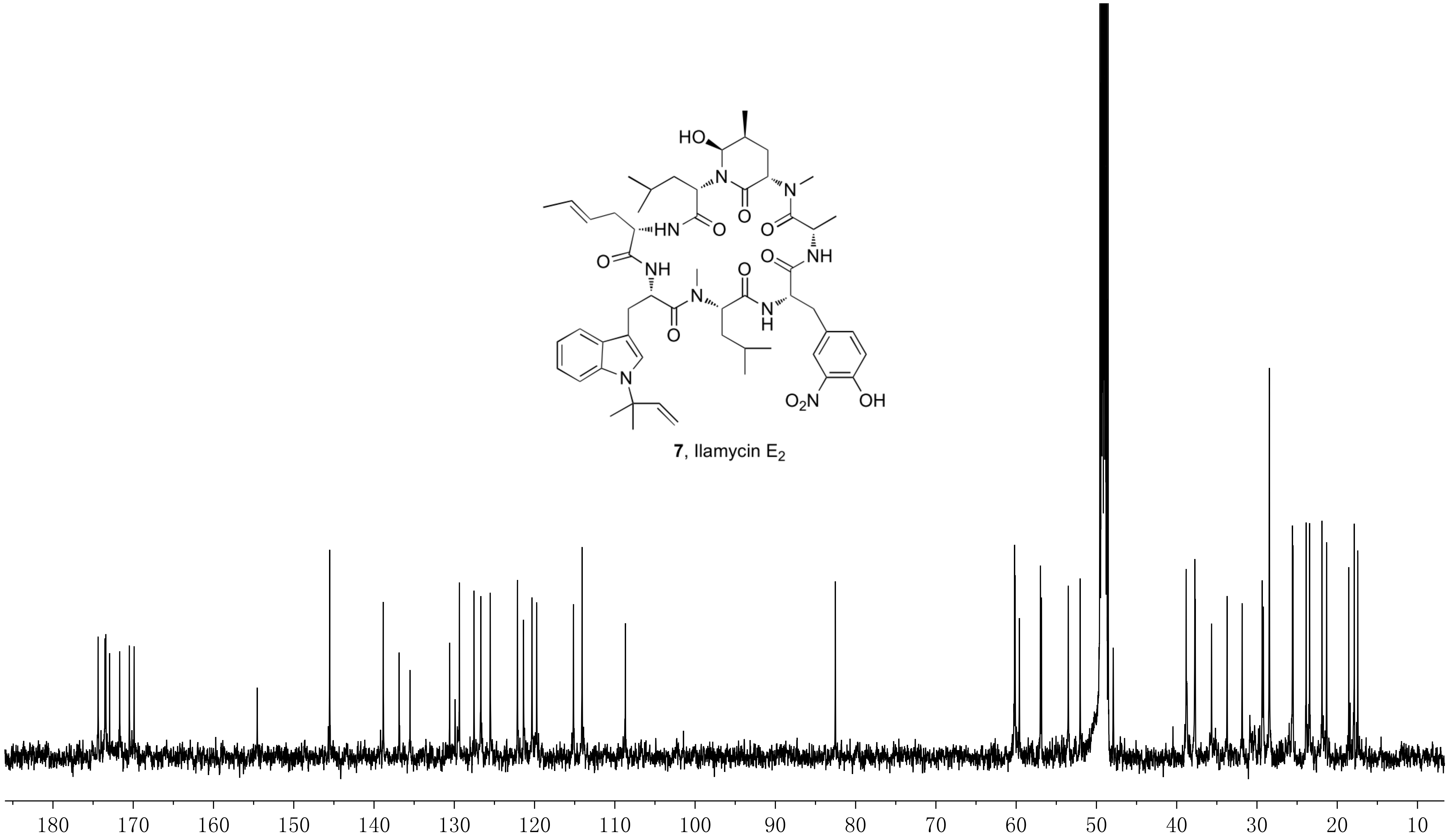

Supplementary Figure 61. ^{13}C NMR spectrum of ilamycin E_2 (**7**) in CD_3OD at 125 MHz.

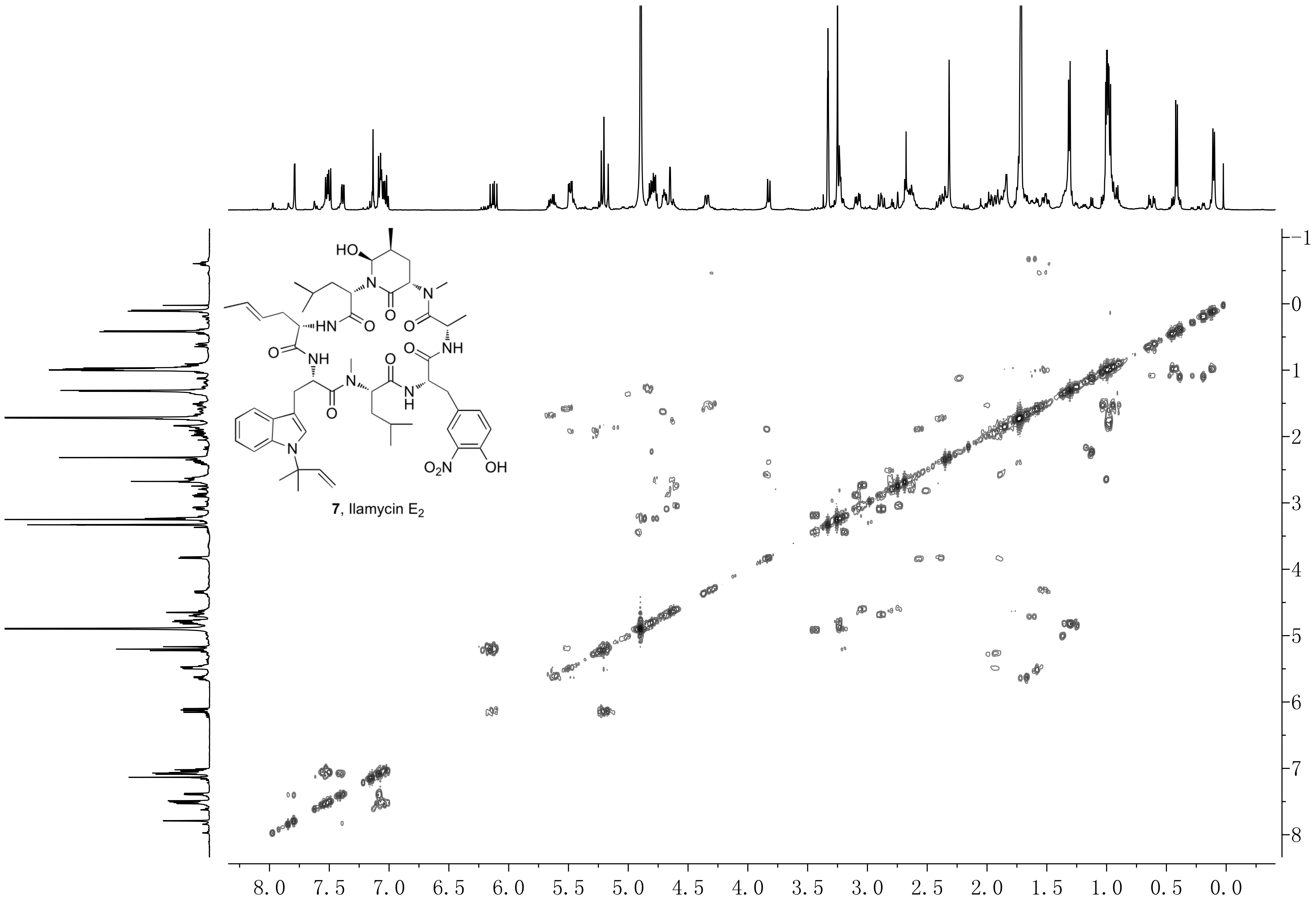

Supplementary Figure 62. COSY spectrum of ilamycin E_2 (**7**) in CD_3OD.

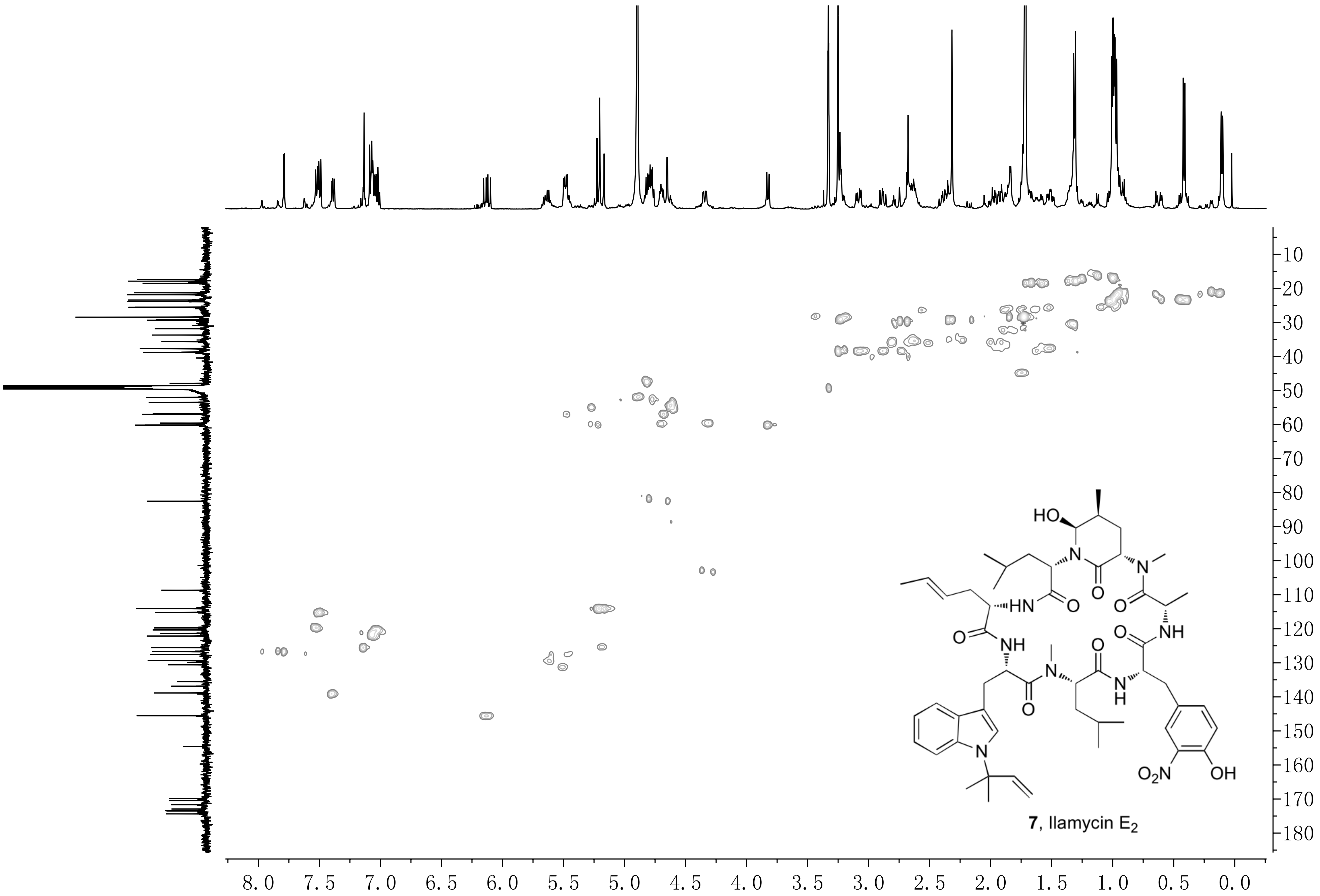

Supplementary Figure 63. HMQC spectrum of ilamycin E_2 (**7**) in CD_3OD.

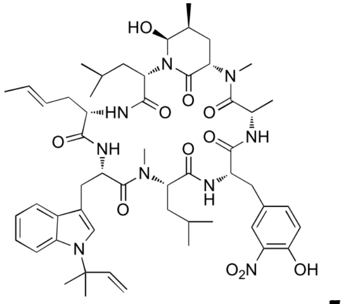

7, Ilamycin E_2

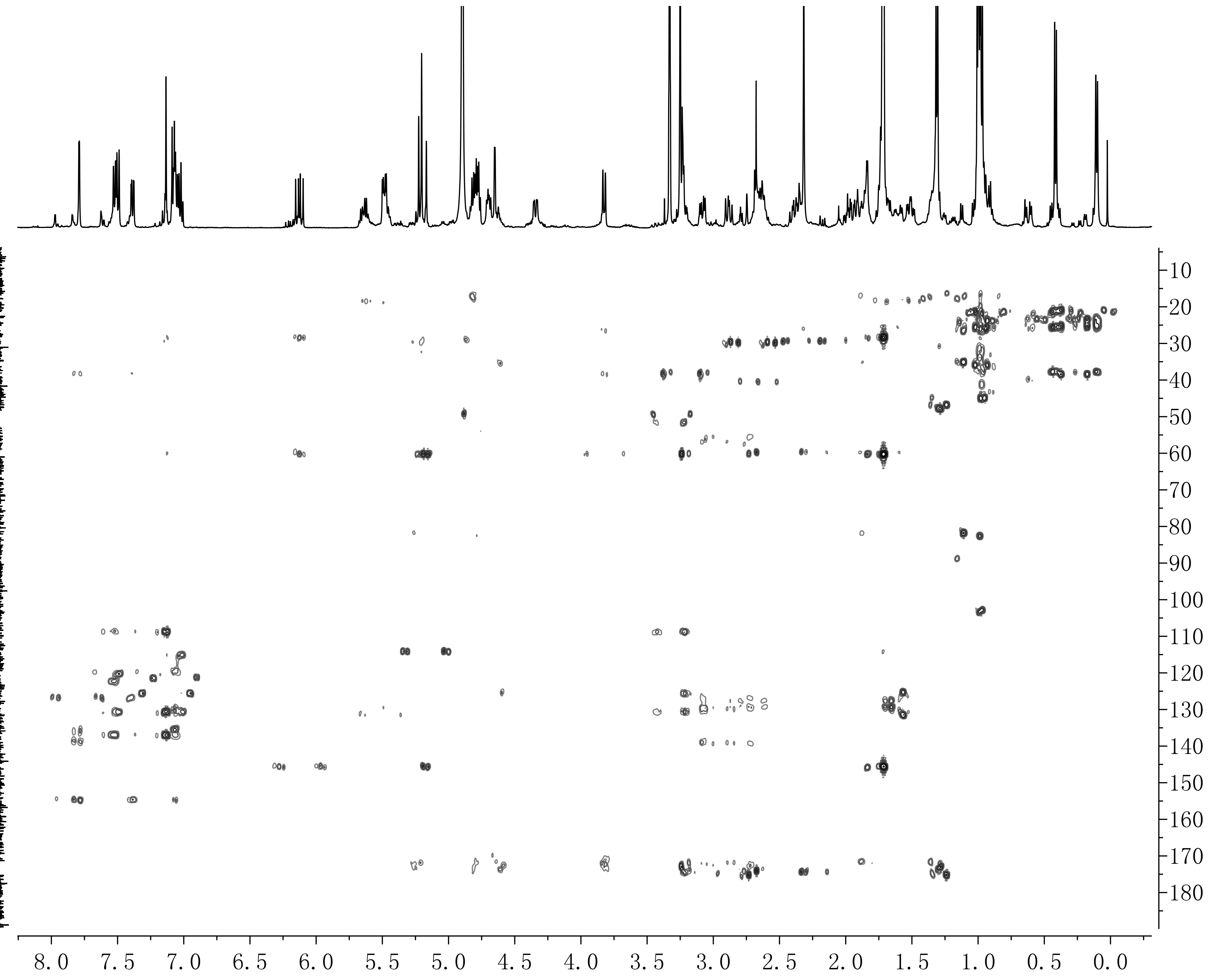

Supplementary Figure **64**. HMBC spectrum of ilamycin E_2 (**7**) in CD_3OD.

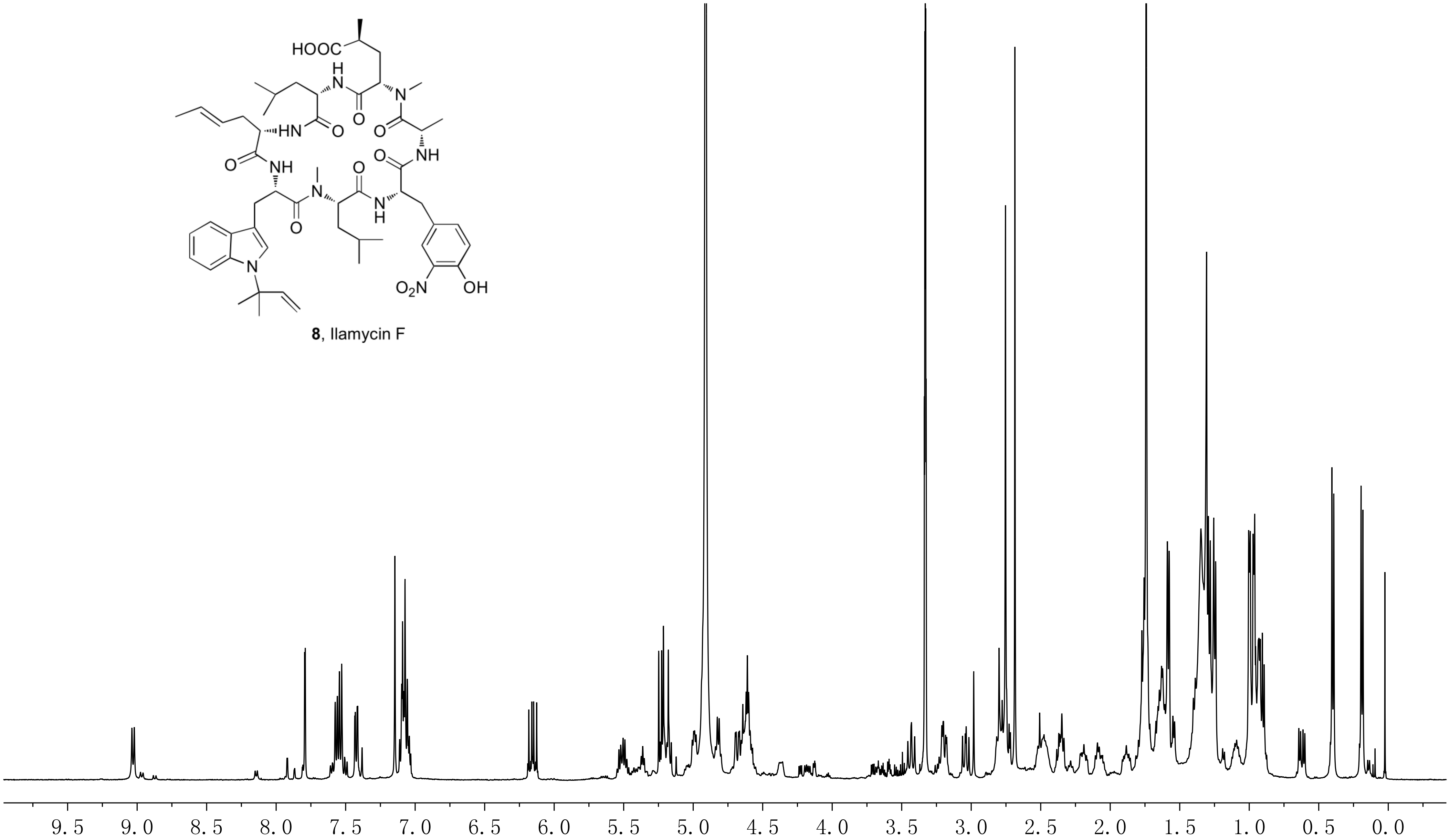

Supplementary Figure 65. ^{1}H NMR spectrum of ilamycin F (**8**) in CD_3OD at 500 MHz.

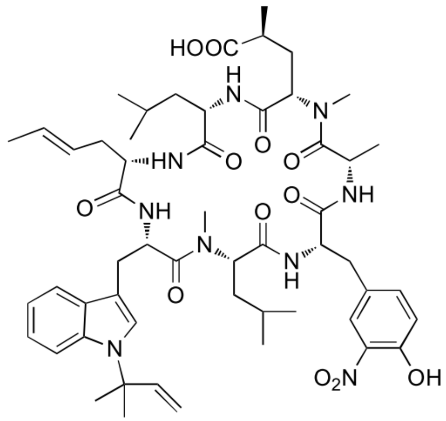

8, Ilamycin F

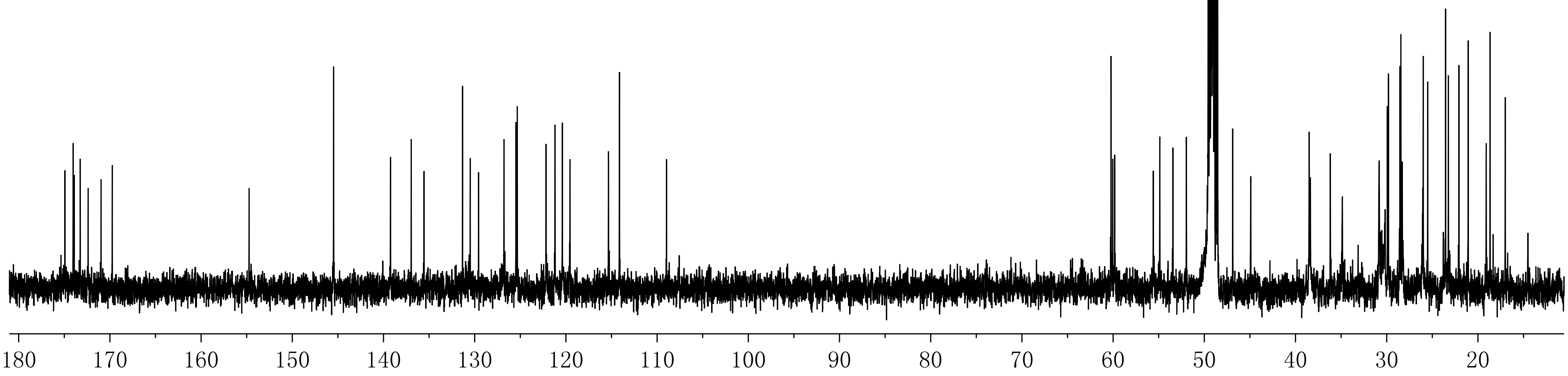

Supplementary Figure 66. ^{13}C NMR spectrum of ilamycin F (**8**) in CD_3OD at 125 MHz.

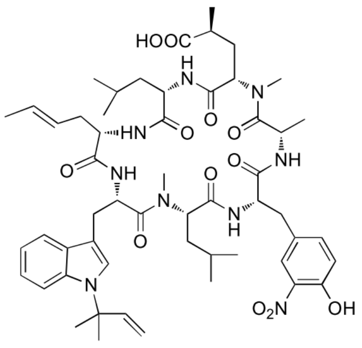

8, Ilamycin F

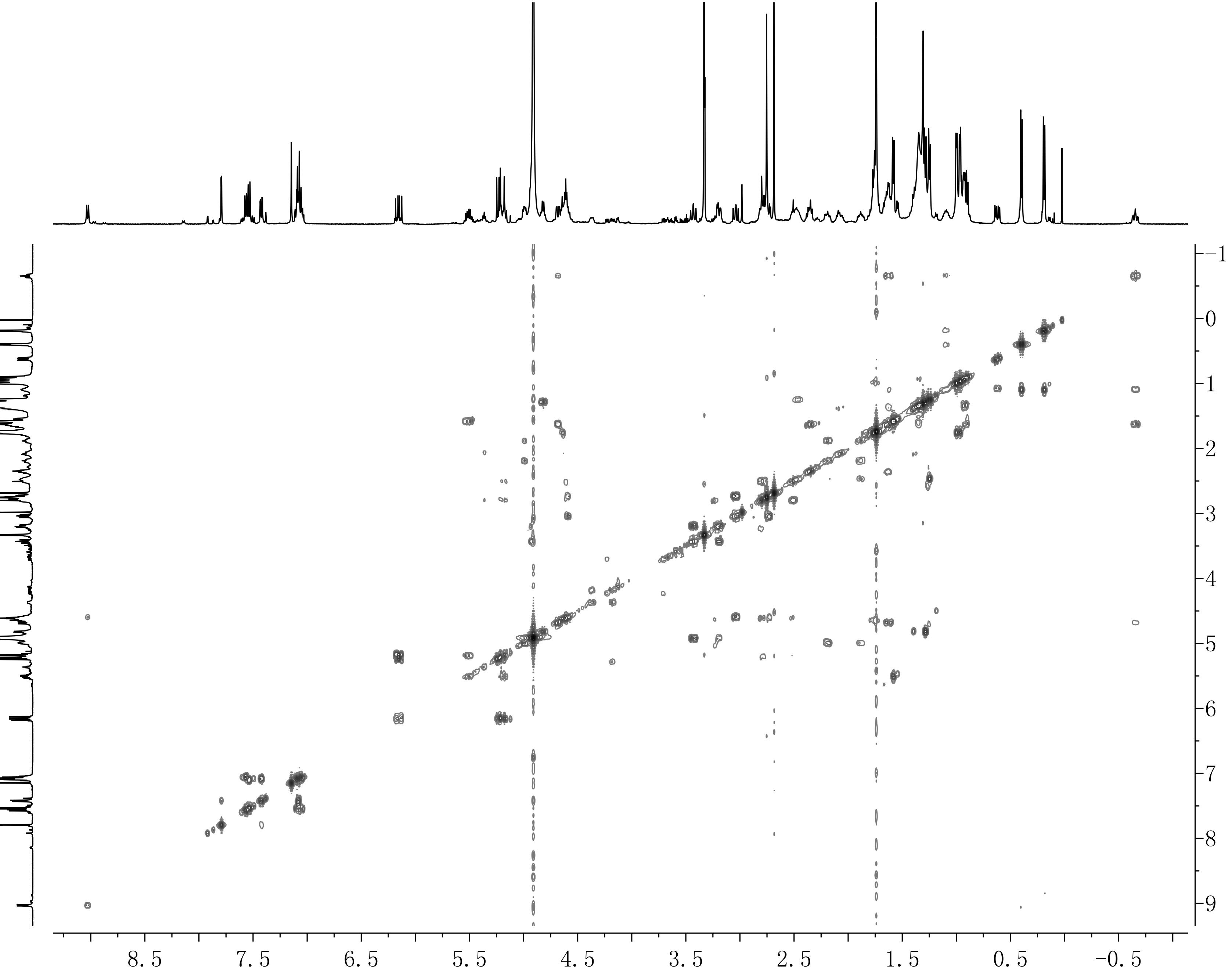

Supplementary Figure 67. COSY spectrum of ilamycin F (**8**) in CD_3OD.

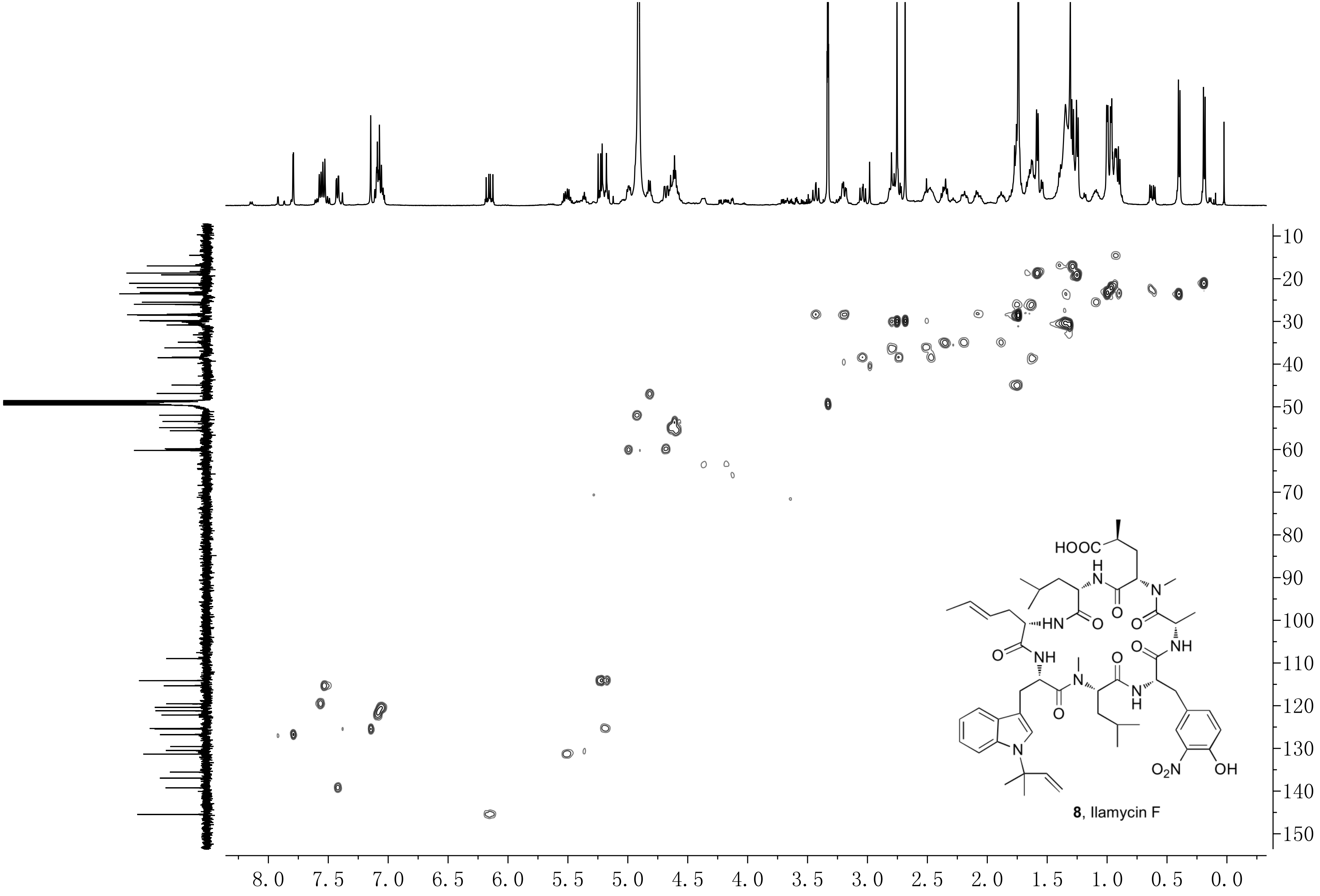

Supplementary Figure 68. HMQC spectrum of ilamycin F (**8**) in CD_3OD.

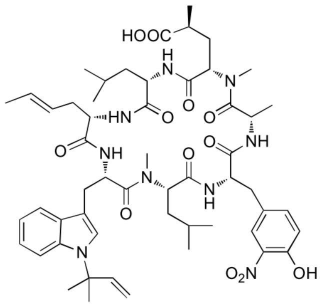

8, Ilamycin F

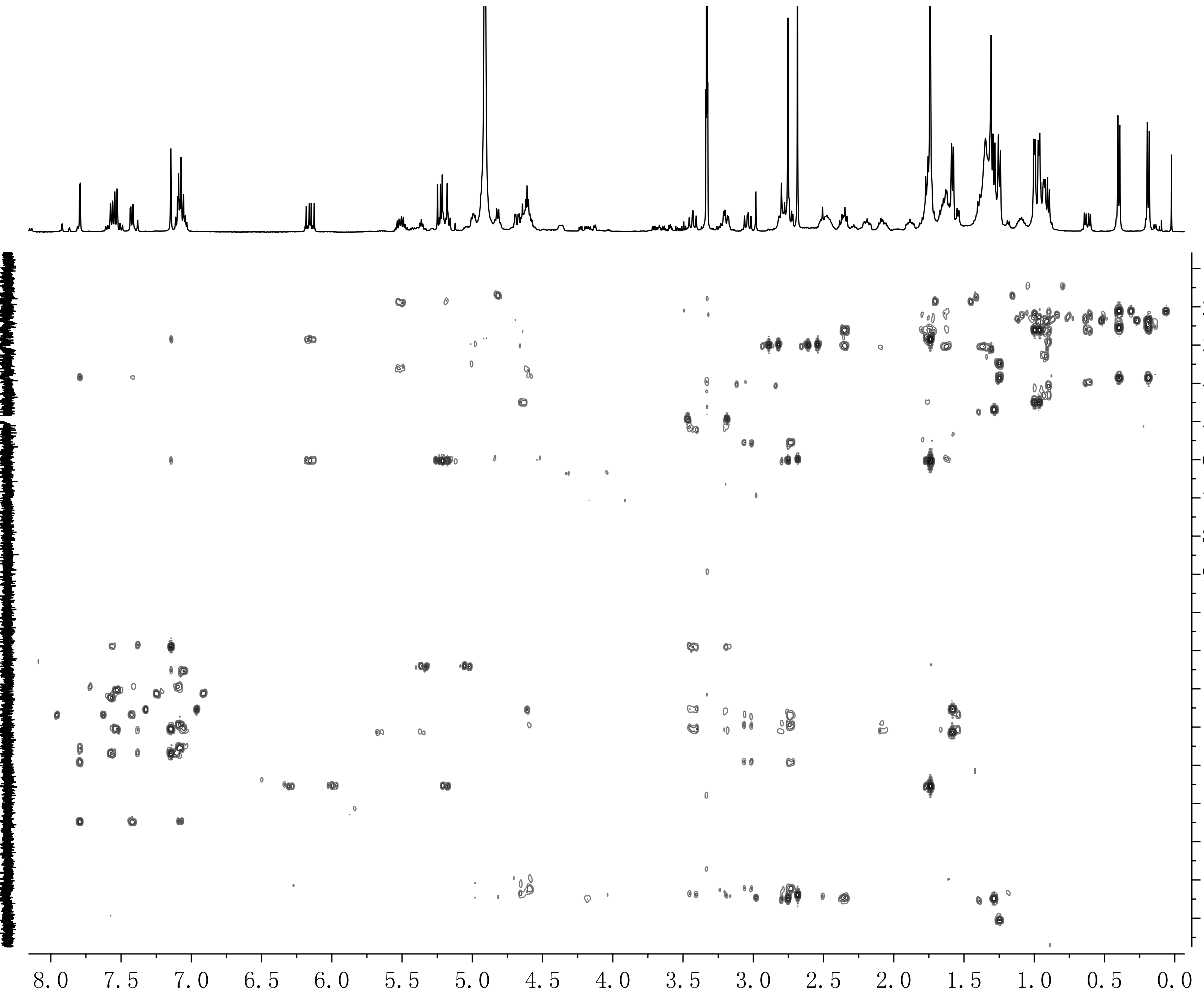

Supplementary Figure 69. HMBC spectrum of ilamycin F (**8**) in CD_3OD.

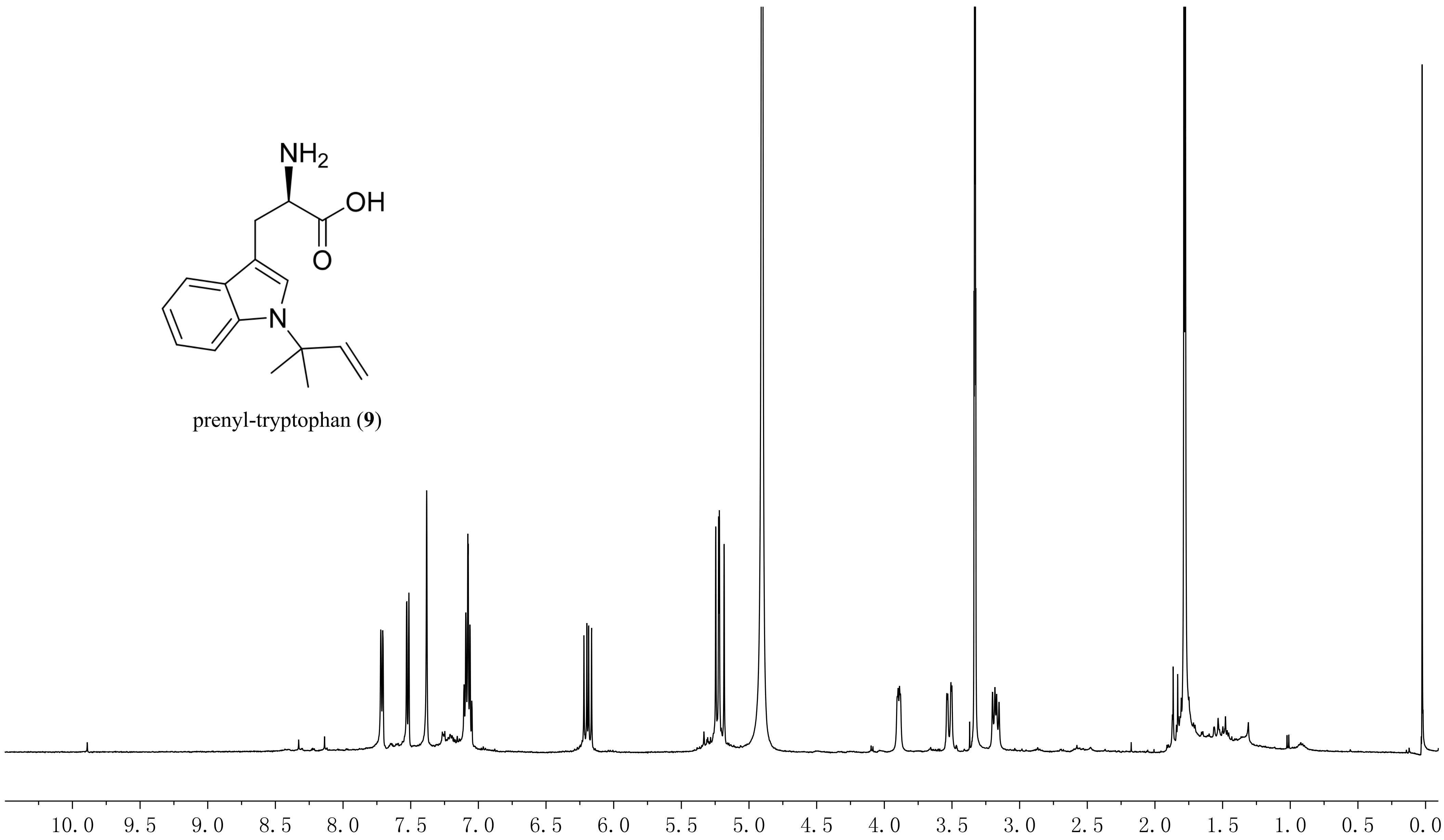

Supplementary Figure 70.^{1}H NMR spectrum of prenyl-tryptophan (**9**) in CD_3OD at 500 MHz.

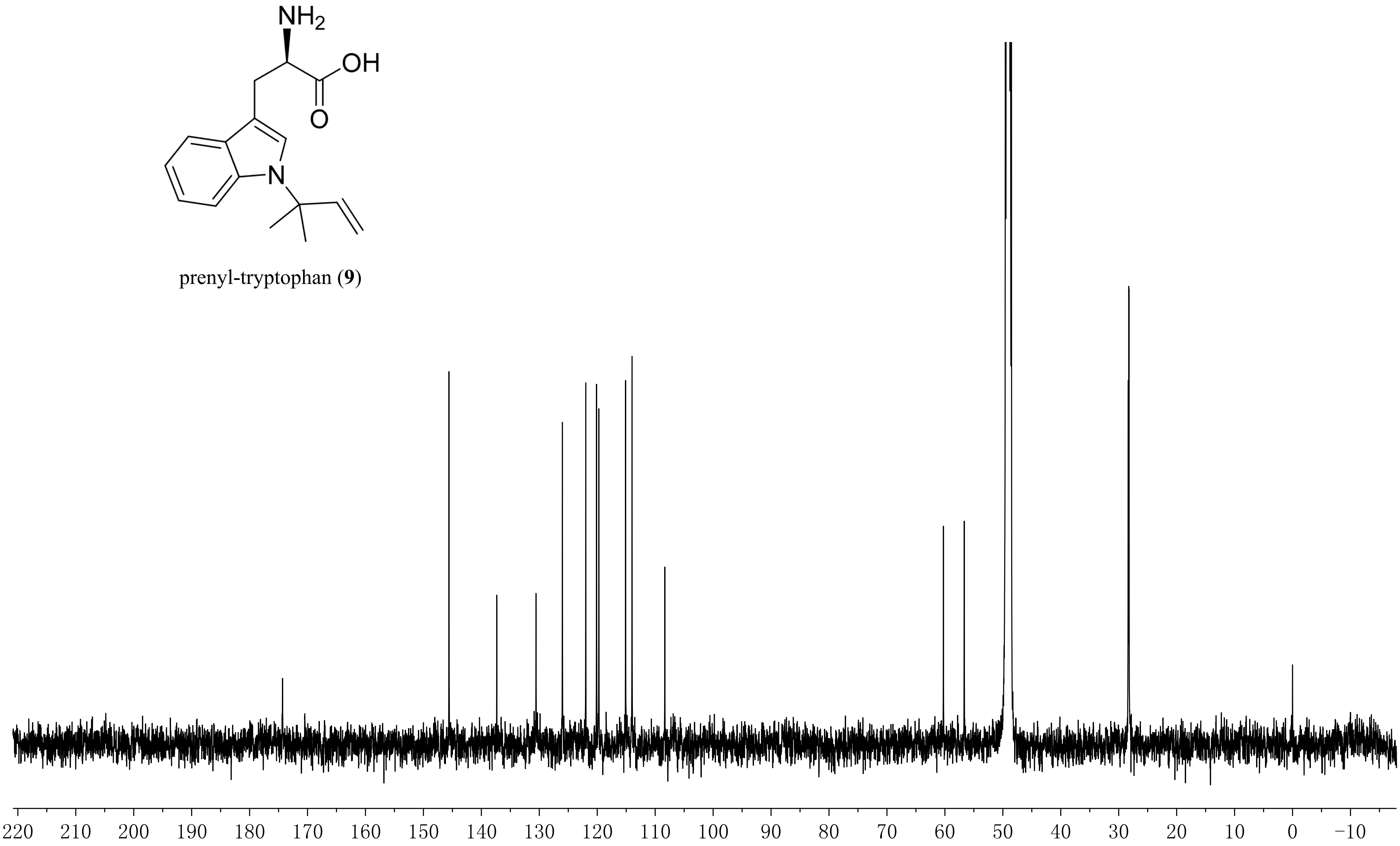

prenyl-tryptophan (**9**)

Supplementary Figure 71. ^{13}C NMR spectrum of prenyl-tryptophan (**9**) in CD_3OD at 125 MHz.

Supplementary Table 1. Crystal structure data and structure refinement for compounds **2**, **4**, **5** and **8**.

	Ilamycin B_2 (**2**)	Ilamycin C_2 (**4**)	Ilamycin D (**5**)	Ilmycin F (**8**)
Empirical fromula	$C_{54}H_{77}N_9O_{11}$	$C_{54}H_{75}N_9O_{12}$ 2 ($CHCl_3$) 2 (CH_3OH)	$C_{54}H_{75}N_9O_{13}$	$C_{54}H_{75}N_9O_{12}$
Formula weight	1028.25	1345.05	1058.23	1042.23
Temperature	293 (2)K	293 (2) K	150 (14) K	293 (2) K
Wavelength	0.71073	1.54184	1.54184	1.54184
Crystal system	orthorhombic	orthorhombic	orthorhombic	orthorhombic
Space group	$P2_12_12$	$P2_12_12_1$	$P2_12_12_1$	$P2_12_12_1$
Unit cell dimensions	a=18.3538(2) Å α=90°	a=17.30911(13) Å α=90 °	a=15.8709(2) Å α=90 °	a=17.10632(12) Å α=90 °
	b= 28.2850(3) Å β=90°	b=17.89029(14) Å β=90 °	b=18.8030(3) Å β=90 °	b=18.5208(2) Å β=90 °
	c=13.32010(10) Å γ= 90°	c=21.67363(19) Å γ= 90 °	c=22.3506(3) Å γ= 90 °	c=39.2320(3) Å γ= 90 °
Volume	6914.96(12) Å	6711.56(9) Å	6669.88(16) Å	12429.60(18) Å
Z	4	4	4	8
Density (calculated)	0.988 g/cm^3	1.331 g/cm^3	1.054 g/cm^3	1.114 g/cm^3
Absorption coefficient	0.070 mm^{-1}	2.892 mm^{-1}	0.624 mm^{-1}	0.651 mm^{-1}
F(000)	2208.0	2840.0	2264.0	4464.0
Crystal size	0.3×0.2×0.1 mm^3	0.3×0.2×0.2 mm^3	0.3×0.2×0.2 mm^3	0.3×0.3×0.2 mm^3
Index ranges	-21<=h<=21,	-21<=h<=21,	-18<=h<=11	-20<=h<=20
	-33<=k<=34,	-22<=k<=20,	-21<=k<=22	-22<=k<=22
	-16<=l<=16	-26<=l<=24	-26<=l<=26	-46<=l<=46
Reflections collected	63230	66091	18906	110247
Independent reflections	13266 (R_{int}=0.0478)	13122 (R_{int}=0.0336)	11046 (R_{int}=0.0323)	22349 (R_{int}=0.0608)
Completeness to theta	0.999	1.000	0.999	1.000
Absorption correction	Multi-scan	Multi-scan	Multi-scan	Multi-scan
Max. and min. transmission	1.000 and 0.355	1.00 and 0.8110	1.000 and 0.8040	1.000 and 0.1350
Refinement method	ShelXL (Sheldrick 2015)	ShelXL (sheidrick 2008)	ShelXL (sheidrick 2008)	ShelXL (sheidrick 2008)
Data/Restraints/parameters	13266/0/668	13112/12/810	11046/2/729	22349/0/1377
Goodness-of-fit on F^2	1.037	1.023	1.108	1.02
Final R indices [I>2sigma(I)]	R_1=0.0668,	R_1=0.0458,	R1=0.0699,	R1=0.0652,
	ωR_2=0.1997	ωR_2=0.1237	ωR2=0.2046	ωR2=0.1831
R indices (all data)	R_1=0.0736,	R_1=0.0484,	R_1=0.0916	R_1=0.0726
	ωR2=0.2098	ωR2=0.1273	ωR2=0.2390	ωR2=0.1913
Absolute structure parameter	-1.9(5)	-0.012(3)	0.1(4)	0.14(5)
Extinction coefficient	n/a	0.00034(8)	0.0006(2)	n/a
Largest diff. peak and hole	0.523 and -0.240 e. Å^{-3}	1.086 and -0.687 e. Å^{-3}	0.542 and -0.266 e. Å^{-3}	0.880 and -0.349 e. Å^{-3}

Crystallographic data have been deposited in the Cambridge Crystallographic Data Center with the deposition number CDCC1524774 for **2**, CDCC1524775 for **4**, CDCC1524776 for **5**, and CDCC1524777 for **8**, respectively. A copy of the data can be obtained, free of charge, on application to the Director, CCDC, 12 Union Road, Cambridge CB2 1EZ, UK [fax: +44(0)-1233-336033 or e-mail: deposit@ccdc.cam.ac.uk].

Supplementary Table 2. ^{1}H and ^{13}C NMR spectroscopic data for compounds **4**-**9**.

position	Ilamycin C_2 (**4**) [a]		Ilamycin D (**5**) [b]		Ilamycin E_1 (**6**) [b]		Ilamycin E_2 (**7**) [b]		Ilamycin F (**8**) [b]		Prenyl-tryptophan (**9**) [b]	
	δ_C	δ_H, multi. (*J* in Hz)	δ_C	δ_H, multi. (*J* in Hz)	δ_C	δ_H, multi. (*J* in Hz)	δ_C	δ_H, multi. (*J* in Hz)	δ_C	δ_H, multi. (*J* in Hz)	δ_C	δ_H, multi. (*J* in Hz)
Pre-Trp												
1	171.3		173.9		174.2		174.4		173.9		174.3	
2	50.1	4.78, dd (10.0, 5.0)	51.9	4.93, dd (11.2, 4.3)	51.7	4.88, m	52.1	4.86, m	52.0	4.90, overlapped	56.7	3.90, dd (8.2, 3.1)
3	28.7	3.28, dd (13.6, 5.0); 3.17, dd (13.6, 10.0)	28.3	3.45, dd (13.1, 4.3)	29.3	3.23, m	29.3	3.40, m: 3.23, m	28.3	3.46, dd (12.3, 11.5); 3.20, dd (12.3, 4.1)	28.2	3.54, dd (15.1, 3.1); 3.18, dd (15.1, 8.2)
4	124.6	7.09, s	125.8	7.17, s	125.5	7.15, s	125.5	7.14, s	125.3	7.15, s	126.0	7.38, s
5	107.3		109.5		108.7		108.7		109.0		108.3	
6	129.0		130.6		130.6		130.5		129.6		130.6	
7	118.9	7.57, d (7.7)	119.6	7.59, d (8.0)	119.7	7.52, d (8.1)	119.7	7.53, d (8.1)	119.6	7.57, d (7.7)	119.7	7.72, d (7.2)
8	119.7	7.11, m	120.6	7.11,m	120.3	7.04, d (8.1)	120.3	7.04, d (8.1)	120.4	7.07, overlapped	120.1	7.06, d (7.2)
9	121.9	7.18, m	122.7	7.16, d (8.0)	122.1	7.09, d (8.1)	122.1	7.09, d (8.1)	122.2	7.11, overlapped	122.0	7.12, d (7.2)
10	113.2	7.69, d (8.4)	114.7	7.83, d (8.0)	115.1	7.50, d (8.1)	115.2	7.51, d (8.1)	115.3	7.55, d (8.3)	115.1	7.53, d (7.2)
11	135.6		137.3		136.9		136.9		137.0		137.3	
12	57.8		59.3		60.2		60.2		60.0		60.3	
13	57.7	3.24, dd (4.6, 2.8)	59.0	3.28, d (2.7)	145.5	6.15, dd (17.5, 10.7)	145.5	6.16, dd (17.8, 11.1)	145.5	6.18, dd (17.6, 10.7)	145.6	6.22, dd (17.5, 10.7)
14	45.4	2.89, t (4.6); 2.80, d (4.6, 2.8)	46.0	2.89, t (4.3); 2.83, dd (4.4, 2.7)	114.1	5.22, d (10.7); 5.19, d (17.5)	114.1	5.23, d (17.8); 5.20, d (11.1)	114.1	5.25, d (10.7); 5.21, d (17.6)	114.0	5.25, d (10.7); 5.22, d (17.5)
15	23.0	1.55, s	23.2	1.55, s	28.4	1.72, s	28.4	1.72, s	28.5	1.74, s	28.3	1.78, s
16	24.3	1.62, s	25.1	1.70, s	28.4	1.72, s	28.4	1.72, s	28.5	1.74, s	28.4	1.79, s
$\Delta^{4,5}$-NorLeu												
17	171.5 or 171.4		173.3		173.4		173.5 or 173.4		173.4			
18	52.2	4.65, overlapped	53.5	4.64, m	57.1	4.68, m	56.9	4.80, m	53.4	4.61, overlapped		
19	33.4	2.68, m; 2.59, m	36.1	2.78, m; 2.51, m	35.4	2.82, m; 2.64, m	37.7	2.60, m; 2.40, m	36.2	2.80, m; 2.53, m		
20	125.6	5.39, m	125.4	5.25, m	127.6	5.60, m	127.5	5.50, m	125.5	5.20, m		
21	128.7	5.60, m	131.4	5.55, m	129.4	5.64, m	129.4	5.60, m	131.3	5.53, m		
22	18.3	1.67, d (6.5)	18.7	1.59, d (6.7)	18.4	1.67, d (6.2)	18.5	1.69, overlapped	18.7	1.60, d (6.4)		
Leu												
23	171.5 or 171.4		173.9		173.1		173.5 or 173.4		173.9			
24	55.6	5.50, dd (11.5, 3.4)	55.0	4.66, dd (11.5, 3.4)	55.1	5.27, m	53.5	5.22, m	54.9	4.65, overlapped		
25	36.9	2.13, m; 1.72, m	45.0	1.74, m	35.8	2.64, m; 1.99, m	35.7	2.64, m; 1.89, m	44.9	1.77, m		
26	24.2	1.72, m	26.0	1.78, m	25.8	1.43, m	25.6	1.52, m	26.0	1.63, m		
27	21.4	0.98, d (6.8)	22.1	0.97, d (5.5)	21.4	0.93, d (6.8)	21.9	0.96, d (6.8)	22.1	0.97, d (5.6)		
28	23.3	0.97, d (6.8)	23.2	1.01, d (5.5)	23.5	1.01, d (6.8)	23.8	1.01, d (6.8)	23.2	1.00, d (5.6)		
Modified-NMeLeu												
29	167.8		170.9		171.9		169.8		171.0			
30	58.8	3.70, dd (9.7, 0.8)	60.1	5.01, dd (9.0, 5.1)	63.2	3.77, dd (9.5, 4.1)	60.2	3.83, dd (9.5, 4.1)	60.2	5.01, dd (11.5, 4.1)		
31	31.1	2.40, ddd (13.6, 12.9, 9.5); 1.68, overlapped	34.7	2.20, m; 1.88, m	26.7	2.30, dt (12.6, 12.2); 1.85, m	31.9	2.38, ddd (14.5, 13.8, 9.9); 1.85, m	34.9	2.36, m; 1.89, m		
32	32.3	2.63, m	38.3	2.48, m	34.2	1.96, m	33.7	1.90, m	38.5	2.50, m		

33	81.4	4.67, d (2.1)	180.3		79.4	4.80, overlapped	82.5	4.65, d (1.6)	180.0	
34	17.1	1.03, d (6.7)	19.0	1.26, d (6.8)	17.5	1.10, d (6.7)	17.4	0.99, d (6.7)	19.1	1.26, d (7.0)
N-Me	38.8	3.22, s	29.9	2.75, s	38.8	3.25, s	38.8	3.25, s	29.9	2.76, s
Ala										
35	172.1		175.0		172.5		172.9		174.9	
36	47.81	4.64, overlapped	46.9	4.84, q (6.6)	47.7	4.80, overlapped	47.8	4.80, m	46.9	4.83, dd (13.3, 6.5)
37	18.0	1.33, d (6.6)	17.0	1.30, d (6.6)	17.8	1.30, d (6.6)	17.9	1.32, d (6.6)	17.0	1.30, d (6.9)
NO_2-Tyr										
38	169.6		172.4		171.7		171.7		172.3	
39	55.7	4.86, dd (9.8, 6.7)	55.6	4.60, m	54.4	4.68, m	57.0	4.70, dd (10.2, 5.7)	55.6	4.59, overlapped
40	38.8	2.97, dd (14.0, 6.7); 2.79, dd (14.0, 9.8)	38.4	3.06, dd (13.0, 9.8); 2.73, m	38. 5	3.11, m; 2.90, m	38.7	3.11, dd (14.6, 5.7); 2.90, dd (14.6, 10.2)	38.5	3.09, dd (13.0, 9.9); 2.75, dd (13.0, 4.5)
41	128.7		129.7		129.4		130.0		130.5	
42	125.4	7.80, d (2.1)	126.8	7.80, d (2.1)	126.4	7.84, d (2.0)	126.7	7.79, d (1.6)	126.8	7.80, d (2.0)
43	133.1		135.6		135.5		135.5		135.6	
44	153.9		154.7		154.3		154.6		154.7	
45	120.2	7.07, d (8.6)	121.2	7.10, d (8.5)	121.2	7.06, d (8.7)	121.4	7.06, d (8.7)	121.3	7.10, overlapped
46	138.5	7.38, dd (8.6, 2.1)	139.2	7.43, dd (8.5, 2.1)	138.9	7.40, dd (8.7, 1.6)	138.9	7.40, dd (8.7, 1.6)	139.3	7.44, dd (8.5, 2.0)
NMeLeu										
47	167.8		169.9		169.8		169.2		169.7	
48	58.7	4.22, dd (10.7, 3.6)	59.8	4.68, dd (11.6, 2.9)	59.7	4.30, dd (10.7, 3.0)	59.6	4.26, dd (10.4, 4.1)	59.8	4.69, dd (11.8, 3.0)
49	36.4	1.60, m	38.5	1.60, m	38.4	1.54, m	37.7	1.64, m	38.4	1.63, m
50	24.2	1.16, m	25.5	1.11, m	25.6	1.00, m	25.6	1.06, m	25.5	1.10, m
51	21.4	0.23, d (6.8)	21.3	0.20, d (6.6)	21.5	0.11, d (6.5)	21.4	0.11, d (6.8)	21.1	0.20, d (6.6)
52	23.3	0.46, d (6.8)	23.3	0.40, d (6.6)	24.0	0.45, d (6.5)	23.4	0.42, d (6.8)	23.6	0.40, d (6.6)
N-Me	28.5	2.27, s	29.9	2.69, s	29.5	2.36, s	29.2	2.32, s	29.9	2.70, s

[a] Recorded in $CDCl_3$; [b] Recorded in CD_3OD.

Supplementary Table 3. Strains and plasmids used in this study.

Strains/ plasmids	Relevant phenotype	Source/ [Ref.]
**Streptomycetes**		
Streptomyces atratus SCSIO ZH16	Wild-type producer of ilamycins	This work
Δorf(-6)	*S. atratus* SCSIO ZH16 with *a* 279 bp fragment of *orf(-6)* substituted by *aac(3)IV+OriT*	This work
Δorf(-5)	*S. atratus* SCSIO ZH16 with *a* 483 bp fragment of *orf(-5)* substituted by *aac(3)IV+OriT*	This work
Δorf(-4)	*S. atratus* SCSIO ZH16 with *a* 903 bp fragment of *orf(-4)* substituted by *aac(3)IV+OriT*	This work
Δorf(-3)	*S. atratus* SCSIO ZH16 with *a* 360 bp fragment of *orf(-3)* substituted by *aac(3)IV+OriT*	This work
Δorf(-2)	*S. atratus* SCSIO ZH16 with *a* 459 bp fragment of *orf(-2)* substituted by *aac(3)IV+OriT*	This work
ΔilaC	*S. atratus* SCSIO ZH16 with a 555 bp fragment of *ilaC* substituted by *aac(3)IV+OriT*	This work
ΔlaD	*S. atratus* SCSIO ZH16 with a 858 bp fragment of *ilaD* substituted by *aac(3)IV+OriT*	This work
ΔilaE	*S. atratus* SCSIO ZH16 with a 702 bp fragment of *ilaE* substituted by *aac(3)IV+OriT*	This work
ΔilaF	*S. atratus* SCSIO ZH16 with a 132 bp fragment of *ilaF* substituted by *aac(3)IV+OriT*	This work
ΔilaG	*S. atratus* SCSIO ZH16 with *a 540 bp fragment of ilaG substituted by aac(3)IV+OriT*	This work
ΔilaH	*S. atratus* SCSIO ZH16 with a 783 bp fragment of *ilaH* substituted by *aac(3)IV+OriT*	This work
ΔilaL	*S. atratus* SCSIO ZH16 with a 993 bp fragment of *ilaL* substituted by *aac(3)IV+OriT*	This work
ΔlaM	*S. atratus* SCSIO ZH16 with a 894 bp fragment of *ilaM* substituted by *aac(3)IV+OriT*	This work
ΔilaN	*S. atratus* SCSIO ZH16 with a 936 bp fragment of *ilaM* substituted by *aac(3)IV+OriT*	This work
ΔaO	*S. atratus* SCSIO ZH16 with *a* 663 *bp fragment of ilaO substituted by aac(3)IV+OriT*	This work
ΔilaR	*S. atratus* SCSIO ZH16 with *a* 1008 *bp fragment of ilaR substituted by aac(3)IV+OriT*	This work
ΔlaS	*S. atratus* SCSIO ZH16 with *a* 2205 bp fragment of *ilaE* substituted by *aac(3)IV+OriT*	This work
Δorf(+1)	*S. atratus* SCSIO ZH16 with *a* 462 bp fragment of *orf(+1)* substituted by *aac(3)IV+OriT*	This work
Δorf(+2)	*S. atratus* SCSIO ZH16 with *a* 270 bp fragment of *orf(+2)* substituted by *aac(3)IV+OriT* cassette	This work
Δorf(+3)	*S. atratus* SCSIO ZH16 with a 447 bp fragment of *orf(+3)* substituted by *aac(3)IV+OriT*	This work
Δorf(+4)	*S. atratus* SCSIO ZH16 with a 195 bp fragment of *orf(+4)* substituted by *aac(3)IV+OriT*	This work
Δorf(+5)	*S. atratus* SCSIO ZH16 with a 534 bp fragment of *orf(+5)* substituted by *aac(3)IV+OriT*	This work
E.coli		
Bw25113	K-12 derivative: *araBAD*, *rhaBAD*	[1]
ET12567	*dam*, *dcm*, *hsdM*, *hsdS*, *hsdR, cat*^{R}, *tet*^{R}	[2]
**Plasmids**		
pIJ773	P1-FRT-oriT-*aac(3)IV*-FRT-P2	[3]
pIJ790	λ–RED (*gam bet exo*) Cml^{R} *araCrep101*^{ts}	[3]
pUZ8002	*tra*, *neo*, RP4	[4]
Cosmid 23D	A cosmid which contains partial ilamycin biosynthesis cluster	This work
Cosmid 1310B	A cosmid which contains partial ilamycin biosynthesis cluster	This work
Cosmid 201E	A cosmid which contains partial ilamycin biosynthesis cluster	This work
Cosmid 47H	A cosmid which contains partial ilamycin biosynthesis cluster	This work
pΔorf(-6)	A 279 bp fragment in *orf(-6)* in cosmid 23D was substituted by the *aac(IV)+OriT* cassette using the PCR-targeting strategy	This work
pΔorf(-5)	A 483 bp fragment in *orf(-5)* in cosmid 23D was substituted by the *aac(IV)+OriT* cassette using the PCR-targeting strategy	This work
pΔorf(-4)	A 903 bp fragment in *orf(-4)* in cosmid 23D was substituted by the *aac(IV)+OriT* cassette using the PCR-targeting strategy	This work
pΔorf(-3)	A 360 bp fragment in *orf(-3)* in cosmid 23D was substituted by the *aac(IV)+OriT* cassette using the PCR-targeting strategy	This work
pΔorf(-2)	A 459 bp fragment in *orf(-2)* in cosmid 23D was substituted by the *aac(IV)+OriT* cassette using the PCR-targeting strategy	This work
pΔilaC	A 555 bp fragment in *ilaC* in cosmid 23D was substituted by the *aac(IV)+OriT* cassette using the PCR-targeting strategy	This work
pΔilaD	A 858 bp fragment in *ilaD* in cosmid 23D was substituted by the *aac(IV)+OriT* cassette using the PCR-targeting strategy	This work

p∆ilaE	A 702 bp fragment in *ilaE* in cosmid 23D was substituted by the *aac(IV)+OriT* cassette using the PCR-targeting strategy	This work
p∆ilaF	A 540 bp fragment in *ilaF* in cosmid 1310B was substituted by the *aac(IV)+OriT* cassette using the PCR-targeting strategy	This work
p∆ilaG	A 132 bp fragment in *ilaG* in cosmid 1310B was substituted by the *aac(IV)+OriT* cassette using the PCR-targeting strategy	This work
p∆ilaH	A 783 bp fragment in *ilaH* in cosmid 1310B was substituted by the *aac(IV)+OriT* cassette using the PCR-targeting strategy	This work
p∆ilaL	A 993 bp fragment in *ilaL* in cosmid 201E was substituted by the *aac(IV)+OriT* cassette using the PCR-targeting strategy	This work
p∆ilaM	A 894 bp fragment in *ilaM* in cosmid 201E was substituted by the *aac(IV)+OriT* cassette using the PCR-targeting strategy	This work
p∆ilaN	A 936 bp fragment in *ilaN* in cosmid 201E was substituted by the *aac(IV)+OriT* cassette using the PCR-targeting strategy	This work
p∆ilaO	A 663 bp fragment in *ilaO* in cosmid 201E was substituted by the *aac(IV)+OriT* cassette using the PCR-targeting strategy	This work
p∆ilaR	A 1008 bp fragment in *ilaR* in cosmid 201E was substituted by the *aac(IV)+OriT* cassette using the PCR-targeting strategy	This work
p∆ilaS	A 2205 bp fragment in *ilaO* in cosmid 201E was substituted by the *aac(IV)+OriT* cassette using the PCR-targeting strategy	This work
p∆orf(+1)	A 462 bp fragment in *orf(+1)* in cosmid 47H was substituted by the *aac(IV)+OriT* cassette using the PCR-targeting strategy	This work
p∆orf(+2)	A 270 bp fragment in *orf(+2)* in cosmid 47H was substituted by the *aac(IV)+OriT* cassette using the PCR-targeting strategy	This work
p∆orf(+3)	A 447 bp fragment in *orf(+3)* in cosmid 47H was substituted by the *aac(IV)+OriT* cassette using the PCR-targeting strategy	This work
p∆orf(+4)	A 195 bp fragment in *orf(+4)* in cosmid 47H was substituted by the *aac(IV)+OriT* cassette using the PCR-targeting strategy	This work
p∆orf(+5)	A 534 bp fragment in *orf(+5)* in cosmid 47H was substituted by the *aac(IV)+OriT* cassette using the PCR-targeting strategy	This work

Supplementary Table 4. Deduced *orf* functions in the *ila* biosynthetic gene cluster.

Gene	Size [a]	Protein homolog and origin	Similarity /Identity [b]	Proposed function
orf(-6)	159	WP_032793446.1, *Streptomyces* sp. W007	88/82	GCN5-related N-acetyltransferase
orf(-5)	226	WP_032793446.1, *Streptomyces* sp. W007	95/94	(ArsR family transcriptional regulator)
orf(-4)	548	ADI03198.1,*Streptomyces bingchenggensis* BCW-1	76/66	pyruvate phosphate dikinase
orf(-3)	210	*WP_012184543.1, Salinispora arenicola*	57/45	transcriptional regulator
orf(-2)	401	WP_030912358.1, *Streptosporangium* amethystogenes	67/61	TetR family transcriptional regulator
orf(-1)	*93*	CAP03331.1, *Vibrio aestuarianus subsp. francensis*	45/24	RecA/RadA recombinase
ilaA	105	*WP_015338763.1, Rhizobium tropic*	42/33	LysR family transcriptional regulator
ilaB	353	AJI44174.1, *Saccharothrix algeriensis,*	65/52	*streptomycin* biosynthesis operon regulator
ilaC	267	WP_040695830.1, *Nocardia vinacea*	60/46	hydrolase
ilaD	434	WP_030722736.1, *Streptomyces* sp. NRRL F-2580,	68/55	cytochrome P450
ilaE	3109	WP_040246048., *Streptomyces albus*	58/46	type I PKS
ilaF	247	AGK25202.1, *Streptomyces griseus*	62/49	thioesterase II
ilaG	79	WP_006141080.1, *Streptomyces griseoaurantiacus*;	85/68	mbtH-like protein
ilaH	380	WP_006141080.1, *Rhodococcus rhodnii*	75/63	aminotransferase
ilaI	161	WP_016692377.1, *Rhodococcus rhodochrous*	62/47	DNA-binding protein
ilaJ	357	WP_030781544.1, *Streptomyces* sp. NRRL F-5008	76/63	ABC-transporter
ilaK	277	WP_037189465.1*, Rhodococcus fascians*	74/54	ABC-2 type transporter
ilaL	402	WP_030880111.1,*Streptomyces varsoviensis,*	69/57	cytochrome P450
ilaM	420	AY204509.1, *Streptomyces turgidiscabies* car8	60/52	nitric oxide synthase
ilaN	394	AF393159.1, *Streptomyces acidiscabies*	49/26	cytochrome P450
ilaO	373	ABW00334.1, *Salinispora arenicola CNS-205*	56/45	aromatic prenyltransferase
ilaP	163	WP_021884572.1, *Clostridium* sp. CAG:590	50/35	amidotransferase subunit A
ilaQ	296	WP_037890612.1,*Streptomyces viridochromogenes*	68/57	tetrahydromethanopterin reductase
ilaR	399	ABW00329.1, *Salinispora arenicola* CNS-205	64/51	cytochrome P450
ilaS	8022	ABW00331.1, *Salinispora arenicola* CNS-205	46/39	NRPS
ilaT	416	WP_037348038.1, *Sciscionella* sp. SE31	82/74	lactate dehydrogenase
orf(+1)	231	AIR96526.1, *Streptomyces glaucescens*	89/86	porphobilinogen deaminase 2
orf(+2)	162	WP_030053397.1, *Streptomyces peruviensis*	81/71	porphobilinogen deaminase
orf(+3)	195	WP_027942013.1, *Amycolatopsis taiwanensis*	82/71	phosphoglycerate mutase
orf(+4)	96	WP_042167841.1, *Streptomyces* sp. NBRC110035	65/55	hypothetical protein
orf(+5)	287	WP_030979091, *Streptomyces* sp. NRRL S-1824	93/89	hypothetical protein

[a] Size in units of amino acids (aa); [b] percentage of homologies

Supplementary Table 5. Primers used in this study.

Name	Sequence (5'-3')	Purpose
Screen 1-F	CCGCTTCCACCGCACATCCA	For the screening of the genomic library
Screen 1-R	CGCCACCACGATGACGACCT	
Screen 2-F	CCAGCCACCTCTTCGTAGC	For the screening of the genomic library
Screen 2-R	CGGTCCGGTCGATCTTTCC	
Screen 3-F	CGGAGCCAAGCAGGTCGTC	For the screening of the genomic library
Screen 3-R	GAGGTCAGGGAAGCGGTTCAG	
Screen 4-F	GGTCATGGTGATCGCCGAACTG	For the screening of the genomic library
Screen 4-R	ACTGCCGCTCGTCGTGGTT	
orf(-6)-delF	GACATCGCGGCAATCTCCGACGCGCTGGACCGCTTCAAC attccggggatccgtcgacc	For disrupting *orf(-6)*
orf(-6)-delR	GCGGCTGGTGCCCGGTGGATCGCAGGGGACCTCTCCCAG tgtaggctggagctgcttc	
orf(-5)-delF	GGAACCCGGGCAGCCTTCTGCCTGGCGCTGCTCGACGGC attccggggatccgtcgacc	For disrupting *orf(-5)*
orf(-5)-delR	GATGCGCGTCATCCATCCGGTGGTGAGTGCGTGGGCGCA tgtaggctggagctgcttc	
orf(-4)-delF	CTCGGGCTCACCACCGAGGCGACCACCACCCTGGCCGAC attccggggatccgtcgacc	For disrupting *orf(-4)*
orf(-4)-delR	GACCACCCCGGCGGCGGCGGCCAGGCCGTGCATGTCGAG tgtaggctggagctgcttc	
orf(-3)-delF	CTCCGCTGCATCGGCTTCTCCGGCCTCTCCGGGGTGGCC attccggggatccgtcgacc	For disrupting *orf(-3)*
orf(-3)-delR	GGGGTAGCGCTGGAAGCGGAGCAACGCCGCGGACAACTC tgtaggctggagctgcttc	
orf(-2)-delF	CGTGCCGCCGCGGCTGAGCACAGCGACTCCGCGAAGCGG attccggggatccgtcgacc	For disrupting *orf(-2)*
orf(-2)-delR	CCAGCGGTCCCGGCAGGCTCGGGCCGGCGGGACGTAGGG tgtaggctggagctgcttc	
ilaC-delF	CCGCTGGTCTACCTGTCCGGATTCACGATGACGCACACG attccggggatccgtcgacc	For disrupting *ilaC*
ilaC-delR	CCCGGCGGCGGTCTCGTCGAACATCTCCGGCGGACACAT tgtaggctggagctgcttc	
ilaD-delF	GGCGCTTTCACCGCTCCGGTGAAACGCATACGGACCCCG attccggggatccgtcgacc	For disrupting *ilaD*
ilaD-delR	CCCGAAGGTGAGGTGGGTCGCCGACCGGTCTTCTCGCAG tgtaggctggagctgcttc	
ilaE-deLF	ATGCAGTCGTTGCCGCCGGCCGGCGCCATGGTCGCCGTA attccggggatccgtcgacc	For disrupting *ilaE*
ilaE-deLR	GGAGCGCGGAGCGCCCGGTCCCGCCGACGCCCCGGCAG G tgtaggctggagctgcttc	
ilaF-delF	GTCTGCTTCCCGCACGCCGGCGGTTCGGCCGGCTCCTTC attccggggatccgtcgacc	For disrupting *ilaF*
ilaF-delR	GTGACCACCGGGAAATACCCGGAAATCGAATGCCGCATC tgtaggctggagctgcttc	
ilaG-delF	GAGGATGAAAGCGCGTCGTACGTCGTATTGGTCAACGAT attccggggatccgtcgacc	For disrupting *ilaG*
ilaG-delR	GCCCGTGTCACCGGCCGCCGTACTCATCGCGTCCACCAG tgtaggctggagctgcttc	
ilaH-delF	CCGTTCGCACCACCCGTCGCCGTCTCGGACGCGATATCC attccggggatccgtcgacc	For disrupting *ilaH*
ilaH-delR	GCAGTGCGCGGCGAAGGACTCCGCGGCCGAACCGATGGG tgtaggctggagctgcttc	
ilaL-delF	CCCGAGGTGTTGCACAATCCGATTTCCGCCTATGGGCAG attccggggatccgtcgacc	For disrupting *ilaL*
ilaL-delR	GAGGTCAGGGAAGCGGTTCAGCAGTGCGCCGAGCGCCAC tgtaggctggagctgcttc	
ilaM-delF	ATCCGCCACGAGATCCGTCAGTCCGGCAGTTATGTGCAC attccggggatccgtcgacc	For disrupting *ilaM*
ilaM-delR	TGGCAGGAAGGCGGGCGACGACTCATGGTCGTCGTAGTA tgtaggctggagctgcttc	

ilaN-delF	TACCGGCGCCTGCGTGAGACCCATCCGGTCTACTGGCAC attccggggatccgtcgacc	For disrupting *ilaN*
ilaN-delR	GTACCGCAGCAGGGCACCGAGCGTCACATGTGCCTCGAT tgtaggctggagctgcttc	
ilaO-delF	ACCCATTCATGGGGCGCGGCCACGGCCGGGGAACTGCCC attccggggatccgtcgacc	For disrupting *ilaO*
ilaO-delR	GTCGTCCGGGGCGTAGGTCCACATCGGCAGGTAGACGGT tgtaggctggagctgcttc	
ilaR-delF	TATTCCAAGGACGACGGAAAAGCACTCCAGGACTGGTTC attccggggatccgtcgacc	For disrupting *ilaR*
ilaR-delR	CAGCACCGGGAACCGTCGCAGCAGCGCGGAGAGTGCGAT tgtaggctggagctgcttc	
ilaS-delF	GCACTTCAGCACAACGCTGATCTGATGTTTGAACTGCCG attccggggatccgtcgacc	For disrupting *ilaS*
ilaS-delR	CACGTCGTTGAGCGCGGCCTCCAGCGAACCGGCGTCCAG tgtaggctggagctgcttc	
orf(+1)-delF	GCGAGATGTCCGGGCGGTCCCGGGCGGGGCCCCTGTTCA attccggggatccgtcgacc	For disrupting *orf(+1)*
orf(+1)-delR	CGTTGCCGCGTATCGGTACGCATTCCAGATGCGGGTGGG tgtaggctggagctgcttc	
orf(+2)-delF	CTGGGTGCAGGGGCGCTGGCGCTTCAGTGCCGCGAGGGC attccggggatccgtcgacc	For disrupting *orf(+2)*
orf(+2)-delR	CTGCCCGCACCCGATGCCGTCGATCAGGTCACGGGCCCC tgtaggctggagctgcttc	
orf(+3)-delF	GTCAGGCACGGCGCGGCGGACGCGTTCGGGGCGCTGACC attccggggatccgtcgacc	For disrupting *orf(+3)*
orf(+3)-delR	CTCCGCCGGCAGATGGCTCATGTCGTTGTACAACAGCAG tgtaggctggagctgcttc	
orf(+4)-delF	CAGCCCTCCGTCCAGCCGTGCTTCTTCGCGAAATGGGCC attccggggatccgtcgacc	For disrupting *orf(+4)*
orf(+4)-delR	GGCTGACAGACGATCTACACCACAGGCCCGTCGAAAGAG tgtaggctggagctgcttc	
orf(+5)-delF	CACTACCGCGGCTTCGACGGCGTGGACGGCGCCTGGGAG attccggggatccgtcgacc	For disrupting *orf(+5)*
orf(+5)-delR	GGATGGCCGCAGAGGCCCGGCACCGGCCCGCTTCAATGC tgtaggctggagctgcttc	
orf(-6)-TF	CCTGACCTGCACGCCGAATG	For verifying the disruption of *orf(-6)*
orf(-6)-TR	AGCGGACGGGAAGGACGATG	
orf(-5)-TF	CCGCCAAAGGATCAGAGTTC	For verifying the disruption of *orf(-5)*
orf(-5)-TR	TCGGCGACAATGGGAACG	
orf(-4)-TF	GGCAAGGCACTCGGTCTCGT	For verifying the disruption of *orf(-4)*
orf(-4)-TR	TCTCCGCCCGTTCCGTCAAG	
orf(-3)-TF	TCCGCTGCATCGGCTTCTCC	For verifying the disruption of *orf(-3)*
orf(-3)-TR	GCGTCGCCTTTGCTGGTTCG	
orf(-2)-TF	TCGAACTTCCCGAGCAAACG	For verifying the disruption of *orf(-2)*
orf(-2)-TR	CGGTGCGTGATGAGATCCTGT	
ilaC-TF	CGACCAGGCAGGGCACATTC	For verifying the disruption of *ilaC*
ilaC-TR	CGGCGGTCTCGTCGAACATC	
ilaD-TF	TGGGTCCGCTACCGCAATTC	For verifying the disruption of *ilaD*
ilaD-TR	ATCAGCCCGTCCTTCCACAC	
ilaE-TF	TACCCAGCCCGCACTCTTCG	For verifying the disruption of *ilaE*
ilaE-TR	AGCAGGGAACCCGGCATCAG	
ilaF-TF	GTTCCGCCTGGTCTGCTTCC	For verifying the disruption of *ilaF*
ilaF-TR	GCCACCTGGTCCATGTTCGTC	
ilaG-TF	ACAACTGCCGTAACAGAGCG	For verifying the disruption of *ilaG*
ilaG-TR	TTGTCGGGATAGCGGTTCAG	
ilaH-TF	CCGTTCGAGGATGAAAGCG	For verifying the disruption of *ilaG*

ilaH-TR	AGTGGTTGGTGTCGGGTGAG	
ilaL-TF	TCCTGTGCGGTGCTGCTGAT	For verifying the disruption of *ilaL*
ilaL-TR	GCGATTCTGCGGGCTGCTTT	
ilaM-TF	TTTCGGCGAGTGAGGGAGGT	For verifying the disruption of *ilaM*
ilaM-TR	CGGGTCATCAGCCAGGAGTC	
ilaN-TF	TGAGTCGTCGCCCGCCTTC	For verifying the disruption of *ilaN*
ilaN-TR	CCGCTCGCCGTCTTCATCG	
ilaO-TF	CGATGAAGACGGCGAGCGG	For verifying the disruption of *ilaO*
ilaO-TR	GTGAGAAGTACGCCTTGAGCC	
ilaR-TF	CGTTAGCAGCGGAGTCGGAATC	For verifying the disruption of *ilaR*
ilaR-TR	GGTGGATGCGGTGAGGAGGAA	
ilaS-TF	GCACCCGCTGATAAGGCTGTC	For verifying the disruption of *ilaS*
ilaS-TR	CAGGTCCGTGAGGCGAGTCT	
orf(+1)-TF	TTGTCCATGGCCCCTCCTTC	For verifying the disruption of *orf(+1)*
orf(+1)-TR	TGCCTCGATCAGCTCGGTGT	
orf(+2)-TF	TACGCGGCAACGCCAATCG	For verifying the disruption of *orf(+2)*
orf(+2)-TR	GGGCTGAGCTGTGGAAACGC	
orf(+3)-TF	AGCGATCTTGTGGGTTCAGG	For verifying the disruption of *orf(+3)*
orf(+3)-TR	ACAGAGCGGATTCCGTGGTG	
orf(+4)-TF	GGGAGCATCACCACGGAATC	For verifying the disruption of *orf(+4)*
orf(+4)-TR	TGCGCTGGTCGGTCACTTC	
orf(+5)-TF	TTTCGACGGGCCTGTGGTG	For verifying the disruption of *orf(+5)*
orf(+5)-TR	CGCAAGGGCGATGGAGATG	

Supplementary Table 6. The cytotoxic activities of compounds **1–8** (IC_{50}, μM) [a].

	HeLa	HepG2	A549	CNE2	MCF7	L02	Huvec-12
**1**	>50	>50	>50	>50	>50	>50	>50
**2**	7.1	8.9	13.8	24.5	17.4	24.6	18.6
**3/4**	3.3	3.2	3.5	6.8	4.5	7.4	33.8
**5**	>50	>50	>50	>50	>50	>50	>50
**6/7**	3.9	4.9	6.2	14.9	3.9	14.9	18.6
**8**	>50	>50	>50	>50	>50	>50	>50
**Dox**[b]	0.98	1.2	3.4	3.6	3.4	13	34.3
**Cis**[c]	5.2	2.5	9.3	12.9	14.09	22.7	34.5

[a] HeLa, human cervical carcinoma cell line; HepG2, human hepatocellular carcinoma cell line; A549, human lung cancer cell line; CNE2, human nasopharyngeal carcinoma cell line; MCF7, human breast adenocarcinoma cell line; L02, normal human hepatic cell line; Huvec-12, normal human umbilical vein endothelial cell line.
[b] Doxorubicin.
[c] Cisplatin.

Supplementary Table 7. The antibacterial activities of compounds **1–8** against six kinds of bacteria (MICs, μM).

	Escherichia coli ATCC 25922	*Acinetobacter baumanii* ATCC19606	*Enterococcus faecalis* ATCC29212	*Staphylococcus aureus* ATCC29213	MRSA shha-01	*Micrococcus luteus* SC-1
**1**	>126.5	126.5	>126.5	>126.5	>126.5	>126.5
**2**	>124.5	124.5	>124.5	>124.5	>124.5	>124.5
**3/4**	>122.9	122.9	>122.9	>122.9	>122.9	>122.9
**5**	>121.0	121.0	>121.0	>121.0	>121.0	>121.0
**6/7**	>124.8	124.8	>124.8	>124.8	>124.8	>124.8
**8**	>122.9	122.9	>122.9	>122.9	>122.9	>122.9
Kanamycin	1.7	3.4	N/A	0.85	N/A	0.85

Supplementary References

1. Datsenko, K. A. & Wanner, B. L. One-step inactivation of chromosomal genes in Escherichia coli K-12 using PCR products. *Proc. Natl. Acad. Sci. U. S. A.* **97**, 6640-6645 (2000).
2. MacNeil, D. J. *et al*. Analysis of Streptomyces avermitilis genes required for avermectin biosynthesis utilizing a novel integration vector. *Gene* **111**, 61-68 (1992).
3. Gust, B., Challis, G. L., Fowler, K., Kieser, T. & Chater, K. F. PCR-targeted Streptomyces gene replacement identifies a protein domain needed for biosynthesis of the sesquiterpene soil odor geosmin. *Proc. Natl. Acad. Sci. U. S. A.* **100**, 1541-1546 (2003).
4. Paget, M. S., Chamberlin, L., Atrih, A., Foster, S. J. & Buttner, M. J. Evidence that the extracytoplasmic function sigma factor sigmaE is required for normal cell wall structure in *Streptomyces coelicolor* A3(2). *J. Bacteriol.* **181**, 204-211 (1999).
