# Supplementary material for: Biosynthesis of ilamycins featuring unusual building blocks and engineered production of enhanced anti-tuberculosis agents
Source: Nat Commun. 2017 Aug 30;8:391. doi: 10.1038/s41467-017-00419-5 (PMC5577134; doi:10.1038/s41467-017-00419-5)

checkCIF/PLATON report (Compound 2)

Structure factors have been supplied for datablock(s) 16c1_sq

THIS REPORT IS FOR GUIDANCE ONLY. IF USED AS PART OF A REVIEW PROCEDURE FOR PUBLICATION, IT SHOULD NOT REPLACE THE EXPERTISE OF AN EXPERIENCED CRYSTALLOGRAPHIC REFEREE.

No syntax errors found. CIF dictionary Interpreting this report

Datablock: 16c1_sq

```
Bond precision:  C-C = 0.0072 A          Wavelength=0.71073

Cell:            a=18.3538(2)     b=28.2850(3)     c=13.3201(1)
                 alpha=90         beta=90          gamma=90
Temperature:     293 K

                Calculated                  Reported
Volume          6914.96(12)                 6914.96(12)
Space group     P 21 21 2                   P 21 21 2
Hall group      P 2 2ab                     P 2 2ab
Moiety formula  C54 H77 N9 O11 [+ solvent]  C54 H77 N9 O11
Sum formula     C54 H77 N9 O11 [+ solvent]  C54 H77 N9 O11
Mr              1028.25                     1028.24
Dx,g cm-3       0.988                       0.988
Z               4                           4
Mu (mm-1)       0.070                       0.070
F000            2208.0                      2208.0
F000'           2208.99
h,k,lmax        22,34,16                    21,34,16
Nref            13571[ 7452]                13266
Tmin,Tmax       0.983,0.993                 0.355,1.000
Tmin'           0.979

Correction method= # Reported T Limits: Tmin=0.355 Tmax=1.000
AbsCorr = MULTI-SCAN

Data completeness= 1.78/0.98      Theta(max)= 25.979

R(reflections)= 0.0668( 11555)    wR2(reflections)= 0.2098( 13266)

S = 1.037                  Npar= 668
```

The following ALERTS were generated. Each ALERT has the format
test-name_ALERT_alert-type_alert-level.
Click on the hyperlinks for more details of the test.

Alert level B

```
PLAT035_ALERT_1_B _chemical_absolute_configuration info  Not given     Please Do !
PLAT230_ALERT_2_B Hirshfeld Test Diff for    C42    --  C43     ..        8.3 s.u.
PLAT410_ALERT_2_B Short Intra H...H Contact  H2A    ..  H48     ..       1.89 Ang.
PLAT410_ALERT_2_B Short Intra H...H Contact  H30    ..  H36     ..       1.86 Ang.
```

Alert level C

```
ABSTY02_ALERT_1_C  An _exptl_absorpt_correction_type has been given without
            a literature citation. This should be contained in the
            _exptl_absorpt_process_details field.
            Absorption correction given as Multi-scan
STRVA01_ALERT_4_C           Flack parameter is too small
           From the CIF: _refine_ls_abs_structure_Flack   -1.900
           From the CIF: _refine_ls_abs_structure_Flack_su    0.500
PLAT094_ALERT_2_C Ratio of Maximum / Minimum Residual Density ....       2.18 Report
PLAT242_ALERT_2_C Low    'MainMol' Ueq as Compared to Neighbors of         N7 Check
PLAT242_ALERT_2_C Low    'MainMol' Ueq as Compared to Neighbors of        C26 Check
PLAT340_ALERT_3_C Low Bond Precision on  C-C Bonds ...............    0.00715 Ang.
PLAT360_ALERT_2_C Short  C(sp3)-C(sp3) Bond   C31    -   C32    ..       1.43 Ang.
PLAT369_ALERT_2_C Long    C(sp2)-C(sp2) Bond  C42    -   C43    ..       1.53 Ang.
PLAT397_ALERT_2_C Deviating  C-O-C  Angle from 120 Deg for  O2           61.0 Degree
PLAT420_ALERT_2_C D-H Without Acceptor       N6     --  H6     ...     Please Check
PLAT716_ALERT_1_C H...A   Unknown or Inconsistent Label ..........      O6_$1
                    H2     O6_$1
PLAT717_ALERT_1_C D...A   Unknown or Inconsistent Label ..........      O6_$1
                    N2     O6_$1
PLAT718_ALERT_1_C D-H..A  Unknown or Inconsistent Label ..........      O6_$1
                    N2     H2     O6_$1
PLAT911_ALERT_3_C Missing # FCF Refl Between THmin & STh/L=  0.600          3 Report
PLAT978_ALERT_2_C Number C-C Bonds with Positive Residual Density.          0 Note
```

Alert level G

```
PLAT007_ALERT_5_G Number of Unrefined Donor-H Atoms ..............          6 Report
PLAT013_ALERT_1_G N.O.K. _shelx_hkl_checksum found in CIF ........     Please Check
PLAT032_ALERT_4_G Std. Uncertainty on Flack Parameter Value High .      0.500 Report
PLAT072_ALERT_2_G SHELXL First  Parameter in WGHT  Unusually Large       0.14 Report
PLAT143_ALERT_4_G s.u. on c - Axis Small or Missing ..............    0.00010 Ang.
PLAT199_ALERT_1_G Reported _cell_measurement_temperature ..... (K)        293 Check
PLAT200_ALERT_1_G Reported   _diffrn_ambient_temperature ..... (K)        293 Check
PLAT335_ALERT_2_G Check Large C6 Ring    C-C Range C41    -C46           0.21 Ang.
PLAT380_ALERT_4_G Incorrectly? Oriented X(sp2)-Methyl Moiety .....        C54 Check
PLAT380_ALERT_4_G Incorrectly? Oriented X(sp2)-Methyl Moiety .....        C53 Check
PLAT380_ALERT_4_G Incorrectly? Oriented X(sp2)-Methyl Moiety .....        C22 Check
PLAT606_ALERT_4_G VERY LARGE Solvent Accessible VOID(S) in Structure        ! Info
PLAT791_ALERT_4_G The Model has Chirality at C2      (Chiral SPGR)          S Verify
PLAT791_ALERT_4_G The Model has Chirality at C13     (Chiral SPGR)          S Verify
PLAT791_ALERT_4_G The Model has Chirality at C18     (Chiral SPGR)          S Verify
PLAT791_ALERT_4_G The Model has Chirality at C24     (Chiral SPGR)          S Verify
PLAT791_ALERT_4_G The Model has Chirality at C30     (Chiral SPGR)          S Verify
PLAT791_ALERT_4_G The Model has Chirality at C36     (Chiral SPGR)          S Verify
PLAT791_ALERT_4_G The Model has Chirality at C39     (Chiral SPGR)          S Verify
PLAT791_ALERT_4_G The Model has Chirality at C48     (Chiral SPGR)          S Verify
PLAT869_ALERT_4_G ALERTS Related to the use of SQUEEZE Suppressed           ! Info
PLAT912_ALERT_4_G Missing # of FCF Reflections Above STh/L=  0.600         83 Note
PLAT913_ALERT_3_G Missing # of Very Strong Reflections in FCF ....          2 Note
```

0 **ALERT level A** = Most likely a serious problem - resolve or explain
4 **ALERT level B** = A potentially serious problem, consider carefully

```
15 ALERT level C = Check. Ensure it is not caused by an omission or oversight
23 ALERT level G = General information/check it is not something unexpected

 8 ALERT type 1 CIF construction/syntax error, inconsistent or missing data
13 ALERT type 2 Indicator that the structure model may be wrong or deficient
 3 ALERT type 3 Indicator that the structure quality may be low
17 ALERT type 4 Improvement, methodology, query or suggestion
 1 ALERT type 5 Informative message, check
```

It is advisable to attempt to resolve as many as possible of the alerts in all categories. Often the minor alerts point to easily fixed oversights, errors and omissions in your CIF or refinement strategy, so attention to these fine details can be worthwhile. In order to resolve some of the more serious problems it may be necessary to carry out additional measurements or structure refinements. However, the purpose of your study may justify the reported deviations and the more serious of these should normally be commented upon in the discussion or experimental section of a paper or in the "special_details" fields of the CIF. checkCIF was carefully designed to identify outliers and unusual parameters, but every test has its limitations and alerts that are not important in a particular case may appear. Conversely, the absence of alerts does not guarantee there are no aspects of the results needing attention. It is up to the individual to critically assess their own results and, if necessary, seek expert advice.

Publication of your CIF in IUCr journals

A basic structural check has been run on your CIF. These basic checks will be run on all CIFs submitted for publication in IUCr journals (*Acta Crystallographica*, *Journal of Applied Crystallography*, *Journal of Synchrotron Radiation*); however, if you intend to submit to *Acta Crystallographica Section C* or *E* or *IUCrData*, you should make sure that full publication checks are run on the final version of your CIF prior to submission.

Publication of your CIF in other journals

Please refer to the *Notes for Authors* of the relevant journal for any special instructions relating to CIF submission.

PLATON version of 24/11/2016; check.def file version of 23/11/2016

Datablock 16c1_sq - ellipsoid plot

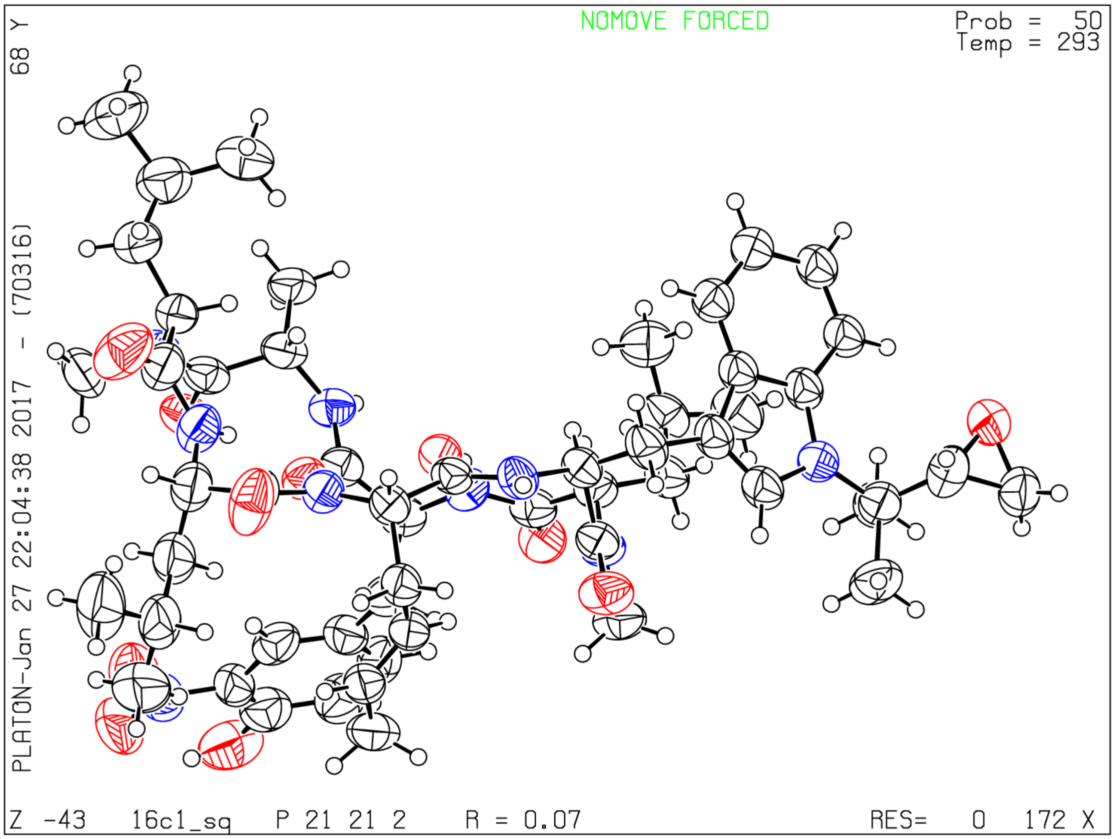

checkCIF/PLATON report (Compound 4)

Structure factors have been supplied for datablock(s) 16e

THIS REPORT IS FOR GUIDANCE ONLY. IF USED AS PART OF A REVIEW PROCEDURE FOR PUBLICATION, IT SHOULD NOT REPLACE THE EXPERTISE OF AN EXPERIENCED CRYSTALLOGRAPHIC REFEREE.

No syntax errors found. CIF dictionary Interpreting this report

Datablock: 16e

```
Bond precision:  C-C = 0.0053 A          Wavelength=1.54184

Cell:           a=17.30911(13)    b=17.89029(14)    c=21.67363(19)
                alpha=90          beta=90           gamma=90
Temperature:    293 K
```

	Calculated	Reported
Volume	6711.57(9)	6711.56(9)
Space group	P 21 21 21	P 21 21 21
Hall group	P 2ac 2ab	P 2ac 2ab
Moiety formula	C54 H75 N9 O12, 2(C H Cl3), 2(C H4 O)	C54 H75 N9 O12, 2(C H Cl3), 2(C H4 O)
Sum formula	C58 H85 Cl6 N9 O14	C58 H85 Cl6 N9 O14
Mr	1345.05	1345.04
Dx,g cm-3	1.331	1.331
Z	4	4
Mu (mm-1)	2.892	2.892
F000	2840.0	2840.0
F000’	2856.47	
h,k,lmax	21,22,26	21,22,26
Nref	13183[7215]	13122
Tmin,Tmax	0.525,0.561	0.811,1.000
Tmin’	0.400	

```
Correction method= # Reported T Limits: Tmin=0.811 Tmax=1.000
AbsCorr = MULTI-SCAN

Data completeness= 1.82/1.00      Theta(max)= 71.902

R(reflections)= 0.0458( 12503)    wR2(reflections)= 0.1273( 13122)

S = 1.023                  Npar= 810
```

The following ALERTS were generated. Each ALERT has the format
test-name_ALERT_alert-type_alert-level.
Click on the hyperlinks for more details of the test.

Alert level B

```
PLAT035_ALERT_1_B _chemical_absolute_configuration info  Not given     Please Do !
PLAT410_ALERT_2_B Short Intra H...H Contact  H2A     ..  H48      ..      1.87 Ang.
PLAT420_ALERT_2_B D-H Without Acceptor      >O1SB   -- >H1SB   ...     Please Check
```

Alert level C

```
ABSTY02_ALERT_1_C  An _exptl_absorpt_correction_type has been given without
            a literature citation. This should be contained in the
            _exptl_absorpt_process_details field.
            Absorption correction given as Multi-scan
PLAT213_ALERT_2_C Atom O12             has ADP max/min Ratio .....        3.7 prolat
PLAT220_ALERT_2_C Non-Solvent Resd 1   C   Ueq(max)/Ueq(min) Range        4.2 Ratio
PLAT220_ALERT_2_C Non-Solvent Resd 1   O   Ueq(max)/Ueq(min) Range        4.1 Ratio
PLAT222_ALERT_3_C Non-Solvent Resd 1   H Uiso(max)/Uiso(min) Range        4.8 Ratio
PLAT243_ALERT_4_C High   'Solvent' Ueq as Compared to Neighbors of        C3S Check
PLAT244_ALERT_4_C Low    'Solvent' Ueq as Compared to Neighbors of        C2S Check
PLAT340_ALERT_3_C Low Bond Precision on  C-C Bonds ...............    0.00535 Ang.
PLAT397_ALERT_2_C Deviating  C-O-C  Angle from 120 Deg for  O2           61.3 Degree
PLAT397_ALERT_2_C Deviating  C-O-C  Angle from 120 Deg for  O2S          41.7 Degree
PLAT415_ALERT_2_C Short Inter D-H..H-X       H1SB    ..  H37B     ..      2.10 Ang.
PLAT601_ALERT_2_C Structure Contains Solvent Accessible VOIDS of .          36 Ang3
PLAT790_ALERT_4_C Centre of Gravity not Within Unit Cell: Resd.  #          1 Note
              C54 H75 N9 O12
```

Alert level G

```
PLAT003_ALERT_2_G Number of Uiso or Uij Restrained non-H Atoms ...          2 Report
PLAT007_ALERT_5_G Number of Unrefined Donor-H Atoms ..............         10 Report
PLAT142_ALERT_4_G s.u. on b - Axis Small or Missing ..............    0.00014 Ang.
PLAT143_ALERT_4_G s.u. on c - Axis Small or Missing ..............    0.00019 Ang.
PLAT171_ALERT_4_G The CIF-Embedded .res File Contains EADP Records          2 Report
PLAT186_ALERT_4_G The CIF-Embedded .res File Contains ISOR Records          2 Report
PLAT199_ALERT_1_G Reported _cell_measurement_temperature ..... (K)        293 Check
PLAT200_ALERT_1_G Reported   _diffrn_ambient_temperature ..... (K)        293 Check
PLAT302_ALERT_4_G Anion/Solvent/Minor-Residue Disorder (Resd  4)..         50 % Note
PLAT302_ALERT_4_G Anion/Solvent/Minor-Residue Disorder (Resd  5)..         50 % Note
PLAT431_ALERT_2_G Short Inter HL..A  Contact Cl1S    ..  O3       ..      2.99 Ang.
PLAT720_ALERT_4_G Number of Unusual/Non-Standard Labels ..........         20 Note
PLAT790_ALERT_4_G Centre of Gravity not Within Unit Cell: Resd.  #          2 Note
              C H Cl3
PLAT790_ALERT_4_G Centre of Gravity not Within Unit Cell: Resd.  #          3 Note
              C H Cl3
PLAT790_ALERT_4_G Centre of Gravity not Within Unit Cell: Resd.  #          4 Note
              C H4 O
PLAT790_ALERT_4_G Centre of Gravity not Within Unit Cell: Resd.  #          5 Note
              C H4 O
PLAT791_ALERT_4_G The Model has Chirality at C2      (Chiral SPGR)          S Verify
PLAT791_ALERT_4_G The Model has Chirality at C13     (Chiral SPGR)          S Verify
PLAT791_ALERT_4_G The Model has Chirality at C18     (Chiral SPGR)          S Verify
PLAT791_ALERT_4_G The Model has Chirality at C24     (Chiral SPGR)          S Verify
PLAT791_ALERT_4_G The Model has Chirality at C30     (Chiral SPGR)          S Verify
PLAT791_ALERT_4_G The Model has Chirality at C32     (Chiral SPGR)          S Verify
PLAT791_ALERT_4_G The Model has Chirality at C33     (Chiral SPGR)          S Verify
PLAT791_ALERT_4_G The Model has Chirality at C36     (Chiral SPGR)          S Verify
PLAT791_ALERT_4_G The Model has Chirality at C39     (Chiral SPGR)          S Verify
```

```
PLAT791_ALERT_4_G The Model has Chirality at C48     (Chiral SPGR)          S Verify
PLAT860_ALERT_3_G Number of Least-Squares Restraints .............         12 Note
PLAT912_ALERT_4_G Missing # of FCF Reflections Above STh/L=  0.600         31 Note
PLAT978_ALERT_2_G Number C-C Bonds with Positive Residual Density.          3 Note
```

```
   0 ALERT level A = Most likely a serious problem - resolve or explain
   3 ALERT level B = A potentially serious problem, consider carefully
  13 ALERT level C = Check. Ensure it is not caused by an omission or oversight
  29 ALERT level G = General information/check it is not something unexpected

   4 ALERT type 1 CIF construction/syntax error, inconsistent or missing data
  12 ALERT type 2 Indicator that the structure model may be wrong or deficient
   3 ALERT type 3 Indicator that the structure quality may be low
  25 ALERT type 4 Improvement, methodology, query or suggestion
   1 ALERT type 5 Informative message, check
```

It is advisable to attempt to resolve as many as possible of the alerts in all categories. Often the minor alerts point to easily fixed oversights, errors and omissions in your CIF or refinement strategy, so attention to these fine details can be worthwhile. In order to resolve some of the more serious problems it may be necessary to carry out additional measurements or structure refinements. However, the purpose of your study may justify the reported deviations and the more serious of these should normally be commented upon in the discussion or experimental section of a paper or in the "special_details" fields of the CIF. checkCIF was carefully designed to identify outliers and unusual parameters, but every test has its limitations and alerts that are not important in a particular case may appear. Conversely, the absence of alerts does not guarantee there are no aspects of the results needing attention. It is up to the individual to critically assess their own results and, if necessary, seek expert advice.

Publication of your CIF in IUCr journals

A basic structural check has been run on your CIF. These basic checks will be run on all CIFs submitted for publication in IUCr journals (*Acta Crystallographica*, *Journal of Applied Crystallography*, *Journal of Synchrotron Radiation*); however, if you intend to submit to *Acta Crystallographica Section C* or *E* or *IUCrData*, you should make sure that full publication checks are run on the final version of your CIF prior to submission.

Publication of your CIF in other journals

Please refer to the *Notes for Authors* of the relevant journal for any special instructions relating to CIF submission.

PLATON version of 24/11/2016; check.def file version of 23/11/2016

Datablock 16e - ellipsoid plot

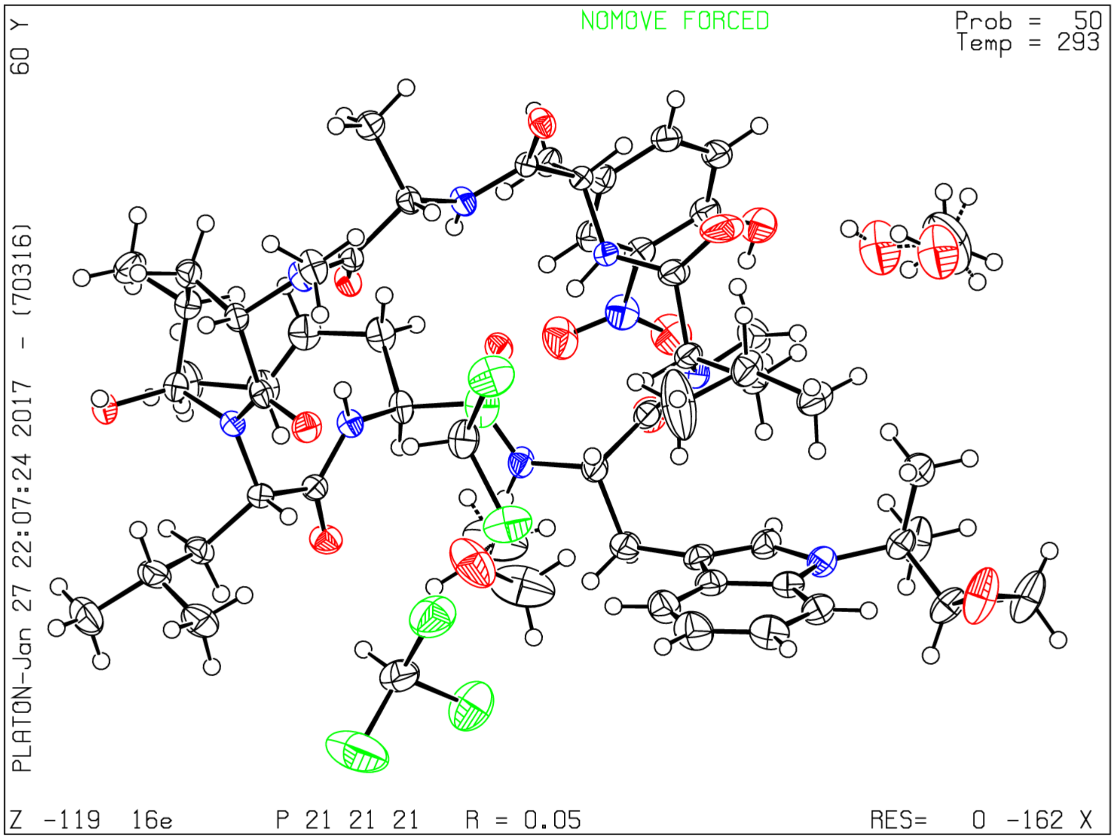

checkCIF/PLATON report (Compound 5)

Structure factors have been supplied for datablock(s) zx_16f_120305_sq

THIS REPORT IS FOR GUIDANCE ONLY. IF USED AS PART OF A REVIEW PROCEDURE FOR PUBLICATION, IT SHOULD NOT REPLACE THE EXPERTISE OF AN EXPERIENCED CRYSTALLOGRAPHIC REFEREE.

No syntax errors found. CIF dictionary Interpreting this report

Datablock: zx_16f_120305_sq

```
Bond precision:  C-C = 0.0109 A          Wavelength=1.54184

Cell:            a=15.8709(2)      b=18.8030(3)      c=22.3506(3)
                 alpha=90          beta=90           gamma=90
Temperature:     150 K

                Calculated                    Reported
Volume          6669.88(16)                   6669.88(16)
Space group     P 21 21 21                    P 21 21 21
Hall group      P 2ac 2ab                     P 2ac 2ab
Moiety formula  C54 H75 N9 O13 [+ solvent]C54 H75 N9 O13
Sum formula     C54 H75 N9 O13 [+ solvent]C54 H75 N9 O13
Mr              1058.23                       1058.23
Dx,g cm-3       1.054                         1.054
Z               4                             4
Mu (mm-1)       0.624                         0.624
F000            2264.0                        2264.0
F000'           2271.18
h,k,lmax        18,22,26                      18,22,26
Nref            11911[ 6539]                  11046
Tmin,Tmax       0.861,0.883                   0.804,1.000
Tmin'           0.829

Correction method= # Reported T Limits: Tmin=0.804 Tmax=1.000
AbsCorr = MULTI-SCAN

Data completeness= 1.69/0.93      Theta(max)= 67.078

R(reflections)= 0.0699( 7668)     wR2(reflections)= 0.2390( 11046)

S = 1.108                  Npar= 729
```

The following ALERTS were generated. Each ALERT has the format
test-name_ALERT_alert-type_alert-level.
Click on the hyperlinks for more details of the test.

Alert level B

```
PLAT035_ALERT_1_B _chemical_absolute_configuration info  Not given     Please Do !
PLAT230_ALERT_2_B Hirshfeld Test Diff for    C41    --  C46     ..        8.7 s.u.
PLAT230_ALERT_2_B Hirshfeld Test Diff for    C42    --  C43     ..       13.0 s.u.
PLAT340_ALERT_3_B Low Bond Precision on  C-C Bonds ...............    0.01091 Ang.
PLAT410_ALERT_2_B Short Intra H...H Contact  H2A    ..  H48     ..       1.84 Ang.
PLAT410_ALERT_2_B Short Intra H...H Contact  H30    ..  H36     ..       1.84 Ang.
PLAT413_ALERT_2_B Short Inter XH3 .. XHn     H3A    ..  H54A    ..       1.97 Ang.
PLAT420_ALERT_2_B D-H Without Acceptor      >O7A    -- >H7A    ...     Please Check
```

Alert level C

```
PLAT031_ALERT_4_C Refined Extinction Parameter within Range ......      3.000 Sigma
PLAT094_ALERT_2_C Ratio of Maximum / Minimum Residual Density ....       2.04 Report
PLAT220_ALERT_2_C Non-Solvent Resd 1   C   Ueq(max)/Ueq(min) Range        3.5 Ratio
PLAT222_ALERT_3_C Non-Solvent Resd 1   H Uiso(max)/Uiso(min) Range        4.4 Ratio
PLAT230_ALERT_2_C Hirshfeld Test Diff for    N7     --  C43     ..        7.0 s.u.
PLAT230_ALERT_2_C Hirshfeld Test Diff for    C40    --  C41     ..        5.5 s.u.
PLAT230_ALERT_2_C Hirshfeld Test Diff for    C50    --  C52     ..        5.2 s.u.
PLAT234_ALERT_4_C Large Hirshfeld Difference O11    --  N7      ..       0.17 Ang.
PLAT234_ALERT_4_C Large Hirshfeld Difference O12    --  C44     ..       0.17 Ang.
PLAT234_ALERT_4_C Large Hirshfeld Difference C32    --  C34B    ..       0.22 Ang.
PLAT241_ALERT_2_C High   'MainMol' Ueq as Compared to Neighbors of         O2 Check
PLAT241_ALERT_2_C High   'MainMol' Ueq as Compared to Neighbors of        C42 Check
PLAT242_ALERT_2_C Low    'MainMol' Ueq as Compared to Neighbors of        C12 Check
PLAT242_ALERT_2_C Low    'MainMol' Ueq as Compared to Neighbors of        C21 Check
PLAT242_ALERT_2_C Low    'MainMol' Ueq as Compared to Neighbors of        C43 Check
PLAT242_ALERT_2_C Low    'MainMol' Ueq as Compared to Neighbors of        C50 Check
PLAT397_ALERT_2_C Deviating  C-O-C  Angle from 120 Deg for  O2           61.2 Degree
PLAT420_ALERT_2_C D-H Without Acceptor       N6     --  H6     ...     Please Check
PLAT790_ALERT_4_C Centre of Gravity not Within Unit Cell: Resd.  #          1 Note
              C54 H75 N9 O13
PLAT911_ALERT_3_C Missing # FCF Refl Between THmin & STh/L=  0.597          9 Report
PLAT915_ALERT_3_C No Flack x Check Done: Low Friedel Pair Coverage         84 %
PLAT934_ALERT_3_C Number of (Iobs-Icalc)/SigmaW > 10 Outliers ....          1 Check
```

Alert level G

```
PLAT002_ALERT_2_G Number of Distance or Angle Restraints on AtSite          3 Note
PLAT007_ALERT_5_G Number of Unrefined Donor-H Atoms ..............          8 Report
PLAT013_ALERT_1_G N.O.K. _shelx_hkl_checksum found in CIF ........     Please Check
PLAT032_ALERT_4_G Std. Uncertainty on Flack Parameter Value High .      0.400 Report
PLAT072_ALERT_2_G SHELXL First  Parameter in WGHT  Unusually Large       0.14 Report
PLAT172_ALERT_4_G The CIF-Embedded .res File Contains DFIX Records          2 Report
PLAT301_ALERT_3_G Main Residue  Disorder ..............(Resd  1)..          4 % Note
PLAT335_ALERT_2_G Check Large C6 Ring    C-C Range C41    -C46           0.17 Ang.
PLAT432_ALERT_2_G Short Inter X...Y Contact  O1     ..  C14     ..       2.88 Ang.
PLAT432_ALERT_2_G Short Inter X...Y Contact  O10    ..  C29     ..       2.85 Ang.
PLAT432_ALERT_2_G Short Inter X...Y Contact  O10    ..  C34A    ..       2.97 Ang.
PLAT606_ALERT_4_G VERY LARGE Solvent Accessible VOID(S) in Structure        ! Info
PLAT791_ALERT_4_G The Model has Chirality at C2      (Chiral SPGR)          S Verify
PLAT791_ALERT_4_G The Model has Chirality at C13     (Chiral SPGR)          S Verify
PLAT791_ALERT_4_G The Model has Chirality at C18     (Chiral SPGR)          S Verify
PLAT791_ALERT_4_G The Model has Chirality at C24     (Chiral SPGR)          S Verify
PLAT791_ALERT_4_G The Model has Chirality at C30     (Chiral SPGR)          S Verify
PLAT791_ALERT_4_G The Model has Chirality at C36     (Chiral SPGR)          S Verify
PLAT791_ALERT_4_G The Model has Chirality at C39     (Chiral SPGR)          S Verify
PLAT791_ALERT_4_G The Model has Chirality at C48     (Chiral SPGR)          S Verify
PLAT860_ALERT_3_G Number of Least-Squares Restraints .............          2 Note
PLAT869_ALERT_4_G ALERTS Related to the use of SQUEEZE Suppressed           ! Info
PLAT978_ALERT_2_G Number C-C Bonds with Positive Residual Density.          1 Note
```

```
  0 ALERT level A = Most likely a serious problem - resolve or explain
  8 ALERT level B = A potentially serious problem, consider carefully
 22 ALERT level C = Check. Ensure it is not caused by an omission or oversight
 23 ALERT level G = General information/check it is not something unexpected

  2 ALERT type 1 CIF construction/syntax error, inconsistent or missing data
 26 ALERT type 2 Indicator that the structure model may be wrong or deficient
  7 ALERT type 3 Indicator that the structure quality may be low
 17 ALERT type 4 Improvement, methodology, query or suggestion
  1 ALERT type 5 Informative message, check
```

It is advisable to attempt to resolve as many as possible of the alerts in all categories. Often the minor alerts point to easily fixed oversights, errors and omissions in your CIF or refinement strategy, so attention to these fine details can be worthwhile. In order to resolve some of the more serious problems it may be necessary to carry out additional measurements or structure refinements. However, the purpose of your study may justify the reported deviations and the more serious of these should normally be commented upon in the discussion or experimental section of a paper or in the "special_details" fields of the CIF. checkCIF was carefully designed to identify outliers and unusual parameters, but every test has its limitations and alerts that are not important in a particular case may appear. Conversely, the absence of alerts does not guarantee there are no aspects of the results needing attention. It is up to the individual to critically assess their own results and, if necessary, seek expert advice.

Publication of your CIF in IUCr journals

A basic structural check has been run on your CIF. These basic checks will be run on all CIFs submitted for publication in IUCr journals (*Acta Crystallographica*, *Journal of Applied Crystallography*, *Journal of Synchrotron Radiation*); however, if you intend to submit to *Acta Crystallographica Section C* or *E* or *IUCrData*, you should make sure that full publication checks are run on the final version of your CIF prior to submission.

Publication of your CIF in other journals

Please refer to the *Notes for Authors* of the relevant journal for any special instructions relating to CIF submission.

PLATON version of 24/11/2016; check.def file version of 23/11/2016

Datablock zx_16f_120305_sq - ellipsoid plot

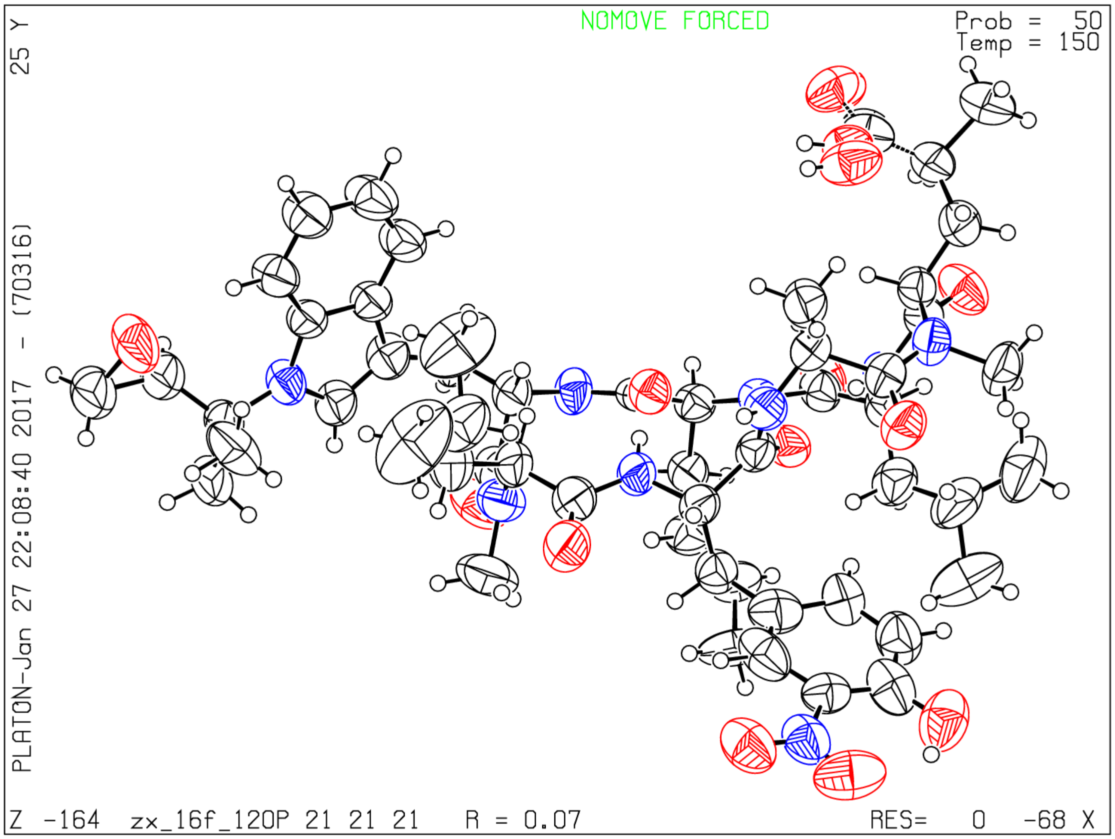

checkCIF/PLATON report (Compound 8)

Structure factors have been supplied for datablock(s) gc_8054_d_141114_sq

THIS REPORT IS FOR GUIDANCE ONLY. IF USED AS PART OF A REVIEW PROCEDURE FOR PUBLICATION, IT SHOULD NOT REPLACE THE EXPERTISE OF AN EXPERIENCED CRYSTALLOGRAPHIC REFEREE.

No syntax errors found. CIF dictionary Interpreting this report

Datablock: gc_8054_d_141114_sq

```
Bond precision:  C-C = 0.0081 A         Wavelength=1.54184

Cell:           a=17.10632(12)     b=18.5208(2)     c=39.2320(3)
                alpha=90           beta=90          gamma=90
Temperature:    293 K

                Calculated                     Reported
Volume          12429.59(19)                   12429.60(18)
Space group     P 21 21 21                     P 21 21 21
Hall group      P 2ac 2ab                      P 2ac 2ab
Moiety formula  C54 H75 N9 O12 [+ solvent]     C54 H75 N9 O12
Sum formula     C54 H75 N9 O12 [+ solvent]     C54 H75 N9 O12
Mr              1042.23                        1042.23
Dx,g cm-3       1.114                          1.114
Z               8                              8
Mu (mm-1)       0.651                          0.651
F000            4464.0                         4464.0
F000'           4477.97
h,k,lmax        20,22,46                       20,22,46
Nref            22352[ 12121]                  22349
Tmin,Tmax       0.823,0.878                    0.135,1.000
Tmin'           0.823

Correction method= # Reported T Limits: Tmin=0.135 Tmax=1.000
AbsCorr = MULTI-SCAN

Data completeness= 1.84/1.00      Theta(max)= 67.407

R(reflections)= 0.0652( 19668)    wR2(reflections)= 0.1913( 22349)

S = 1.020                   Npar= 1377
```

The following ALERTS were generated. Each ALERT has the format
test-name_ALERT_alert-type_alert-level.
Click on the hyperlinks for more details of the test.

Alert level B

```
PLAT035_ALERT_1_B _chemical_absolute_configuration info  Not given     Please Do !
PLAT097_ALERT_2_B Large Reported Max.  (Positive) Residual Density       0.88 eA-3
PLAT230_ALERT_2_B Hirshfeld Test Diff for    C42A   --  C43A    ..       15.2 s.u.
PLAT410_ALERT_2_B Short Intra H...H Contact  H30A   ..  H36A    ..       1.82 Ang.
PLAT410_ALERT_2_B Short Intra H...H Contact  H30B   ..  H36B    ..       1.87 Ang.
PLAT420_ALERT_2_B D-H Without Acceptor       O6B    --  H6B    ...     Please Check
```

Alert level C

```
DIFMX02_ALERT_1_C  The maximum difference density is > 0.1*ZMAX*0.75
            The relevant atom site should be identified.
PLAT094_ALERT_2_C Ratio of Maximum / Minimum Residual Density ....       2.52 Report
PLAT220_ALERT_2_C Non-Solvent Resd 1   C   Ueq(max)/Ueq(min) Range        3.2 Ratio
PLAT220_ALERT_2_C Non-Solvent Resd 2   C   Ueq(max)/Ueq(min) Range        3.4 Ratio
PLAT222_ALERT_3_C Non-Solvent Resd 2   H Uiso(max)/Uiso(min) Range        4.2 Ratio
PLAT230_ALERT_2_C Hirshfeld Test Diff for    O8A    --  C38A    ..        5.7 s.u.
PLAT230_ALERT_2_C Hirshfeld Test Diff for    C13A   --  C14A    ..        5.3 s.u.
PLAT230_ALERT_2_C Hirshfeld Test Diff for    C43A   --  C44A    ..        5.5 s.u.
PLAT230_ALERT_2_C Hirshfeld Test Diff for    O5B    --  C34B    ..        6.8 s.u.
PLAT234_ALERT_4_C Large Hirshfeld Difference C21A   --  C22A    ..       0.17 Ang.
PLAT241_ALERT_2_C High   'MainMol' Ueq as Compared to Neighbors of       C42A Check
PLAT242_ALERT_2_C Low    'MainMol' Ueq as Compared to Neighbors of       C43A Check
PLAT242_ALERT_2_C Low    'MainMol' Ueq as Compared to Neighbors of       C50A Check
PLAT242_ALERT_2_C Low    'MainMol' Ueq as Compared to Neighbors of       C12B Check
PLAT242_ALERT_2_C Low    'MainMol' Ueq as Compared to Neighbors of       C50B Check
PLAT340_ALERT_3_C Low Bond Precision on  C-C Bonds ...............    0.00808 Ang.
PLAT410_ALERT_2_C Short Intra H...H Contact  H2AA   ..  H48A    ..       1.91 Ang.
PLAT410_ALERT_2_C Short Intra H...H Contact  H2BA   ..  H48B    ..       1.93 Ang.
PLAT420_ALERT_2_C D-H Without Acceptor       N6A    --  H6AA   ...     Please Check
PLAT420_ALERT_2_C D-H Without Acceptor       N2B    --  H2B    ...     Please Check
PLAT430_ALERT_2_C Short Inter D...A Contact  O2B    ..  N7B     ..       2.90 Ang.
PLAT911_ALERT_3_C Missing # FCF Refl Between THmin & STh/L=  0.599          3 Report
PLAT978_ALERT_2_C Number C-C Bonds with Positive Residual Density.          0 Note
```

Alert level G

```
PLAT007_ALERT_5_G Number of Unrefined Donor-H Atoms ..............         14 Report
PLAT013_ALERT_1_G N.O.K. _shelx_hkl_checksum found in CIF ........     Please Check
PLAT072_ALERT_2_G SHELXL First  Parameter in WGHT  Unusually Large       0.13 Report
PLAT143_ALERT_4_G s.u. on c - Axis Small or Missing ..............    0.00030 Ang.
PLAT199_ALERT_1_G Reported _cell_measurement_temperature ..... (K)        293 Check
PLAT200_ALERT_1_G Reported   _diffrn_ambient_temperature ..... (K)        293 Check
PLAT380_ALERT_4_G Incorrectly? Oriented X(sp2)-Methyl Moiety .....       C22B Check
PLAT606_ALERT_4_G VERY LARGE Solvent Accessible VOID(S) in Structure        ! Info
PLAT720_ALERT_4_G Number of Unusual/Non-Standard Labels ..........         11 Note
PLAT791_ALERT_4_G The Model has Chirality at C2A     (Chiral SPGR)          S Verify
PLAT791_ALERT_4_G The Model has Chirality at C2B     (Chiral SPGR)          S Verify
PLAT791_ALERT_4_G The Model has Chirality at C18A    (Chiral SPGR)          S Verify
PLAT791_ALERT_4_G The Model has Chirality at C18B    (Chiral SPGR)          S Verify
PLAT791_ALERT_4_G The Model has Chirality at C24A    (Chiral SPGR)          S Verify
PLAT791_ALERT_4_G The Model has Chirality at C24B    (Chiral SPGR)          S Verify
PLAT791_ALERT_4_G The Model has Chirality at C30A    (Chiral SPGR)          S Verify
PLAT791_ALERT_4_G The Model has Chirality at C30B    (Chiral SPGR)          S Verify
PLAT791_ALERT_4_G The Model has Chirality at C32A    (Chiral SPGR)          S Verify
PLAT791_ALERT_4_G The Model has Chirality at C32B    (Chiral SPGR)          S Verify
PLAT791_ALERT_4_G The Model has Chirality at C36A    (Chiral SPGR)          S Verify
PLAT791_ALERT_4_G The Model has Chirality at C36B    (Chiral SPGR)          S Verify
PLAT791_ALERT_4_G The Model has Chirality at C39A    (Chiral SPGR)          S Verify
PLAT791_ALERT_4_G The Model has Chirality at C39B    (Chiral SPGR)          S Verify
PLAT791_ALERT_4_G The Model has Chirality at C48A    (Chiral SPGR)          S Verify
```

```
PLAT791_ALERT_4_G The Model has Chirality at C48B    (Chiral SPGR)          S Verify
PLAT869_ALERT_4_G ALERTS Related to the use of SQUEEZE Suppressed           ! Info
PLAT909_ALERT_3_G Percentage of Observed Data at Theta(Max) Still          69 % Note
PLAT913_ALERT_3_G Missing # of Very Strong Reflections in FCF ....          1 Note
PLAT933_ALERT_2_G Number of OMIT Records in Embedded .res File ...          2 Note
```

```
   0 ALERT level A = Most likely a serious problem - resolve or explain
   6 ALERT level B = A potentially serious problem, consider carefully
  23 ALERT level C = Check. Ensure it is not caused by an omission or oversight
  29 ALERT level G = General information/check it is not something unexpected

   5 ALERT type 1 CIF construction/syntax error, inconsistent or missing data
  25 ALERT type 2 Indicator that the structure model may be wrong or deficient
   5 ALERT type 3 Indicator that the structure quality may be low
  22 ALERT type 4 Improvement, methodology, query or suggestion
   1 ALERT type 5 Informative message, check
```

It is advisable to attempt to resolve as many as possible of the alerts in all categories. Often the minor alerts point to easily fixed oversights, errors and omissions in your CIF or refinement strategy, so attention to these fine details can be worthwhile. In order to resolve some of the more serious problems it may be necessary to carry out additional measurements or structure refinements. However, the purpose of your study may justify the reported deviations and the more serious of these should normally be commented upon in the discussion or experimental section of a paper or in the "special_details" fields of the CIF. checkCIF was carefully designed to identify outliers and unusual parameters, but every test has its limitations and alerts that are not important in a particular case may appear. Conversely, the absence of alerts does not guarantee there are no aspects of the results needing attention. It is up to the individual to critically assess their own results and, if necessary, seek expert advice.

Publication of your CIF in IUCr journals

A basic structural check has been run on your CIF. These basic checks will be run on all CIFs submitted for publication in IUCr journals (*Acta Crystallographica*, *Journal of Applied Crystallography*, *Journal of Synchrotron Radiation*); however, if you intend to submit to *Acta Crystallographica Section C* or *E* or *IUCrData*, you should make sure that full publication checks are run on the final version of your CIF prior to submission.

Publication of your CIF in other journals

Please refer to the *Notes for Authors* of the relevant journal for any special instructions relating to CIF submission.

PLATON version of 24/11/2016; check.def file version of 23/11/2016

Datablock gc_8054_d_141114_sq - ellipsoid plot

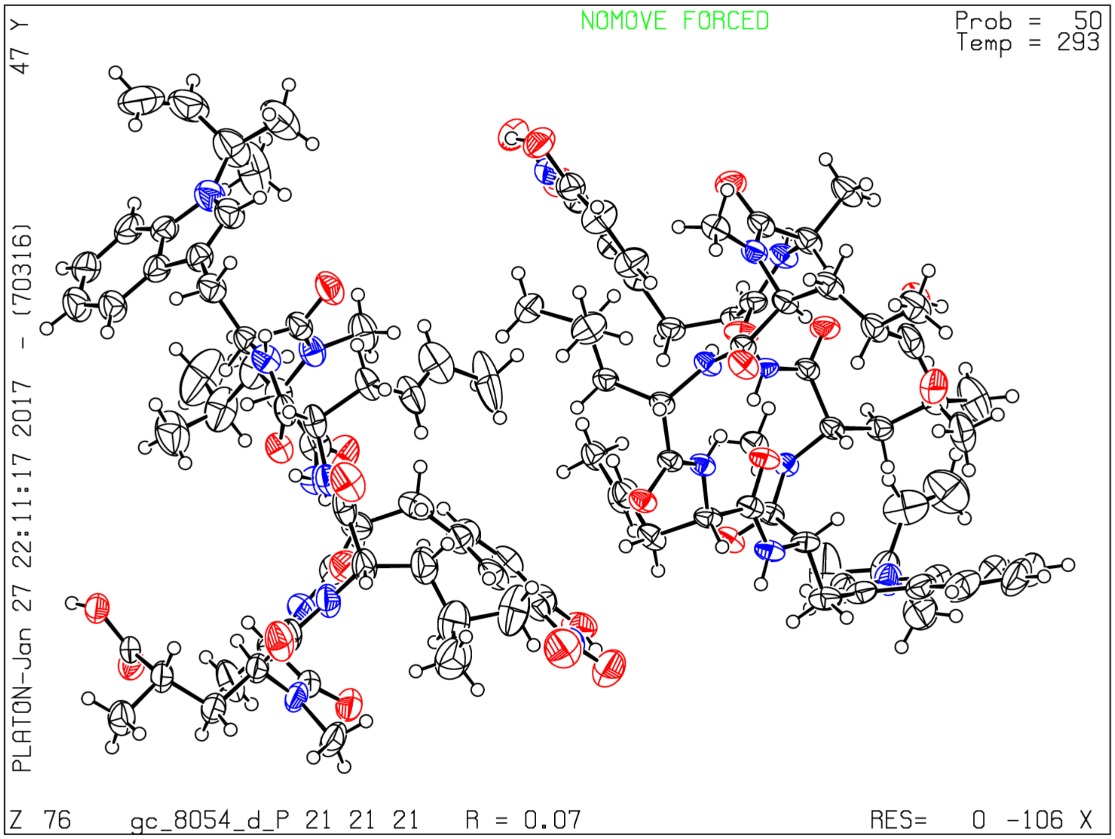

Supplement: Supplementary file 6 — Supplementary Data 5 [file 41467_2017_419_MOESM6_ESM.pdf]
